# Metabolomic and transcriptomic analyses provide insights into variations in flavonoids contents between two *Artemisia* cultivars

**DOI:** 10.1186/s12870-023-04295-8

**Published:** 2023-05-30

**Authors:** Yuchen Qiao, Liqin Wu, Suling Yang, Qing Wang, Haike Gu, Liqin Wei, Guijun Liu, Sijing Zhou, Ping Wang, Meifang Song

**Affiliations:** 1 Institute of Radiation Technology, Beijing Academy of Science and Technology, Beijing, China; 2 Beijing Science and Technology Innovation Promotion Center, Beijing, China

**Keywords:** Artemisia cultivars, Flavonoids, Metabolome, Antioxidant, RNA-seq

## Abstract

**Background:**

Plants in the genus *Artemisia* are rich in active ingredients and specialized metabolites. Many of these compounds, especially flavonoids, have potential medicinal and nutritional applications, and are of growing interest to scientists due to their wide range of pharmacological and biological activities. *Artemisia* cultivars are commonly used as raw materials for medicine, food, and moxibustion in China. However, most of the metabolites produced by *Artemisia* species have not been identified, and few studies have addressed differences in active compounds between species and cultivars.

**Results:**

We here investigated two *Artemisia* cultivars, ‘Nanyangshiyong’ (NYSY) and ‘Nanyangyaoyong’ (NYYY), which are commonly used in foods and moxibustion, respectively. NYSY and NYYY were confirmed to be* Artemisia argyi* cultivars. Total flavonoids contents and antioxidant activities were higher in NYYY than in NYSY. A total of 882 metabolites were identified in the samples; most of the potentially medicinally active compounds, especially flavonoids (e.g., flavone, flavonol, isoflavone, and anthocyanin), were up-regulated in NYYY compared to NYSY. Furthermore, most of the genes related to flavonoids biosynthesis were up-regulated in NYYY. Correlation analysis was used to identify putative members of transcription factor families that may regulate genes encoding key flavonoids biosynthesis enzymes.

**Conclusions:**

We found that the antioxidant activities and flavonoids contents significantly varied between two *Artemisia* cultivars of the same species. We also uncovered metabolomic and transcriptomic evidence of the molecular phenomena underlying those differences in flavonoids contents between the two *Artemisia* cultivars. This study provides a wealth of data for future utilization and improvements of *Artemisia* cultivars, and highlights a need to study the specific metabolite profiles of plants that are used in foods and medicines.

**Supplementary Information:**

The online version contains supplementary material available at 10.1186/s12870-023-04295-8.

## Background

The genus *Artemisia* is one of the largest genera in the family Compositae (daisy); it contains more than 500 species that are widely distributed across temperate regions in Asia, Europe, and North America [1, 2]. China alone hosts more than 180 *Artemisia* species, which belong to two subtypes: subgenus *Artemisia* and subgenus *Dracunculus* [3]. The species in these subgenera include *A.annua*, *A. argyi*, *A. lavandulaefolia, A.montana*, *A.princeps*, *A. mongolica*, *A. indica*, and *A.vulgaris* [3].

*Artemisia* plants are rich in active ingredients and specialized metabolites. These compounds are produced to protect plants against biotic or abiotic stresses, but can also combat disease in primary consumers [4]. For many years, *Artemisia* species have been used as ethnomedicines to treat ailments such as malaria, hepatitis, cancer, inflammation, and infection [5]. For example, artemisinin, the most well-known antimalarial medicine, is derived from *A. annua* [6]. Many other phytochemicals, such as essential oils [7], flavonoids [8], terpenoids [8], phenolic acids [9], lignans [10], coumarins [10], organic acids [11], alkaloids [12], and tannins [13] are found in *Artemisia* species, which are thus a rich source of potentially medicinally or nutritionally beneficial bioactive compounds.

Previous evidence shows that many ingredients in *Artemisia* plants have pharmacological activity, including antioxidant [14, 15], anti-obesity [15], anti-inflammatory [15], antifungal [16], antibacterial [17], and anticoagulation [18] effects. One class of such specialized metabolites is flavonoids, which are highly structurally variable phenols. Flavonoids such as flavones, anthocyanins, flavanones, and flavanols are of growing interest to scientists because they have a wide range of pharmacological and biological properties, including anti-cancer, anti-allergic, anti-inflammatory, antioxidant, antimicrobial, antifungal, and anti-diarrheal activities [19–21]. Many *Artemisia* cultivars are currently used as raw materials for medicines, moxa, foods, and cosmetics in China [3]. Different species or cultivars may qualitatively and quantitatively vary in the composition of bioactive compounds. For this reason, although *A. argyi*, *A. princeps*, and *A. montana* are all used as sources of material for both moxibustion and medicine in Japan [22], only *A. argyi* is used in medicines in China [6]. However, research comparing *Artemisia* cultivars has been limited. Species authentication, chemical composition profiling, and nutritional and functional property analyses have been conducted in very few *Artemisia* cultivars. Furthermore, despite the known importance of flavonoids to the medicinal and nutritional effects of many bioactive plant species, flavonoids contents and biosynthetic pathways in *Artemisia* cultivars remain largely unknown. This raises safety concerns about the use of these plants and impedes their full utilization.

We here investigated two *Artemisia* cultivars: ‘Nangyangshiyong’ (NYSY), which is commonly used in food, and ‘Nangyangyaoyong’ (NYYY), which is commonly used in moxibustion. NYSY and NYYY were validated as *A. argyi* cultivars, and we analyzed morphological traits at the vegetative growth stage. NYYY had higher total flavonoids contents and antioxidant activities than NYSY plants. A total of 882 unique metabolites were identified in the two cultivars. Most medical compounds, especially flavonoids, were up-regulated in NYYY compared to NYSY, as were most flavonoids synthesis genes. Finally, we uncovered putative transcription factors (TFs) of the differentially expressed flavonoids biosynthesis genes, including members of the R2R3-MYB, bHLH, bZIP, WRKY, NAC, and MADS TF families.

In summary, we here present comprehensive metabolomic and transcriptomic profiles of two key *Artemisia* cultivars. Our work expands current knowledge of the chemical components present in *Artemisia* cultivars and delineates the molecular and metabolic mechanisms underlying cultivar-specific flavonoids variations. The results increase our understanding of bioactive metabolite regulation in *Artemisia* species and demonstrate the necessity of thoroughly characterizing plant cultivars used in foods or medicines to ensure safety and optimize their use. Furthermore, this study lays the groundwork for future genetic improvement of members of the economically valuable *Artemisia* genus.

## Results

### NYSY and NYYY were validated as *A. argyi* cultivars

**Fig. 1:**
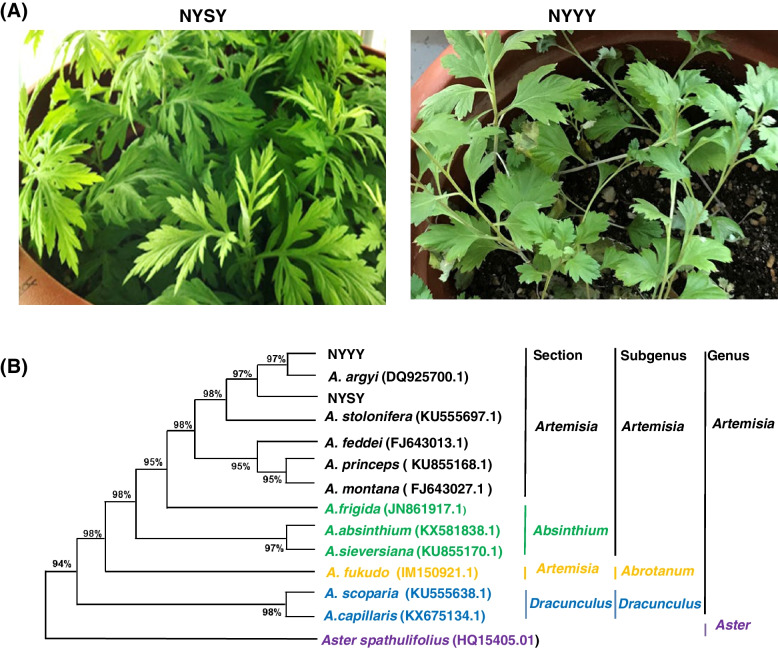
Morphological characterization and evolutionary relationships of NYSY and NYYY. **A** Morphological phenotypes of NYSY and NYYY plants grown under long day condition for two months. **B** Phylogenetic tree constructed from the internal transcribed spacer (ITS) 2 sequences of 14 plants in the family *Compositae* NYYY, NYSY, *A. argyi* (DQ925700.1), *A. stolonifera* (KU555697.1), *A. feddei* (FJ643013.1), *A. princeps* (KU855168.1), *A. montana* (FJ643027.1), *A. frigida* (JN861917.1), *A. absinthium* (KX581838.1), *A*. *sieversiana* (KU855170.1), *A.fukudo* (IM150921.1), *A. scoparia* (KU555638.1), *A.capillaris* (KX675134.1), and the out-group species *Aster spathulifolius* (HQ154050.1). *Aster spathulifolius* was chosen as an out-group species

After growing plants of both cultivars, we first analyzed the morphology of each during the vegetative growth stage. Significant morphological differences were observed; NYSY exhibited pinnately compound leaves, whereas NYSY had palmatipartite leaves (Fig. 1A). However, this did not positively confirm the species to which either cultivar belonged. Internal transcribed spacer 2 (ITS2) sequences have been used for species authentication in *Artemisa* [23–26]. We therefore analyzed ITS2 sequences of both cultivars and constructed a phylogenetic tree to show the relationships between ITS2 sequences in several *Artemisia* species. Specifically, the tree contained ITS2 sequences from NYSY, NYYY, *Aster spathulifolius*, and other Compositae species from the subgenera *Artemisia* (*A. montana*, *A. stolonifera*, *A. princeps*, *A. argyi*, and *A. feddei*), *Absinthium* (*A. frigida*, *A. absinthium*, and *A. selengensis*), *Dracunculus* (*A.scoparia* and *A. capillaris*), and *Abrotanum* (*A. fukudo*)*.* NYSY and NYYY clustered together with the members of the subgenus *Artemisia* (Fig. 1B). Furthermore, both NYSY and NYYY showed the highest similarity to *A. argyi* (GeneBank accession number: DQ925700); NYYY showed 100% sequence identity with *A. argyi*, whereas NYSY had a single degenerate site (C/G/T) at nucleotide position 107. Thus, despite the significant differences in morphological phenotypes at the vegetative growth stage, sequence analysis indicated that both cultivars were *A. argyi* (Fig. 1A).

### Metabolites with medical or nutritional properties varied between NYSY and NYYY

**Fig. 2:**
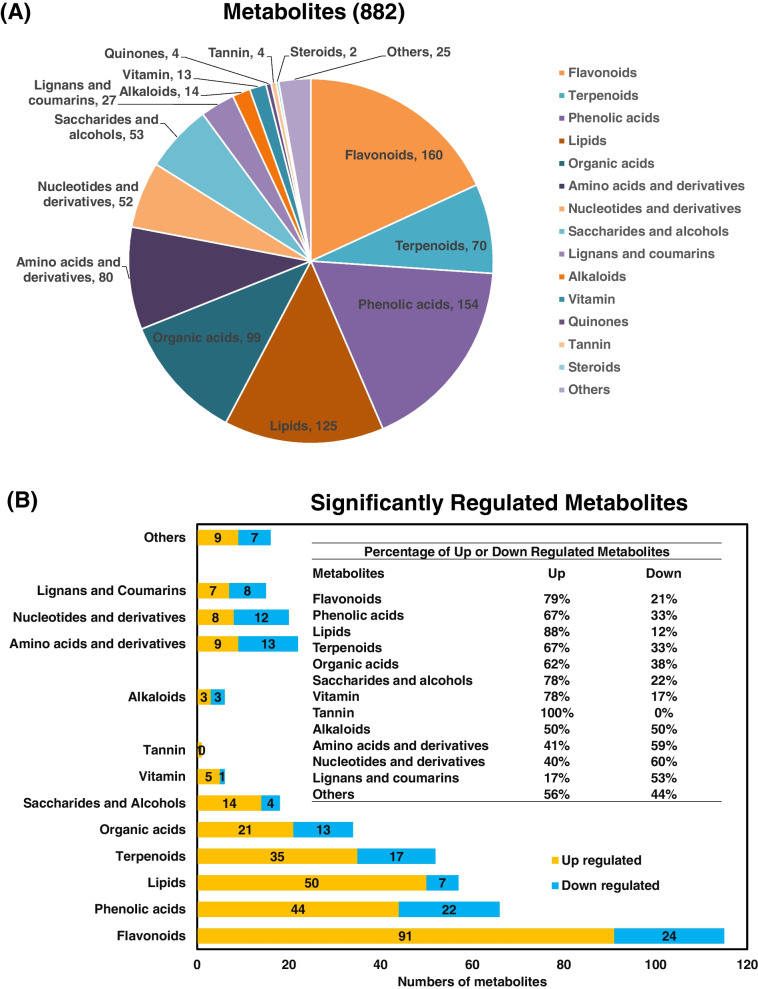
Metabolomic profiling of NYSY and NYYY. **A** Distribution of metabolites in both NYSY and NYYY cultivars. **B** Classification of significantly differentially regulated metabolites between NYSY and NYYY

To investigate the presence and abundance of potentially active ingredients in the two cultivars, metabolomic profiles were generated for each cultivar using qualitative and quantitative mass spectrometry analyses. In total, 882 metabolites were identified in the two cultivars. These comprised 160 flavonoids (specifically five chalcones, one anthocyanin, four dihydroflavones, one dihydroflavonol, 100 flavones, 22 flavonols, 14 flavonoid carbonosides, 12 isoflavones, and one sinensetin), 154 phenolic acids, 125 lipids, 99 organic acids and derivatives, 80 amino acids and derivatives, 70 terpenes, 53 saccharides and alcohols, 52 nucleotides and derivatives, 27 lignans and coumarins, 14 alkaloids, 13 vitamins, four quinones, four tannins, two steroids, and 25 other metabolites (Fig. 2A, Table S1).

We then compared the metabolite profiles of NYSY and NYYY samples to understand variations between the two cultivars. A correlation heat map showed high similarity between biological replicates (R^2^ > 0.8), demonstrating the reliability of the data (Fig. S1A). Principal component analysis (PCA) revealed that the first two principal components (PC1 and PC2) explained 68.16% and 13.84% of the variation, respectively, suggesting that most of the significant differences in metabolite contents between the two varieties were captured by PC1 (Fig. S1B). This result suggested that genetic variation strongly influenced the differing metabolite profiles.

Metabolites were classified as differentially regulated between cultivars using a fold-change (FC) thresholds of ≥ 2 (for classification as up-regulated) or ≤ 0.5 (for classification as down-regulated) and a variable importance in projection (VIP) threshold of ≥ 1. Using these thresholds, a total of 430 metabolites were differentially regulated, with 299 up-regulated and 131 down-regulated in NYYY compared to NYSY. The remaining 452 metabolites were not significantly differentially regulated (Fig. S2, Table S1). Most of the differentially regulated metabolites were significantly up-regulated in NYYY compared to NYSY. Specifically, 79% of differentially regulated flavonoids were up-regulated in NYYY, as were 67% of phenolic acids, 88% of lipids, 67% of terpenoids, 62% of organic acids, 78% of saccharides and alcohols, 83% of vitamins, and 100% of quinones. For alkaloids and lignans/coumarins, the proportions of up- and down-regulated compounds were comparable between the two cultivars, with 50% and 47%, respectively, up-regulated in NYYY. Less than half of the differentially regulated amino acids (and derivatives) and nucleotides (and derivatives) were up-regulated in NYYY (41% and 40%, respectively) (Fig. 2B). Overall, the metabolite profiles showed significant differential regulation between the two cultivars, and that most of the differentially regulated metabolites were up-regulated in NYYY.

### Flavonoids contents and antioxidant capacities varied between cultivars

An additional analysis was conducted to understand variations in flavonoids contents between NYSY and NYYY. Of the 145 significantly differentially regulated flavonoids, > 50% of the members of all classes were up-regulated in NYYY (Table 1, Table S1). Specifically, 100% of the chalcones and dihydroflavones were up-regulated, as were 91% of the isoflavones, 83% of the flavones, 54% of the flavonoid carbonosides, 54% of the flavonols, the single anthocyanin and dihydroflavonol.

**Table 1:** Significantly differentially regulated flavonoids between NYSY and NYYY

Metabolite category	Percentage up-regulated	Number up-regulated	Percentage down-regulated	Number Down-regulated
Anthocyanins	100	1	0	0
Dihydroflavonol	100	1	0	0
Chalcones	100	4	0	0
Dihydroflavone	100	4	0	0
Isoflavones	91	10	9	1
Flavones	83	57	17	12
Flavanols	54	7	46	6
Flavonoids carbonoside	54	7	46	6

“Up-regulated” and “down-regulated” refer to abundance in NYYY compared to NYSY

**Fig. 3:**
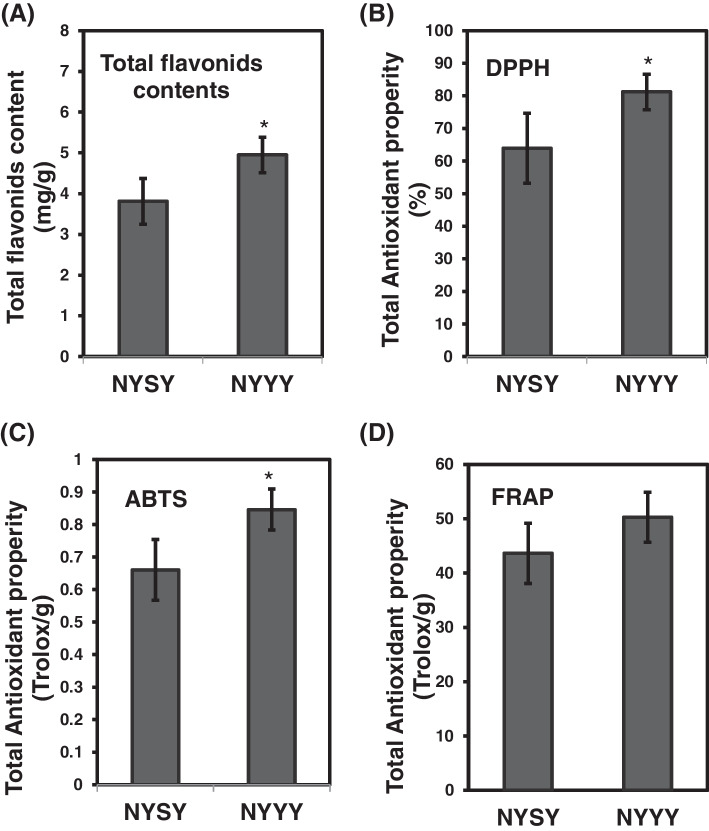
Total flavonoids contents and antioxidant activities in NYSY and NYYY. **A** Total flavonoids contents in each of the two cultivars; **B**-**D** Specific antioxidant capacities in NYSY and NYYY. **B** DPPH radical scavenging activity, **C** ABTS scavenging capacity, and **D** FRAP scavenging activity. Both cultivars were grown under 16 / 8 h light/dark condition (~ 200 μmol m^−2^ s.^−1^ light intensity) at 25 °C for two months, (*p < 0.05)

The antioxidant capacities of flavonoids are important for their applications in medicines, foods, and cosmetics. Therefore, we also analyzed the total flavonoids contents and antioxidant capacities in NYSY and NYYY to determine whether there were variations between the two cultivars. Consistent with the metabolomic data, we found that NYYY had significantly higher flavonoids contents than NYSY (5.0 and 3.8 mg/g of dry weight, respectively; *p* < 0.05) (Fig. 3A). NYYY also showed a statistically significant (*p* < 0.05) increase in the capacity to scavenge 2,2-diphenyl-1-picrylhydrazyl (DPPH) free radicals and free radical 2,2-azinobis [3-ethyl-benzothiazoline-6-sulfonic acid] (ABTS)(86.2% and 62.7 μmol Trolox/g, respectively, in NYYY, and 64% and 48.9.0 μmol Trolox/g, respectively, in NYSY), Although NYYY also showed an increase in the capacity to scavenge ferric reducing antioxidant power (FRAP) (52.7 µmol Trolox/g in NYYY and 43.6 µmol Trolox/g in NYSY),the difference was not statistically significant (Fig. 3B-D).

Thus, NYYY showed higher antioxidant capacity than NYSY did, and this capacity was positively correlated with total flavonoids contents.

### Transcriptomic analysis revealed the molecular basis of flavonoids variation

To explore the molecular basis of flavonoids diversity between the two *Artemisia* cultivars, we performed transcriptome analysis (RNA-seq). A total of 375,626 unigenes were identified among the six samples (two cultivars in biological triplicate), all of which were functionally annotated using several common databases (Materials and Methods).

**Fig. 4:**
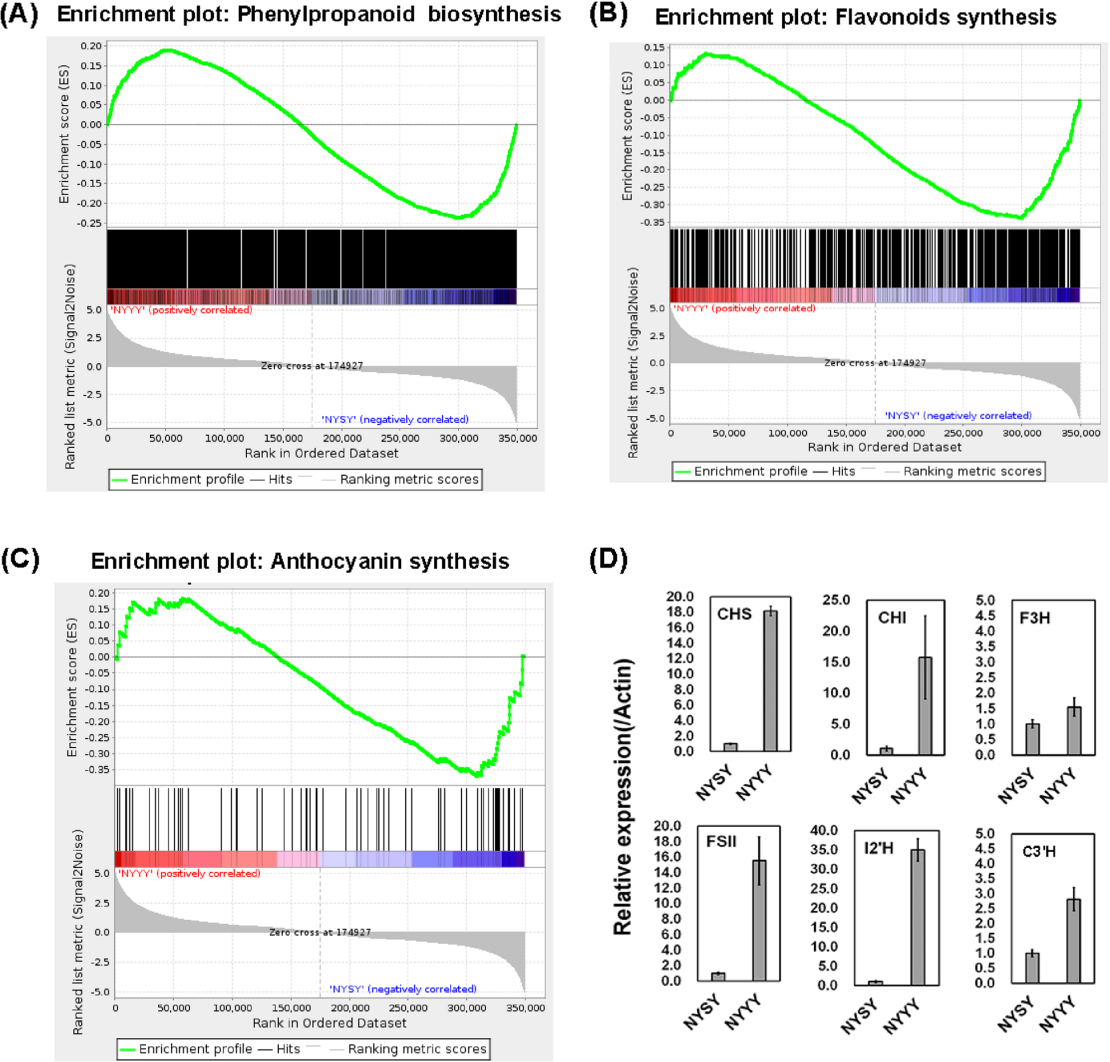
Transcriptional analysis of phenylpropanoid and flavonoids synthesis genes between NYSY and NYYY. **A**-**C** Gene set enrichment analysis (GSEA) of phenylpropanoid (**A**), flavonoids (**B**), and anthocyanin (**C**) metabolism gene sets based on pathway annotations in the Kyoto Encyclopedia of Genes and Genomes (KEGG) database. **D** Quantitative reverse transcription(qRT) PCR validation of selected candidate genes predicted to affect differential flavonoids abundance flavonoids between NYSY and NYYY. Chalcone synthase (CHS, gene cluster 12,810.170191), chalcone isomerase (CHI,gene cluster 12,810.216674), naringenin 3-dioxygenas (F3H,gene cluster 12,810.173163), flavone synthase II (FSII, gene cluster 12,810.213557), 5-O-(4-coumaroyl)-D-quinate 3'-monooxygenase (C3′H, gene Cluster-12810.193865), and I2′H (gene cluster 12,810.210239). Data represent the mean values from three biological replicates per gene in each cultivar. Error bars indicate standard error

As expected, gene set enrichment analysis (GSEA) showed that genes in the phenylpropanoid, flavonoids biosynthesis, and anthocyanin biosynthesis based on pathway annotations in the Kyoto Encyclopedia of Genes and Genomes (KEGG) database were up-regulated in NYYY compared to NYSY (Fig. 4A-C) [27]. Genes were classified as differentially expressed using FC thresholds of ≥ 4 (up-regulated) or ≤ 0.25 (down-regulated) at *p* < 0.01. A total of 45,918 differentially expressed genes (DEGs) were identified between NYSY and NYYY. Of these DEGs, 526 had the annotations “phenylpropanoid biosynthesis”, “anthocyanin biosynthesis”, “flavonoids biosynthesis”, “isoflavonoids biosynthesis”, or “flavone and flavanol biosynthesis”. Most of genes (55.3%, representing 291 clusters) encoding flavonoids synthesis enzymes were up regulated in NYYY. To validate the flavonoids synthesis genes expression levels calculated from the RNA-seq data, six DEGs associated with flavonoids biosynthesis were measured with quantitative reverse transcription (qRT)-PCR. The selected genes were those encoding a chalcone isomerase (CHI) (gene cluster 12,810.216674), a naringenin 3-dioxygenase (F3H) (gene cluster 12,810.173163), a 5-O-(4-coumaroyl)-D-quinate 3'-monooxygenase (C3'H) (gene cluster 12,810.193865), a flavone synthase II (FSII) (gene cluster 12,810.213557), a chalcone synthase (CHS) (gene cluster 12,810.170191), and an isoflavone/4'-methoxyisoflavone 2'-hydroxylase (I2′H) (gene cluster 12,810.175245). The qRT-PCR data were consistent with the RNA-seq data in both cultivars (Fig. 4D), indicating the suitability of the RNA-seq data for further analyses.

Combined with the metabolomic data, the transcriptomic data suggested that genes in the flavonoids biosynthesis pathway were differentially expressed between the two cultivars, which could explain the observed cultivar-specific variation in total flavonoids contents.

### Combined metabolomic and transcriptomic analyses demonstrated a putative flavonoids biosynthesis pathway

**Fig. 5:**
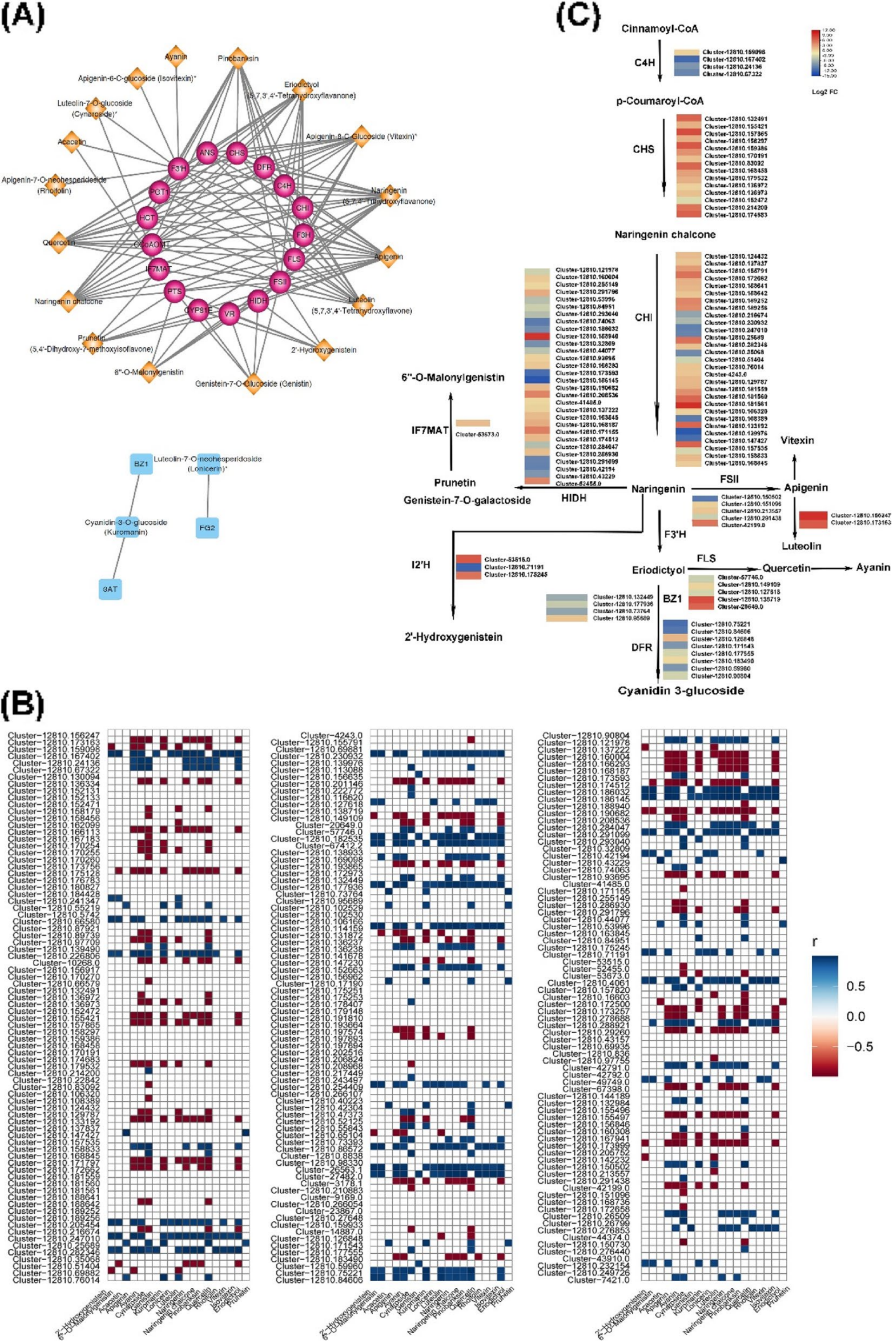
A putative flavonoids biosynthesis pathway in the two Artemisia cultivars. **A** Network analysis of 19 flavonoids ingredients (apigenin, acacetin, luteolin, ayanin, rhoifolin, cynaroside, lonicerin, pinobanksin, quercetin, naringenin, eriodictyol, naringenin chalcone, cyanidin-3-O-glucoside, isovitexin, vitexin, prunetin, 2'-hydroxygenistein, genistin, and 6''-O-malonylgenistin) and 23 flavonoids biosynthesis enzymes (C4H, trans-cinnamate 4-monooxygenase;CHS, chalcone synthase; CHI, chalcone isomerase;FSII, flavone synthase II; F3H, naringenin 3-dioxygenas; FLS, flavonol synthase; F3'H, flavonoid 3'-monooxygenase; I2'H, isoflavone/4'-methoxyisoflavone 2'-hydroxylase; HIDH, 2-hydroxyisoflavanone dehydratase; IF7MAT, isoflavone 7-O-glucoside-6''-O-malonyltransferase; BZ1, anthocyanidin3-O-glucosyltransferase; C12RT1, flavanone 7-O-glucoside 2''-O-beta-L-rhamnosyltransferase; 3AT, anthocyanidin 3-O-glucoside 6''-O-acyltransferase; ANS, ansamycin synthases; C3'H, 5-O-(4-coumaroyl)-D-quinate 3'-monooxygenase; CCoAOMT, caffeoyl-CoA O-methyltransferases; FG2, flavonol-3-O-glucoside L-rhamnosyltransferase; HCT, shikimate O-hydroxycinnamoyl transferases; PGT1, phlorizin synthases;PTS, 6-pyruvoyltetrahydropterin/6-carboxytetrahydropterin synthase; UGT75C1, anthocyanidin 3-O-glucoside 5-O-glucosyltransferase; VR, vestitone reductase; and DFR, bifunctional dihydroflavonol 4-reductase/flavanone 4-reductase); **B** Heat map showing corelations between the 19 flavonoids ingredients and expression levels of genes encoding the 23 flavonoids biosynthesis enzymes; **C** A flavonoids biosynthesis pathway potentially used by the two Artemisia cultivars based on gene expression levels and pathway annotations in the Kyoto Encyclopedia of Genes and Genomes (KEGG)database

The metabolomic and transcriptomic results discussed above indicated that flavonoids contents and flavonoids biosynthesis gene expression levels differed between *Artemisia* cultivars. To identify candidate genes involved in biosynthesis of the differentially regulated flavonoids, we conducted correlation analyses between flavonoids synthesis genes transcript and metabolites. Using correlation coefficient thresholds of ≥ 0.9 and ≤ -0.9(*p* < 0.01), we identified 238 DEGs encoding 23 flavonoids synthesis enzymes related to production of 19 flavonoids, namely seven flavones (apigenin, acacetin, luteolin, ayanin, rhoifolin, cynaroside, and lonicerin), one dihydroflavonol (pinobanksin), one flavonol (quercetin), two dihydroflavones (naringenin and eriodictyol), one chalcone (naringenin chalcone), one anthocyanin (cyanidin-3-O-glucoside), two flavonoid carbonosides (isovitexin and vitexin), and four isoflavones (prunetin, 2'-hydroxygenistein, genistin, and 6''-O-malonylgenistin) (Fig. 5A-B).

Metabolic intermediates and end products of the flavonoids biosynthesis pathway were mapped to the phenylpropane metabolism pathway, which is the starting point of flavonoids biosynthesis in plants (Fig. 5C). Consistent with the observed up-regulation of flavonoids and precursors, numerous genes in this pathway were up-regulated in NYYY (Fig. 5C). Specifically, genes encoding a trans-cinnamate 4-monooxygenase (C4H), 28 shikimate O-hydroxycinnamoyl transferases (HCTs), 26 caffeoyl-CoA O-methyltransferases (CCoAOMT), a C3'H, 14 CHSs, 23 CHIs, two F3Hs, three FSIIs, three flavonol synthases (FLSs), six phlorizin synthases (PGT1s), a flavonoids 3'-monooxygenase(F3'H), six bifunctional dihydroflavonol 4-reductase/flavanone 4-reductases (DFRs) and a flavanone 7-O-glucoside 2''-O-beta-L-rhamnosyltransferase (C12RT1) were up-regulated in NYYY (Fig. 5B-C). Members of the cyanidin biosynthesis pathway, namely one anthocyanidin synthase (ANS) and one anthocyanidin 3-O-glucosyltransferase (BZ1) gene, which are associated with cyanidin 3-glucoside, were also up-regulated in NYYY, as were members of the isoflavone synthesis pathway: 16 2-hydroxyisoflavanone dehydratases (HIDHs), two isoflavone/4'-methoxyisoflavone 2'-hydroxylases(I2'Hs), and one isoflavone 7-O-glucoside-6''-O-malonyltransferase (IF7MAT) (Fig. 5B-C). However, genes encoding other C4H, CCoAOMT, C3’H, F3'H, DFR, FLS, CHS, CHI, FSII, BZ1, HIDH, ANS, HCT, PGT1, and I2'H enzymes were down-regulated in NYYY (Fig. 5B-C). These opposing patterns between flavonoids contents and biosynthesis gene expression levels suggested the existence of multigene families that differentially controlled flavonoids biosynthesis in *Artemisia* cultivars.

### Transcriptomic analysis uncovered TFs that may regulate flavonoids biosynthesis

**Fig. 6:**
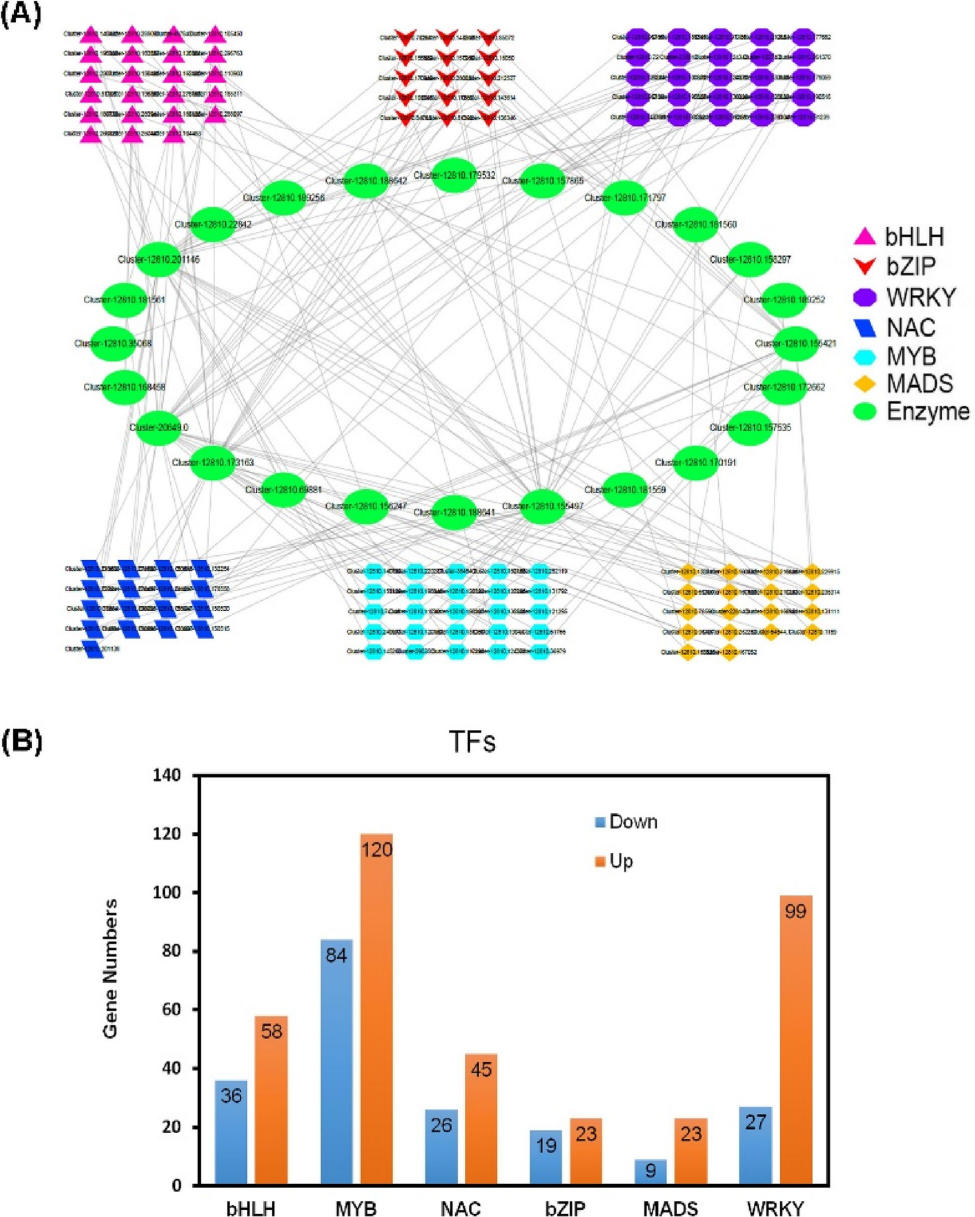
Analysis of transcription factors (TFs) that may regulate flavonoids biosynthesis genes in *Artemisia argyi.* **A** Network analysis of genes encoding five flavonoids synthesis enzymes (CHS, CHI, ANS, FLS, and F3H) and putative regulatory TFs for those genes. For this analysis, bHLH, MYB, NAC, bZIP, MADS and WRKY TFs were considered. Based on a correlation analysis between expression levels of flavonoids synthesis genes and these TFs, the five TFs of each type that were most significantly correlated with flavonoids synthesis gene expression were selected. **B** Distribution of significantly differentially expressed TFs that may regulate the five selected flavonoids synthesis enzymes (CHS, CHI, ANS, FLS, and F3H) in *A. argyi*

Our results clearly showed differential expression of genes related to flavonoids biosynthesis between the two cultivars, and indicated that genes encoding F3H, CCoAOMT, CHS, CHI, ANS, FLS, C3'H, HCT, C12RT1, DFR, HIDH, I2'H, IF7MAT, PGT1, and FSII enzymes were most likely to be responsible for flavonoids biosynthesis. Thus, we hypothesized that up-regulation of these putative synthesis genes caused the increased flavonoids content observed in NYYY compared to NYSY. We next sought to identify TFs that may regulate these flavonoids biosynthesis genes in *A. argyi*. This was accomplished by analyzing correlations between expression levels of putative TFs and flavonoids biosynthesis genes using correlation coefficient thresholds of ≥ 0.9 and ≤ -0.9, *p* < 0.01. We identified 569 TFs that may be associated with flavonoids biosynthesis: 94 bHLHs, 204 MYBs, 71 NACs, 42 bZIPs, 32 MADSs, and 126 WRKYs. These TFs were correlated with up-regulation of flavonoids synthesis genes encoding 10 CHSs, 15 CHIs, one ANS, one FLS, and two F3H enzymes. Based on the *p*-values, the five TFs in each class that were most significantly correlated with flavonoids synthesis gene expression were used in network analysis (Fig. 6A).

Consistent with the putative flavonoids biosynthesis gene expression levels, most of these TFs were up-regulated in NYYY compared to NYSY. Specifically, 62% of the bHLHs, 59% of the MYBs, 79% of the WRKYs, 63% of the NACs, 72% of the MADSs, and 55% of the bZIPs were up-regulated in NYYY (Fig. 6B). Thus, these TFs may directly or indirectly regulate expression of flavonoids biosynthesis genes, ultimately leading to the high flavonoids contents measured in NYYY plants.

## Discussion

### Species validation of NYYY and NYSY

The taxonomic relationships between members of the genus *Artemisia* are commonly based on morphological traits. However, this approach is controversial because *Artemisia* species have diverse morphological phenotypes, varying ploidy levels, and complicated genetic relationships [23]. It is thus difficult to authenticate cultivars as belonging to a particular species only through morphological traits, especially at the vegetative growth stage. Nevertheless, we here analyzed NYYY and NYSY morphology at the vegetative growth stage. As expected, we could not discern the species to which the two cultivars belonged based on morphology, which significantly varied between the two cultivars.

Another common method of plant identification involves the use of single gene regions, such as the ITS regions of ribosomal or chloroplast genes, as molecular markers [23–26]. The ITS2 sequence is reportedly appropriate for authentication of *Artemisia* species [23–26]. Indeed, in a phylogenetic analysis based on the ITS2 sequence, members of the subgenus *Artemisia* (*A. princeps*, *A. montana, A. argyi,* and *A. stolonifera*) clustered together, and NYSY and NYYY clustered with them (Fig. 1B). A degenerate site was detected in the ITS2 sequence in NYSY, likely caused by polyploidization.

### Potentially beneficial specialized metabolites in *Artemisia*

Oxidation is the main cause of cellular and therefore organismal aging. Oxidative stress is associated with the development of diseases and disorders such as cancer, cardiovascular disease, diabetes, strokes, and neurodegenerative diseases [28, 29]. Eliminating excessive oxidative free radicals in the body can prevent many such diseases. Antioxidant capacities are important in medicines, foods, and cosmetics, all of which are applications in which *Artemisia* plants are commonly used.

Metabolomic analysis here revealed 882 unique metabolites among the two *Artemisia* cultivars, including flavonoids, phenolic acids, organic acids and their derivatives, amino acids and their derivatives, terpenes, alkaloids, and lignans, some of which are potentially medicinally or nutritionally active ingredients (Fig. 2A). For example, flavonoids such as naringenin, apigenin, eriodictyol, luteolin, quercetin, and luteoloside have antioxidative, anti-inflammatory, anti-thrombotic, anti-tumor, anti-diabetic, and neuroprotective effects [21]; flavonoids are thus valuable resources for drug development. Our results suggested that the two *Artemisia* cultivars were rich in potential medicinal ingredients, and could therefore be used as a source of medicines. However, there were significant differences in metabolite profiles between the two materials. Most of the potentially medicinally active ingredients that were differentially regulated between the two cultivars were up-regulated in NYYY. These included 79% of the expressed flavonoids, 67% of the terpenoids, and 66.7% of the phenolic acids (Fig. 2B). These results indicated that NYYY is likely more suitable than NYSY for medicinal use, consistent with the current use of NYYY (but not NYSY) in moxibustion.

In addition to their medicinal uses, *Artemisia* plants also have a long history as food sources. Plant toxicity and flavor are key considerations in foods. Many *Artemisia* plants have an aromatic, astringent, bitter taste. Consumers generally find astringency and bitterness unpleasant, and polyphenols, including flavonoids, confer astringency and bitterness [30, 31]. We found that most of the flavonoids identified in the two *Artemisia* cultivars were down-regulated in NYSY (Table S1, Fig. 3C); the lower flavonoids levelmay thus increase the palatability of NYSY. This is consistent with the current use of NYSY, but not NYYY, as a food. In addition, santonin, a lactone compound long used for deworming but discontinued due to its toxicity [32], was detected in NYYY but not in NYSY (Table S1). Thus, considering flavor and toxicity, our results validate NYSY as a more suitable food source than NYYY.

Inflammation and oxidation of skin cells are the main factors leading to skin aging. Anti-inflammatory and anti-oxidation functions are therefore important components of cosmetics. Although the antioxidant activities differed between the two tested Artemisia cultivars, both had high antioxidant activities (Fig. 3B-D). Overall, the current uses of Artemisia in medicines, foods, and cosmetics were validated by the high concentrations of flavonoids identified in this study.

### Identification of flavonoids biosynthesis genes and TFs

As discussed above, flavonoids are some of the most thoroughly investigated plant specialized metabolites, and are known to be widely present in plant leaves, flowers, fruits, and other tissues [19], Flavonoids are found in many *Artemisia* species, including *A. argyi*, as demonstrated in the present study [33–38]. Here, an integrated analysis of transcriptomic and metabolomic data revealed 14 flavonoids synthesis genes that were differentially expressed between NYYY and NYSY (encoding the enzymes C4H, C3′H, HCT, FLS, F3′H, FSII, CHS, CHI, HDHF, F3′5′H, F3H, I2′H, DFR, and BZ1). The observed differences in flavonoids levels and differential expression levels of these genes indicated that they were related to flavonoids biosynthesis in *Artemisia*.

TFs are essential regulators that bind to specific DNA sequences to activate or inhibit target gene expression, thereby influencing multiple key biological processes. Studies in plants such as *Arabidopsis thaliana*, grapes, bilberry *Gerbera*, eggplant, *Populus*, and apple have confirmed that numerous activators and repressors regulate the expression of flavonoids biosynthesis genes. These TFs include members of the R2R3-MYB, NAC, bZIP, MADS-box, WRKY, and SPL families [39–46]. Research in *Arabidopsis* has shown that several flavonoids biosynthetic genes (e.g., CHS, CHI, F3H, F3′H, FLS, DFR, and ANS) are directly or indirectly regulated by R2R3-MYB TFs in the MBW complex (such as MYB75, MYB90, MYB113, MYB114, bHLH, and WD40), NACs, WRKYs, or SPLs transcription factors in response to various stimuli [39, 40, 47–54]. Differential combinatorial interactions of cis-acting elements that are recognized by R2R3-MYB, bZIP, and bHLH factors control light-responsive and tissue-specific activation of phenylpropanoid biosynthesis genes.

Similarly, many R2R3-MYB, NAC, MADS bZIP, WRKY, and SPL TFs have recently been found to participate in flavonoids biosynthesis in economically important crops such as apple, grape, *Gerbera* × *hybrida*, and eggplant [41, 45, 55–58]. We here found that seven R2R3-MYB, 94 WRKY, 57 NAC, 44 bHLH, and 20 bZIP TFs were differentially expressed between NYSY and NYYY plants (Fig. 6). Most of the differentially expressed *WRKY*, *R2R3-MYB*, *bHLH*, and *MADS* genes were up-regulated in NYYY, whereas most of the NAC and bZIP TFs were down-regulated (Fig. 6). A previous study in *Arabidopsis* showed that AtMYB11, AtMYB12 and AtMYB111 are all independently capable of activating the genes encoding CHS, CHI, F3H, and FLS, which together determine flavonol content [59]. WRKY genes function together with MYB and bHLH genes to positively or negatively regulate flavonoids synthesis. For example, PyWRKY26 and PybHLH3 co-target the PyMYB114 promoter, which results in anthocyanin accumulation in red-skinned pears [44].

These prior results suggest that the TFs we identified as differentially expressed between NYYY and NYSY may directly or indirectly coordinate the expression of flavonoids biosynthesis genes through a complex regulatory network, leading to accumulation of flavonoids such as naringenin, apigenin, eriodictyol, luteolin, quercetin, and luteoloside. The roles of these TFs in the flavonoids metabolic pathway and in regulatory responses to various stimuli require further study. However, their identification will enable future targeted breeding in *Artemisia* plants, allowing researchers to modulate and optimize flavonoids contents depending on the intended purpose of a specific cultivar.

## Conclusions

Our results shed light on the specialized metabolites present in two *A. argyi* cultivars. The differences identified between the two materials highlight the need for further understanding of the properties of individual cultivars used in medicinal and nutritional applications; NYYY was more suited to medicinal use, whereas NYSY was more appropriate for consumption in foods. Furthermore, we provide novel insights into the molecular and genetic regulatory mechanisms underlying flavonoids biosynthesis in *Artemisia* plants. Our results promote optimal utilization of *Artemisia* cultivars and will facilitate future breeding efforts in these economically valuable plants.

## Materials and methods

### Plant materials and cultivation methods

Two *Artemisia* cultivars, NYYY and NYSY, were kindly provided by Nanyang Guoyizhongjing Wormwood Industry Co. Ltd, China. The two cultivars were collected from Qiaotou, Sheqi, Nangyang, Henan, China with permission. The two materials were authenticated by Gu HK and Qiao YC and have been deposited at the Institute of Radiation Technology, Beijing Academy of Science and Technology.

All plants were grown in soil under a 16/8 h light/ dark photoperiod at 25 °C with ~ 200 μmol m^−2^ s^−1^ light intensity. The leaves of 2-month-old plants were harvested, frozen immediately in liquid nitrogen, and stored at -80 °C for DNA extraction, RNA-seq, and widely targeted metabolome analysis as described below.

### DNA extraction, sequencing, and phylogenetic analysis

Total genomic DNA was extracted from plant leaves using the TGuide plant Genomic DNA Extraction Kit (Tiangen, OSR-M301). ITS sequences were amplified using the primers ITS2F (5′-ATGCGATACTTGGTGTGAAT-3′) and ITS3R (5′-GACGCTTCTCCAGACTACAAT-3′). Sequencing was conducted by Sangon Biotech (Shanghai) Co., Ltd. Eleven ITS2 sequences of *Artemisia* and *Aster* species were downloaded from the GenBank. The ITS2 sequences of 13 *Artemisia* samples, namely NYYY, NYSY, *A. montana* (FJ643027.1), *A. stolonifera* (KU555697.1), *A. princeps* (KU855168.1), *A. argyi* (DQ925700.1), *A. feddei* (FJ643013.1), *A. frigida* (JN861917.1), *A. absinthium* (KX581838.1), *A. sieversiana* (KU855170.1), *A. scoparia* (KU555638.1), *A. capillaris* (KX675134.1), *A. fukudo* (IM150921.1), were aligned with that of *Aster spathulifolius* (HQ154050.1) using UPGMA method. Phylogenetic trees were constructed in MEGAX [60] with *Aster spathulifolius* was serving as the out-group due to its distinct separation from the in-group taxa.

### Metabolite extraction and ultra-performance liquid chromatography–tandem mass spectrometry (UPLC-MS/MS) analysis

Leaves were harvested from two-month-old greenhouse-grown plants. After collection, samples were freeze-dried in a vacuum freeze-dryer (Scientz-100F). Freeze-dried samples were crushed using a mixer mill (MM 400, Retsch) with a zirconia bead for 1.5 min at 30 Hz. Lyophilized powder (100 mg per sample) was dissolved in 1.2 ml 70% methanol solution and vortexed for 30 s every 30 min a total of six times. Samples were then incubated at 4 °C overnight. After centrifugation at 12,000 g for 10 min, extracts were filtrated (SCAA-104, 0.22 μm pore size; ANPEL, Shanghai, China, http://www.anpel.com.cn/) before UPLC-MS/MS analysis. UPLC-MS/MS analysis was performed as previously reported [61]. There were three biological replicates per cultivar. Quality control (QC) samples were prepared by mixing all six sample extracts.

### Flavonoids content analysis

Flavonoids were extracted from *Artemisia* samples according to the following protocol. Briefly, 100 mg of *Artemisia* leaf powder was extracted with 70% methanol (v/v) at 4 °C overnight and vortexed three times. Samples were centrifuged at 12,000 g for 10 min. The supernatant fractions were filtrated through a micropore filter membrane (0.22 μm pore size).

Flavonoids content was measured in these sample extract using previously described spectrophotometric methods with slight modifications. A total of 40 µL sample stock solution or rutin (0.2 mg mL^−1^) was mixed with 40 µL 5% NaNO_2_, then incubated at room temperature for 6 min. To this mixture, 40 µL 10% Al(NO3)_3_ was added and samples were incubated at room temperature for 6 min. Finally, 400 µL 4% NaOH was added to the above mixture, and samples were dilute with 70% methanol to a final volume 1 mL. After incubation at room temperature for 30 min, absorbance values were measured at 510 nm using a SpectraMax M_3_. A calibration curve (y = 1.4682X -0.0061, R^2^ = 0.9975) was generated from rutin at final concentrations of 0.08, 0.24, 0.32, 0.40, and 0.48 μg/mL. Flavonoids contents were expressed in milligramequivalents of rutin per gram of dry weight (mg /g). There were three biological replicates for each cultivar.

### Antioxidant capacity measurements

DPPH radical scavenging activities were measured to determine total antioxidant capacity as follows: 400 μL of DPPH solution in absolute ethanol and 20 μL of the sample supernatant were added to a 96-well microplate. The mixture was incubated at room temperature under the dark for 30 min, then absorbance values at 517 nm were measured using a SpectraMax M3. The DPPH radical scavenging activities of each sample were calculated as the percent inhibition according to the following equation: DPPH radical scavenging activity (%) = [1—(A1—A2) ÷ A3] × 100%. where A1 is the absorbance of the test sample, A2 is the absorbance of the positive control, and A3 is the absorbance of the blank.

The scavenging capacity for ABTS radical cationswas measured using a total antioxidant capacity assay kit (Suzhou Grace Biotechnology Co.,Ltd, G0127W) following the manufacturer’s instructions. Supernatant (10 μL) and peroxidase solution (190 μL) were added to a 96-well microplate. After mixing, the solution was left at room temperature under dim light for 6 min, then the absorbance values at 414 nm were measured with Spectra Max M3. The antioxidant capacities were calculated using the equation in the protocol.

FRAP scavenging capacities were determined using a total antioxidant capacity assay kit (Suzhou Grace Biotechnology Co.,Ltd, G0115W). Following the manufacturer’s instructions, 170 μL of FRAP solution, 25ul uL of distilled water and 5 μL of supernatant were added to the wells of a 96-well microplate. The mixture was left at room temperature under dim light for 10 min, then the absorbance values at 590 nm were measured with a Spectra Max M3. The antioxidant capacities were calculated according to the equation in the protocol.

### qRT-PCR analysis

To validate the reliability of transcriptome results, qRT-PCR tests were performed. cDNA synthesis and qRT-PCR were performed as previously reported. The primers used for qRT-PCR analysis are showed in Supplementary Table S2. All samples were analyzed in three biological replicates. Relative expression levels were calculated using the 2^−ΔΔCt^ method with *AaActin* serving as the internal control.

### RNA isolation and transcriptome sequencing

Leaves were harvested from two-month-old greenhouse-grown plants and RNA was isolated from the collected samples (Tiangen, DP441). A total of six samples (two cultivars with three biological replicates each) were analyzed. cDNA libraries were sequenced on the Illumina sequencing platform by Metware Biotechnology Co., Ltd. (Wuhan, China). The raw data were filtered using fastp to ensure quality and reliability. The clean reads from all six samples were spliced de novo using Trinity software (N50 = 1178). A total of 375,626 unigenes were assembled, then annotated using seven major databases: the National Center for Biotechnology Information (NCBI) non-redundant (NR) protein database, the Protein Family (Pfam) database, Swiss-Prot (a manually annotated and reviewed protein sequence database), the Kyoto Encyclopedia of Genes and Genomes (KEGG) database [27], Clusters of Orthologous Genes (COG)/EuKaryotic Orthologous Groups (KOG), and the Gene Ontology (GO) database. DEGs between the two cultivars were identified using DESeq2 v1.22.1 with thresholds of log2(|fold change|) ≥ 2 and *p* < 0.01.

### GSEA

Fold change values of all genes between NYSY and NYYY was calculated. Based on the resulting values, GSEA was conducted using the ‘clusterprofiler’ package in R (GSEA 3.0) [62, 63].

### Statistical analyses 

After unit-variance scaling of the metabolite contents data, unsupervised PCA was performed using the statistics function ‘prcomp’ in R. Differences in total flavonoids s and antioxidant activities between the two cultivars were analyzed with a one-way analysis of variance (ANOVA) and Fisher’s least significant difference (LSD) test at the 5% level in base R.

## Supplementary Information

**Additional file 1:** **Figure S1.** Correlation and PCA analysis of metabolomic data. (A) Correlation diagram based on metabolomic data. The square (R^2^) of Pearson correlation coefficients between biological replicates should be >0.8. (B) PCA analysis of metabolomic data. The x-axis represents the first principal component and the y-axis represents the second principal component. **Figure S2.** Vol map of significantly up regulated, down regulated, and insignificantly regulated metabolites between NYSY and NYYY.

**Additional file 2:** **Table S1.** Metabolites detected in NYSY and NYYY.

**Additional file 3: Table S2.** qRT-PCR primers used for validation different expressed genes between NYSY and NYYY.

## Data Availability

The RNA sequence data are in the NCBI’s Sequence Read Archive (SRA) under the accession number PRJNA821649. https://www.ncbi.nlm.nih.gov/bioproject/PRJNA821649.
