# Supplementary material for: Metabolomic and transcriptomic analyses provide insights into variations in flavonoids contents between two Artemisia cultivars
Source: BMC Plant Biol. 2023 May 30;23:288. doi: 10.1186/s12870-023-04295-8 (PMC10228080; doi:10.1186/s12870-023-04295-8)
Supplement: Supplementary file 1 — Additional file 1: Figure S1. Correlation and PCA analysis of metabolomic data. (A) Correlation diagram based on metabolomic data. The square (R2) of Pearson correlation coefficients between biological replicates should be >0.8. (B) PCA analysis of metabolomic data. The x-axis represents the first principal component and the y-axis represents the second principal component. Figure S2. Vol map of significantly up regulated, down regulated, and insignificantly regulated metabolites between NYSY and NYYY. [file 12870_2023_4295_MOESM1_ESM.pdf]

(A)

(B)

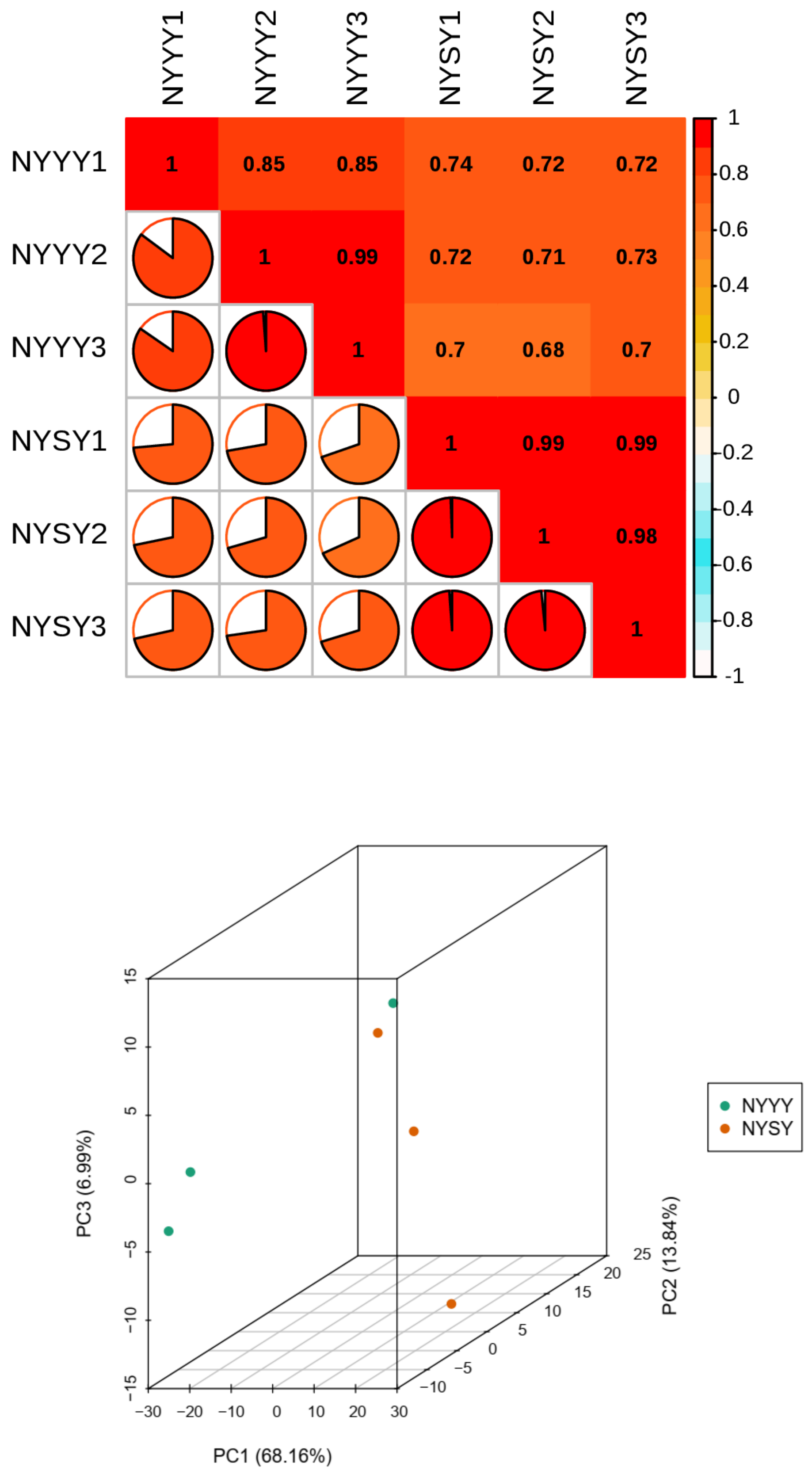

Figure S1. Correlation and PCA analysis of metabolomic data. (A) Correlation diagram based on metabolomic data. The square (R^2) of Pearson correlation coefficients between biological replicates should be >0.8. (B) PCA analysis of metabolomic data. The x-axis represents the first principal component and the y-axis represents the second principal component.

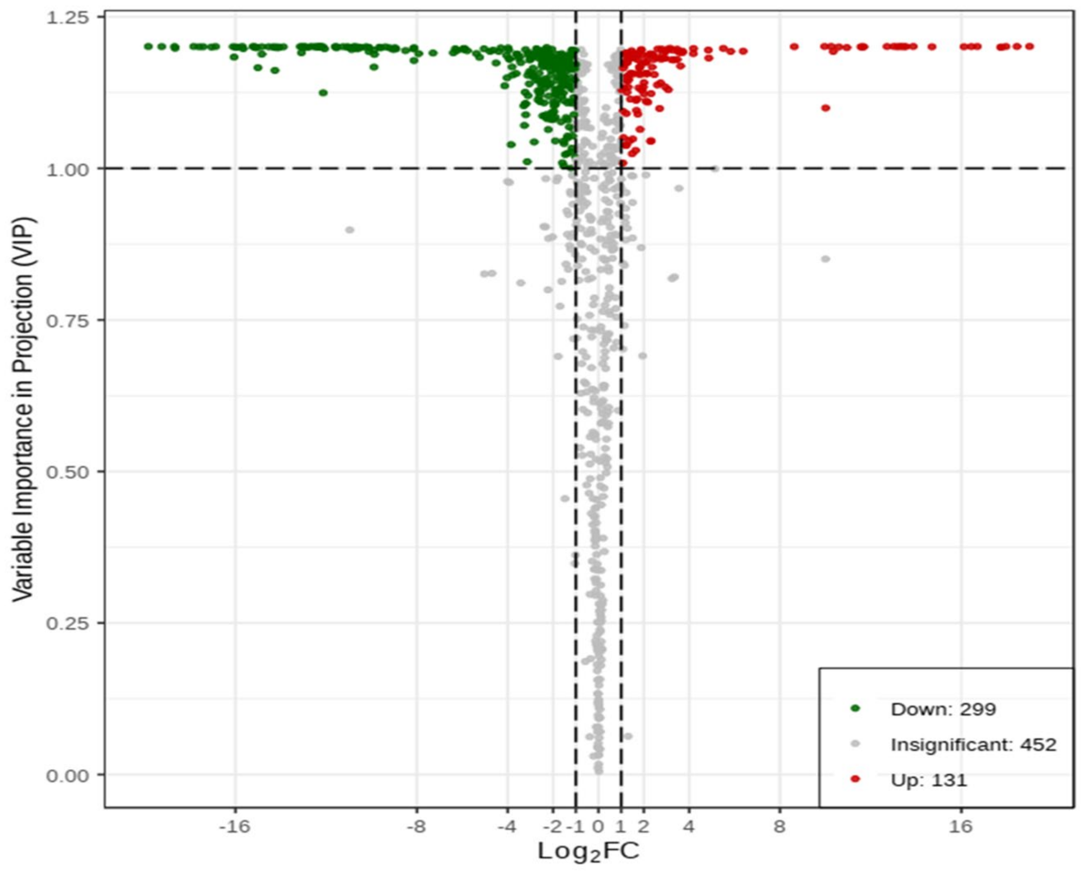

Figure S2. Vol map of significantly up regulated, down regulated, and insignificantly regulated metabolites between NYSY and NYYY.
