# Supplementary material for: Metabolomic and transcriptomic analyses provide insights into variations in flavonoids contents between two Artemisia cultivars
Source: BMC Plant Biol. 2023 May 30;23:288. doi: 10.1186/s12870-023-04295-8 (PMC10228080; doi:10.1186/s12870-023-04295-8)
Supplement: Supplementary file 2 — Additional file 2: Table S1. Metabolites detected in NYSY and NYYY. [file 12870_2023_4295_MOESM2_ESM.pdf]

Table S1. Metabolites detected in NYSY and NYYY

Compounds	Formula	Class I	Class II	NYYY1	NYYY2	NYYY3	NYSY1	NYSY2	NYSY3	VIP	Fold Change	Log2FC	Type
Indole	C8H7N	Alkaloids	Plumerane	6.78E+03	1.38E+04	7.94E+03	8.84E+03	1.07E+04	9.05E+03	1.24E-01	1.00E+00	-5.71E-04	insig
N-Benzylmethylene isomethylamine	C8H9N	Alkaloids	Alkaloids	1.14E+06	8.61E+05	8.33E+05	2.59E+06	2.47E+06	1.87E+06	1.15E+00	2.44E+00	1.29E+00	up
1-Methylhistamine	C6H11N3	Alkaloids	Alkaloids	1.13E+04	1.18E+04	1.14E+04	1.02E+04	1.32E+04	1.10E+04	7.87E-02	9.98E-01	-3.55E-03	insig
6-Deoxyfagomine	C6H13NO2	Alkaloids	Alkaloids	8.35E+05	7.98E+05	6.98E+05	9.09E+05	9.05E+05	7.54E+05	6.14E-01	1.10E+00	1.39E-01	insig
Stachydrine	C7H13NO2	Alkaloids	Pyrrole alkaloids	1.58E+06	1.76E+06	2.25E+06	4.45E+05	1.87E+05	3.69E+05	1.14E+00	1.79E-01	-2.48E+00	down
Nicotine	C10H14N2	Alkaloids	Alkaloids	7.47E+05	6.58E+05	2.59E+05	2.57E+05	3.57E+05	2.51E+05	7.52E-01	5.19E-01	-9.46E-01	insig
Candicine	C11H18NO+	Alkaloids	Alkaloids	1.45E+05	1.93E+05	1.28E+05	1.79E+05	2.31E+05	2.69E+05	8.90E-01	1.46E+00	5.43E-01	insig
4-(Rhamnosyloxy)phenylacetonitrile	C14H17NO5	Alkaloids	Alkaloids	6.54E+04	8.77E+04	7.71E+04	9.00E+00	9.00E+00	9.00E+00	1.20E+00	1.17E-04	-1.31E+01	down
2-(D-Glucosyloxy)-4-hydroxybenzeneacetonitrile	C14H17NO7	Alkaloids	Alkaloids	6.01E+03	9.32E+03	6.09E+03	3.76E+04	3.43E+04	2.25E+04	1.16E+00	4.40E+00	2.14E+00	up
5,6-Dihydroxyindole-5-O-β-glucoside	C14H17NO7	Alkaloids	Alkaloids	2.74E+04	1.28E+04	1.84E+04	4.41E+04	3.83E+04	4.35E+04	1.05E+00	2.15E+00	1.10E+00	up
10-Formyltetrahydrofuran	C20H23N7O7	Alkaloids	Alkaloids	1.08E+05	7.17E+04	5.87E+04	1.43E+05	1.37E+05	1.36E+05	1.03E+00	1.75E+00	8.07E-01	insig
Bis(N,N-Diethylethanaminium)-2-acetamido-1,5-anhydro-2-deoxy-1-[-hydroxy(phosphonato)methyl]-D-glucitol	C21H48N3O9P	Alkaloids	Alkaloids	3.88E+05	2.95E+05	2.69E+05	2.15E+05	3.27E+05	3.11E+05	3.87E-01	8.96E-01	-1.58E-01	insig
Betanin (Betanidin-5-O-glucoside)	C24H26N2O13	Alkaloids	Alkaloids	2.02E+04	1.23E+05	1.17E+05	1.17E+04	6.86E+03	1.10E+04	1.01E+00	1.13E-01	-3.14E+00	down
3-Hydroxypropyl palmitate glc-glucosamine	C31H61O14N	Alkaloids	Alkaloids	1.02E+06	9.09E+05	8.29E+05	7.92E+05	1.23E+06	8.57E+05	9.37E-02	1.04E+00	5.90E-02	insig
N,N-Dimethylglycine	C4H9NO2	Amino acids and derivatives	Amino acids and derivatives	6.58E+05	4.49E+05	4.95E+05	4.17E+05	3.58E+05	3.51E+05	9.61E-01	7.02E-01	-5.09E-01	insig
2-Aminoisobutyric acid	C4H9NO2	Amino acids and derivatives	Amino acids and derivatives	3.21E+06	2.87E+06	3.45E+06	2.57E+06	2.13E+06	2.88E+06	8.99E-01	7.96E-01	-3.29E-01	insig
L-Serine	C3H7NO3	Amino acids and derivatives	Amino acids and derivatives	1.65E+05	1.24E+05	1.77E+05	1.93E+05	1.94E+05	1.95E+05	8.73E-01	1.25E+00	3.24E-01	insig
Histamine	C5H9N3	Amino acids and derivatives	Amino acids and derivatives	1.23E+04	6.20E+03	2.62E+03	6.64E+03	1.74E+04	2.04E+04	7.02E-01	2.10E+00	1.07E+00	insig
L-Proline	C5H9NO2	Amino acids and derivatives	Amino acids and derivatives	5.20E+06	3.48E+06	4.83E+06	2.01E+07	2.00E+07	1.56E+07	1.17E+00	4.12E+00	2.04E+00	up
5-Aminovaleric acid	C5H11NO2	Amino acids and derivatives	Amino acids and derivatives	6.65E+05	3.59E+05	5.98E+05	1.87E+04	1.81E+04	2.23E+04	1.19E+00	3.64E-02	-4.78E+00	down
L-Valine	C5H11NO2	Amino acids and derivatives	Amino acids and derivatives	1.73E+07	1.60E+07	1.39E+07	2.13E+07	2.19E+07	1.97E+07	1.08E+00	1.33E+00	4.14E-01	insig
L-Threonine	C4H9NO3	Amino acids and derivatives	Amino acids and derivatives	2.19E+05	1.10E+05	1.51E+05	3.05E+05	4.20E+05	2.24E+05	9.41E-01	1.98E+00	9.85E-01	insig
L-Cysteine	C3H7NO2S	Amino acids and derivatives	Amino acids and derivatives	1.15E+04	1.95E+04	1.85E+04	1.85E+04	2.30E+04	1.24E+04	1.90E-01	1.09E+00	1.22E-01	insig
5-Oxo-L-Proline	C5H7NO3	Amino acids and derivatives	Amino acids and derivatives	2.43E+04	9.84E+03	2.16E+04	3.48E+04	2.77E+04	6.59E+04	8.82E-01	2.31E+00	1.21E+00	insig
Pipecolic acid	C6H11NO2	Amino acids and derivatives	Amino acids and derivatives	8.15E+06	9.47E+06	1.40E+07	2.75E+06	1.88E+06	2.79E+06	1.15E+00	2.35E-01	-2.09E+00	down
Trans-4-Hydroxy-L-proline	C5H9NO3	Amino acids and derivatives	Amino acids and derivatives	1.36E+05	1.44E+05	1.46E+05	1.53E+05	1.49E+05	1.40E+05	6.12E-01	1.04E+00	5.69E-02	insig
N-Propionylglycine	C5H9NO3	Amino acids and derivatives	Amino acids and derivatives	2.58E+04	2.54E+04	3.39E+04	2.38E+04	3.12E+04	3.09E+04	3.25E-02	1.01E+00	1.38E-02	insig
L-Leucine	C6H13NO2	Amino acids and derivatives	Amino acids and derivatives	1.61E+07	1.60E+07	1.58E+07	1.93E+07	2.01E+07	1.64E+07	9.17E-01	1.16E+00	2.20E-01	insig
L-Isoleucine	C6H13NO2	Amino acids and derivatives	Amino acids and derivatives	1.66E+07	1.64E+07	1.59E+07	1.78E+07	1.89E+07	1.68E+07	9.11E-01	1.09E+00	1.27E-01	insig
L-Norleucine	C6H13NO2	Amino acids and derivatives	Amino acids and derivatives	1.49E+07	1.63E+07	1.41E+07	1.59E+07	1.69E+07	1.49E+07	5.17E-01	1.05E+00	7.44E-02	insig
L-Asparagine	C4H8N2O3	Amino acids and derivatives	Amino acids and derivatives	7.82E+04	7.71E+04	6.25E+04	5.96E+04	7.50E+04	5.51E+04	6.22E-01	8.71E-01	-1.99E-01	insig
L-Ornithine	C5H12N2O2	Amino acids and derivatives	Amino acids and derivatives	8.42E+03	1.00E+04	8.13E+03	9.08E+03	9.20E+03	6.67E+03	3.22E-01	9.38E-01	-9.25E-02	insig
L-Aspartic Acid	C4H7NO4	Amino acids and derivatives	Amino acids and derivatives	2.15E+06	1.56E+06	2.09E+06	5.39E+06	4.98E+06	4.05E+06	1.15E+00	2.49E+00	1.31E+00	up
L-Tyramine	C8H11NO	Amino acids and derivatives	Amino acids and derivatives	8.48E+05	1.20E+06	5.22E+05	3.05E+06	3.15E+06	3.93E+06	1.13E+00	3.93E+00	1.98E+00	up
1-Methylpiperidine-2-carboxylic acid	C7H13NO2	Amino acids and derivatives	Amino acids and derivatives	2.24E+06	1.83E+06	2.96E+06	1.26E+05	8.88E+04	1.21E+05	1.19E+00	4.77E-02	-4.39E+00	down
D-Proline betaine	C7H14NO2+	Amino acids and derivatives	Amino acids and derivatives	1.03E+06	8.80E+05	1.28E+06	3.48E+04	3.61E+04	2.57E+04	1.20E+00	3.03E-02	-5.05E+00	down
L-Glutamine	C5H10N2O3	Amino acids and derivatives	Amino acids and derivatives	1.11E+06	9.96E+05	1.11E+06	1.17E+06	1.26E+06	1.19E+06	9.68E-01	1.13E+00	1.71E-01	insig
L-Lysine	C6H14N2O2	Amino acids and derivatives	Amino acids and derivatives	1.00E+06	9.26E+05	1.02E+06	1.11E+06	1.15E+06	1.09E+06	1.06E+00	1.14E+00	1.84E-01	insig
L-threo-3-Methylaspartate	C5H9NO4	Amino acids and derivatives	Amino acids and derivatives	7.82E+05	4.60E+05	5.93E+05	1.17E+06	1.13E+06	1.05E+06	1.07E+00	1.83E+00	8.69E-01	insig
O-Acetylserine	C5H9NO4	Amino acids and derivatives	Amino acids and derivatives	1.80E+05	2.13E+05	1.68E+05	3.65E+05	4.04E+05	3.57E+05	1.17E+00	2.01E+00	1.01E+00	up
L-Glutamic acid	C5H9NO4	Amino acids and derivatives	Amino acids and derivatives	7.16E+06	3.16E+06	4.34E+06	1.11E+07	1.34E+07	9.65E+06	1.04E+00	2.32E+00	1.22E+00	up
L-Methionine	C5H11NO2S	Amino acids and derivatives	Amino acids and derivatives	4.33E+05	3.81E+05	4.62E+05	5.79E+05	5.58E+05	4.39E+05	8.48E-01	1.24E+00	3.05E-01	insig
L-Histidine	C6H9N3O2	Amino acids and derivatives	Amino acids and derivatives	8.90E+04	8.48E+04	9.33E+04	1.25E+05	7.13E+04	9.09E+04	1.80E-01	1.07E+00	1.02E-01	insig
S-Allyl-L-cysteine	C6H11NO2S	Amino acids and derivatives	Amino acids and derivatives	5.99E+03	4.29E+03	4.73E+03	5.05E+03	6.42E+03	4.78E+03	3.37E-01	1.08E+00	1.14E-01	insig
N-Acetyl-L-threonine	C6H11NO4	Amino acids and derivatives	Amino acids and derivatives	5.19E+04	3.19E+04	5.32E+04	7.64E+04	5.43E+04	6.06E+04	8.03E-01	1.40E+00	4.83E-01	insig
3-Hydroxy-3-methylpentane-1,5-dioic acid	C6H10O5	Amino acids and derivatives	Amino acids and derivatives	3.56E+06	3.12E+06	3.32E+06	1.71E+06	1.36E+06	1.66E+06	1.17E+00	4.74E-01	-1.08E+00	down
L-Methionine methyl ester	C6H13NO2S	Amino acids and derivatives	Amino acids and derivatives	2.20E+04	2.10E+04	2.44E+04	2.50E+04	2.54E+04	2.20E+04	5.79E-01	1.07E+00	1.02E-01	insig
L-Methionine Sulfoxide	C5H11NO3S	Amino acids and derivatives	Amino acids and derivatives	4.53E+06	4.58E+06	4.26E+06	5.46E+06	4.84E+06	5.77E+06	1.03E+00	1.20E+00	2.65E-01	insig
L-Phenylalanine	C9H11NO2	Amino acids and derivatives	Amino acids and derivatives	2.95E+06	2.16E+06	1.97E+06	6.54E+06	6.32E+06	4.69E+06	1.13E+00	2.48E+00	1.31E+00	up
3-Methyl-L-Histidine	C7H11N3O2	Amino acids and derivatives	Amino acids and derivatives	2.64E+04	2.63E+04	2.42E+04	1.97E+04	2.16E+04	2.80E+04	5.61E-01	9.01E-01	-1.50E-01	insig
N-Acetyl-L-leucine	C8H15NO3	Amino acids and derivatives	Amino acids and derivatives	2.02E+04	1.01E+04	1.32E+04	2.53E+04	1.81E+04	1.81E+04	7.59E-01	1.41E+00	4.98E-01	insig
L-Theanine	C7H14N2O3	Amino acids and derivatives	Amino acids and derivatives	2.80E+04	1.63E+04	2.61E+04	4.11E+03	7.19E+03	7.71E+03	1.12E+00	2.70E-01	-1.89E+00	down
N-α-Acetyl-L-ornithine	C7H14N2O3	Amino acids and derivatives	Amino acids and derivatives	3.03E+04	2.27E+04	2.53E+04	2.94E+04	2.31E+04	2.94E+04	2.39E-01	1.05E+00	6.52E-02	insig
L-Arginine	C6H14N4O2	Amino acids and derivatives	Amino acids and derivatives	4.46E+05	2.47E+05	2.80E+05	1.23E+06	4.04E+05	7.20E+05	9.01E-01	2.42E+00	1.27E+00	insig
N-Acetyl-L-Aspartic Acid	C6H9NO5	Amino acids and derivatives	Amino acids and derivatives	1.15E+05	7.10E+04	9.40E+04	2.25E+05	2.31E+05	2.17E+05	1.14E+00	2.41E+00	1.27E+00	up
L-Citrulline	C6H13N3O3	Amino acids and derivatives	Amino acids and derivatives	6.14E+04	3.52E+04	3.75E+04	4.84E+04	4.15E+04	5.02E+04	2.35E-01	1.04E+00	6.29E-02	insig
L-Tyrosine	C9H11NO3	Amino acids and derivatives	Amino acids and derivatives	6.72E+06	6.25E+06	6.39E+06	1.11E+07	1.11E+07	1.00E+07	1.19E+00	1.67E+00	7.37E-01	insig
N-Acetyl-L-Glutamine	C7H12N2O4	Amino acids and derivatives	Amino acids and derivatives	7.27E+05	6.75E+05	7.85E+05	9.25E+05	7.94E+05	9.96E+05	9.64E-01	1.24E+00	3.11E-01	insig
N-Glycyl-L-leucine	C8H16N2O3	Amino acids and derivatives	Amino acids and derivatives	1.02E+05	1.17E+05	1.19E+05	1.09E+05	9.10E+04	9.73E+04	7.86E-01	8.81E-01	-1.83E-01	insig
L-Glycyl-L-isoleucine	C8H16N2O3	Amino acids and derivatives	Amino acids and derivatives	7.88E+04	9.89E+04	9.37E+04	8.77E+04	8.43E+04	7.97E+04	5.20E-01	9.28E-01	-1.08E-01	insig
N6-Acetyl-L-lysine	C8H16N2O3	Amino acids and derivatives	Amino acids and derivatives	2.03E+05	1.78E+05	1.32E+05	2.68E+05	2.53E+05	2.59E+05	1.04E+00	1.52E+00	6.03E-01	insig
Homoarginine	C7H16N4O2	Amino acids and derivatives	Amino acids and derivatives	2.71E+03	2.10E+03	2.45E+03	1.01E+03	2.00E+03	1.15E+03	9.73E-01	5.73E-01	-8.04E-01	insig
N-Monomethyl-L-arginine	C7H16N4O2	Amino acids and derivatives	Amino acids and derivatives	9.03E+04	7.05E+04	7.46E+04	2.07E+05	8.16E+04	1.12E+05	7.69E-01	1.70E+00	7.67E-01	insig
N-Acetyl-L-glutamic acid	C7H11NO5	Amino acids and derivatives	Amino acids and derivatives	2.94E+04	7.15E+03	5.63E+03	8.03E+04	7.84E+04	5.24E+04	1.05E+00	5.00E+00	2.32E+00	up
L-Homocitrulline	C7H15N3O3	Amino acids and derivatives	Amino acids and derivatives	9.31E+04	6.06E+04	4.28E+04	8.46E+04	9.27E+04	6.47E+04	5.38E-01	1.23E+00	3.00E-01	insig
2,6-Diaminooimelic acid	C7H14N2O4	Amino acids and derivatives	Amino acids and derivatives	8.99E+03	7.06E+03	9.00E+03	1.12E+04	1.21E+04	7.90E+03	6.70E-01	1.24E+00	3.15E-01	insig
L-Dopa	C9H11NO4	Amino acids and derivatives	Amino acids and derivatives	4.22E+05	2.57E+05	2.59E+05	1.87E+05	1.49E+05	1.65E+05	1.03E+00	5.35E-01	-9.02E-01	insig
L-Alanyl-L-leucine	C9H18N2O3	Amino acids and derivatives	Amino acids and derivatives	1.68E+04	1.51E+04	1.58E+04	1.57E+04	1.82E+04	1.32E+04	1.33E-01	9.87E-01	-1.87E-02	insig
NG,NG-Dimethyl-L-arginine	C8H18N4O2	Amino acids and derivatives	Amino acids and derivatives	5.80E+04	6.52E+04	5.49E+04	8.30E+04	6.38E+04	5.76E+04	5.80E-01	1.15E+00	1.99E-01	insig
L-Tryptophan	C11H12N2O2	Amino acids and derivatives	Amino acids and derivatives	1.76E+06	2.92E+06	2.72E+06	3.94E+06	3.02E+06	3.22E+06	8.30E-01	1.37E+00	4.58E-01	insig
N-Acetyl-L-phenylalanine	C11H13NO3	Amino acids and derivatives	Amino acids and derivatives	1.57E+05	2.94E+05	2.72E+05	3.45E+05	2.89E+05	3.16E+05	7.25E-01	1.31E+00	3.93E-01	insig

3-(2-Naphthyl)-L-alanine	C13H13NO2	Amino acids and derivatives	Amino acids and derivatives	3.55E+03	9.00E+00	3.58E+02	4.01E+04	3.76E+04	5.91E+04	9.99E-01	3.50E+01	5.13E+00	insig
N-Acetyl-L-Arginine	C8H16N4O3	Amino acids and derivatives	Amino acids and derivatives	1.94E+04	2.89E+04	2.80E+04	7.14E+04	4.92E+04	6.18E+04	1.13E+00	2.39E+00	1.26E+00	up
5-Hydroxy-L-tryptophan	C11H12N2O3	Amino acids and derivatives	Amino acids and derivatives	1.78E+04	2.51E+04	1.74E+04	2.55E+04	2.80E+04	2.55E+04	9.11E-01	1.31E+00	3.87E-01	insig
L-Glycyl-L-phenylalanine	C11H14N2O3	Amino acids and derivatives	Amino acids and derivatives	2.83E+04	2.93E+04	2.69E+04	3.96E+04	4.58E+04	4.08E+04	1.16E+00	1.49E+00	5.79E-01	insig
N-Acetyl-L-tyrosine	C11H13NO4	Amino acids and derivatives	Amino acids and derivatives	1.90E+04	2.23E+04	1.73E+04	1.74E+04	2.04E+04	1.67E+04	4.15E-01	9.29E-01	-1.06E-01	insig
L-Alanyl-L-Phenylalanine	C12H16N2O3	Amino acids and derivatives	Amino acids and derivatives	1.19E+04	1.36E+04	1.27E+04	6.97E+03	9.17E+03	7.73E+03	1.13E+00	6.24E-01	-6.81E-01	insig
L-Cystine	C6H12N2O4S2	Amino acids and derivatives	Amino acids and derivatives	2.03E+04	1.25E+04	1.51E+04	1.39E+04	1.18E+04	1.08E+04	7.58E-01	7.61E-01	-3.94E-01	insig
N-(3-Indolylacetyl)-L-alanine	C13H14N2O3	Amino acids and derivatives	Amino acids and derivatives	1.80E+04	4.50E+04	5.34E+04	9.00E+00	9.00E+00	9.00E+00	1.20E+00	2.32E-04	-1.21E+01	down
N-Acetyl-L-Tryptophan	C13H14N2O3	Amino acids and derivatives	Amino acids and derivatives	1.64E+04	8.52E+03	5.94E+03	3.57E+04	2.97E+04	3.70E+04	1.09E+00	3.33E+00	1.73E+00	up
L-Saccharopine	C11H20N2O6	Amino acids and derivatives	Amino acids and derivatives	3.31E+03	4.69E+03	4.04E+03	1.93E+03	2.63E+03	1.56E+03	1.07E+00	5.08E-01	-9.76E-01	insig
L-Leucyl-L-phenylalanine	C15H22N2O3	Amino acids and derivatives	Amino acids and derivatives	4.16E+04	4.07E+04	3.91E+04	1.99E+04	1.64E+04	1.16E+04	1.14E+00	3.95E-01	-1.34E+00	down
L-Aspartyl-L-Phenylalanine	C13H16N2O5	Amino acids and derivatives	Amino acids and derivatives	6.92E+03	5.07E+03	8.27E+03	1.90E+04	2.14E+04	1.92E+04	1.16E+00	2.94E+00	1.56E+00	up
Nicotianamine	C12H21N3O6	Amino acids and derivatives	Amino acids and derivatives	3.95E+03	3.67E+03	5.61E+03	7.04E+03	3.89E+03	5.46E+03	5.15E-01	1.24E+00	3.08E-01	insig
L-Glutamine-O-glycoside	C11H20N2O8	Amino acids and derivatives	Amino acids and derivatives	1.47E+03	5.25E+03	5.27E+03	5.47E+03	4.19E+03	6.04E+03	5.08E-01	1.31E+00	3.90E-01	insig
L-Glutamic acid-O-glycoside	C11H19NO9	Amino acids and derivatives	Amino acids and derivatives	1.25E+03	1.80E+03	2.57E+03	8.17E+03	8.01E+03	8.26E+03	1.16E+00	4.35E+00	2.12E+00	up
L-Phenylalanyl-L-phenylalanine	C18H20N2O3	Amino acids and derivatives	Amino acids and derivatives	1.23E+04	4.12E+05	4.68E+05	9.00E+00	9.00E+00	9.00E+00	1.17E+00	3.02E-05	-1.50E+01	down
S-(Methyl)glutathione	C11H19N3O6S	Amino acids and derivatives	Amino acids and derivatives	5.51E+03	8.52E+03	1.93E+04	3.68E+04	2.24E+04	3.64E+04	9.87E-01	2.87E+00	1.52E+00	insig
S-(5'-Adenosy)-L-homocysteine	C14H20N6O5S	Amino acids and derivatives	Amino acids and derivatives	6.20E+04	6.20E+04	5.47E+04	1.38E+05	1.30E+05	8.26E+04	1.07E+00	1.96E+00	9.73E-01	insig
S-Adenosyl-L-methionine	C15H22N6O5S	Amino acids and derivatives	Amino acids and derivatives	1.30E+05	9.51E+04	1.09E+05	1.85E+05	2.08E+05	1.95E+05	1.14E+00	1.76E+00	8.18E-01	insig
L-Aspartic acid-O-diglucoside	C16H27NO14	Amino acids and derivatives	Amino acids and derivatives	9.93E+04	9.65E+04	9.69E+04	1.84E+05	2.08E+05	1.32E+05	1.08E+00	1.79E+00	8.38E-01	insig
Astin J	C25H33N5O7	Amino acids and derivatives	Amino acids and derivatives	7.37E+03	1.53E+04	9.14E+03	3.14E+04	4.23E+04	4.53E+04	1.13E+00	3.73E+00	1.90E+00	up
Astin H	C25H32ClN5O7	Amino acids and derivatives	Amino acids and derivatives	4.01E+05	3.70E+05	4.64E+05	2.52E+05	2.99E+05	3.16E+05	1.07E+00	7.02E-01	-5.11E-01	insig
Oxiglutatione	C20H32N6O12S2	Amino acids and derivatives	Amino acids and derivatives	8.53E+03	7.69E+03	8.24E+03	1.56E+04	8.17E+03	1.38E+04	8.52E-01	1.54E+00	6.20E-01	insig
3',4',7-Trihydroxyflavone	C15H10O5	Flavonoids	Flavonoid	9.59E+03	1.37E+04	7.39E+03	2.89E+03	2.14E+03	1.59E+03	1.14E+00	2.15E-01	-2.21E+00	down
Apigenin	C15H10O5	Flavonoids	Flavonoid	1.69E+05	2.59E+05	2.44E+05	5.94E+04	4.33E+04	4.70E+04	1.17E+00	2.23E-01	-2.17E+00	down
Naringenin	C15H12O5	Flavonoids	Dihydroflavone	2.40E+06	3.47E+06	3.78E+06	6.45E+05	6.28E+05	7.41E+05	1.18E+00	2.09E-01	-2.26E+00	down
Pinobanksin	C15H12O5	Flavonoids	Dihydroflavonol	2.41E+06	3.45E+06	3.69E+06	6.78E+05	6.64E+05	7.41E+05	1.18E+00	2.18E-01	-2.20E+00	down
Naringenin chalcone	C15H12O5	Flavonoids	Chalcones	1.66E+06	2.73E+06	2.73E+06	4.19E+05	4.05E+05	4.57E+05	1.18E+00	1.80E-01	-2.48E+00	down
Izalpinin	C16H12O5	Flavonoids	Flavonols	2.14E+05	1.71E+05	6.35E+04	1.91E+04	1.46E+04	1.83E+04	1.12E+00	1.16E-01	-3.11E+00	down
Prunetin	C16H12O5	Flavonoids	Isoflavones	3.78E+06	3.12E+06	1.00E+06	3.05E+05	2.33E+05	3.17E+05	1.11E+00	1.08E-01	-3.21E+00	down
Acacetin	C16H12O5	Flavonoids	Flavonoid	5.63E+05	5.21E+05	1.57E+05	4.49E+04	3.49E+04	5.29E+04	1.11E+00	1.07E-01	-3.22E+00	down
Isoscutellarein	C15H10O6	Flavonoids	Flavonoid	2.45E+05	3.57E+05	3.25E+05	1.56E+05	1.19E+05	1.42E+05	1.13E+00	4.50E-01	-1.15E+00	down
2'-Hydroxygenistein	C15H10O6	Flavonoids	Isoflavones	2.32E+05	3.56E+05	3.02E+05	1.59E+05	1.11E+05	1.29E+05	1.11E+00	4.48E-01	-1.16E+00	down
Luteolin	C15H10O6	Flavonoids	Flavonoid	7.53E+05	1.02E+06	8.69E+05	4.57E+05	3.10E+05	3.69E+05	1.13E+00	4.30E-01	-1.22E+00	down
Maritimetin	C15H10O6	Flavonoids	Sinensetin	2.22E+03	6.66E+03	7.28E+03	1.66E+03	1.58E+03	3.18E+03	8.34E-01	3.97E-01	-1.33E+00	insig
Kaempferol	C15H10O6	Flavonoids	Flavonols	3.54E+04	8.03E+04	9.57E+04	3.98E+04	3.32E+04	7.46E+04	4.78E-01	6.98E-01	-5.19E-01	insig
Eriodictyol	C15H12O6	Flavonoids	Dihydroflavone	1.23E+07	1.87E+07	1.95E+07	2.37E+06	2.51E+06	3.10E+06	1.18E+00	1.58E-01	-2.66E+00	down
Isookanin	C15H12O6	Flavonoids	Dihydroflavone	2.26E+04	3.57E+04	3.05E+04	7.88E+03	7.77E+03	9.51E+03	1.17E+00	2.83E-01	-1.82E+00	down
Okanin	C15H12O6	Flavonoids	Chalcones	4.02E+06	5.88E+06	6.07E+06	6.62E+05	6.62E+05	8.72E+05	1.19E+00	1.37E-01	-2.86E+00	down
5,7,2'-Trhiyroxy-8-methoxyflavone	C16H12O6	Flavonoids	Flavonoid	2.39E+07	2.96E+07	2.99E+07	9.60E+06	7.69E+06	9.69E+06	1.18E+00	3.24E-01	-1.63E+00	down
Hispidulin	C16H12O6	Flavonoids	Flavonoid	5.03E+07	5.76E+07	5.70E+07	1.95E+07	1.59E+07	1.82E+07	1.19E+00	3.25E-01	-1.62E+00	down
Gnetifolin B	C16H12O6	Flavonoids	Flavonoid	2.30E+07	3.07E+07	3.05E+07	9.76E+06	8.16E+06	9.85E+06	1.18E+00	3.30E-01	-1.60E+00	down
Isoetin	C15H10O7	Flavonoids	Flavonoid	2.91E+04	2.12E+04	2.26E+04	1.33E+04	1.40E+04	1.21E+04	1.13E+00	5.41E-01	-8.86E-01	insig
Quercetin	C15H10O7	Flavonoids	Flavonols	8.37E+04	8.75E+04	7.36E+04	9.00E+00	9.00E+00	9.00E+00	1.20E+00	1.10E-04	-1.31E+01	down
Blumeatin	C16H14O6	Flavonoids	Flavonoid	2.33E+06	1.59E+06	1.46E+06	9.00E+00	9.00E+00	9.00E+00	1.20E+00	5.01E-06	-1.76E+01	down
Pectolinarigenin	C17H14O6	Flavonoids	Flavonoid	8.63E+06	1.50E+07	1.59E+07	3.35E+04	1.88E+04	2.22E+04	1.20E+00	1.88E-03	-9.05E+00	down
Dihydroxy-dimethoxyflavone	C17H14O6	Flavonoids	Flavonoid	8.63E+06	1.47E+07	1.61E+07	3.49E+04	2.43E+04	2.16E+04	1.20E+00	2.05E-03	-8.93E+00	down
Kumatakenin	C17H14O6	Flavonoids	Flavonols	9.77E+06	1.12E+07	1.21E+07	5.98E+05	4.95E+05	5.79E+05	1.20E+00	5.06E-02	-4.31E+00	down
4',5-Dihydroxy-3',5'-dimethoxyflavone	C17H14O6	Flavonoids	Flavonoid	8.69E+04	1.18E+05	1.27E+05	3.52E+04	2.41E+04	2.78E+04	1.17E+00	2.63E-01	-1.93E+00	down
Cirsimaritin	C17H14O6	Flavonoids	Flavonoid	1.72E+05	2.74E+05	3.07E+05	3.23E+03	3.14E+03	3.11E+03	1.20E+00	1.26E-02	-6.31E+00	down
Tamarixetin	C16H12O7	Flavonoids	Flavonoid	5.11E+03	5.67E+03	5.01E+03	1.40E+04	1.79E+04	2.29E+04	1.17E+00	3.47E+00	1.80E+00	up
Azaleatin	C16H12O7	Flavonoids	Flavonols	3.67E+04	5.20E+04	4.08E+04	4.04E+04	2.72E+04	3.68E+04	6.71E-01	8.06E-01	-3.12E-01	insig
Pedalitin	C16H12O7	Flavonoids	Flavonoid	4.15E+06	7.04E+06	7.39E+06	1.05E+05	5.31E+04	6.23E+04	1.19E+00	1.18E-02	-6.40E+00	down
Isorhamnetin	C16H12O7	Flavonoids	Flavonols	2.20E+04	3.11E+04	3.52E+04	1.29E+05	1.30E+05	1.95E+05	1.17E+00	5.13E+00	2.36E+00	up
Nepetin	C16H12O7	Flavonoids	Flavonoid	3.14E+07	3.50E+07	3.11E+07	1.37E+07	1.00E+07	1.15E+07	1.18E+00	3.62E-01	-1.47E+00	down
Ombuin	C17H14O7	Flavonoids	Flavonols	4.14E+05	2.51E+05	1.43E+05	3.81E+06	2.77E+06	3.31E+06	1.17E+00	1.23E+01	3.62E+00	up
Iristectorigenin B	C17H14O7	Flavonoids	Isoflavones	1.91E+07	3.00E+07	2.88E+07	9.63E+06	7.66E+06	9.79E+06	1.14E+00	3.48E-01	-1.52E+00	down
Rehderianin I	C17H14O7	Flavonoids	Flavonoid	1.49E+07	2.27E+07	2.19E+07	6.74E+06	5.42E+06	6.49E+06	1.16E+00	3.14E-01	-1.67E+00	down
Jaceosidin	C17H14O7	Flavonoids	Flavonoid	1.45E+07	2.27E+07	2.21E+07	6.67E+06	5.37E+06	6.83E+06	1.15E+00	3.18E-01	-1.65E+00	down
Quercetagetin-4'-Methyl Ether	C16H12O8	Flavonoids	Flavonoid	3.82E+03	2.98E+04	2.37E+04	5.05E+03	1.79E+03	5.42E+03	8.00E-01	2.14E-01	-2.22E+00	insig
Quercetagetin-3-Methyl Ether	C16H12O8	Flavonoids	Flavonoid	6.02E+03	3.42E+04	3.33E+04	6.82E+03	5.12E+03	4.02E+03	8.84E-01	2.17E-01	-2.20E+00	insig
Eupatorin	C18H16O7	Flavonoids	Flavonoid	1.35E+06	1.59E+06	1.76E+06	6.84E+05	6.23E+05	7.49E+05	1.17E+00	4.37E-01	-1.19E+00	down
Cirsilineol	C18H16O7	Flavonoids	Flavonoid	1.34E+07	1.54E+07	1.68E+07	6.54E+06	6.13E+06	7.25E+06	1.18E+00	4.37E-01	-1.20E+00	down
Pachypodol	C18H16O7	Flavonoids	Flavonoid	3.62E+05	8.16E+05	5.71E+05	9.00E+00	9.00E+00	9.00E+00	1.20E+00	1.54E-05	-1.60E+01	down
Eupatilin	C18H16O7	Flavonoids	Flavonoid	1.36E+06	1.61E+06	1.76E+06	6.92E+05	6.38E+05	7.52E+05	1.17E+00	4.40E-01	-1.18E+00	down
Ayanin	C18H16O7	Flavonoids	Flavonoid	2.77E+05	3.68E+05	3.96E+05	9.00E+00	9.00E+00	9.00E+00	1.20E+00	2.59E-05	-1.52E+01	down
5,6,7,4'-Tetramethoxyflavanone	C19H20O6	Flavonoids	Flavonoid	3.53E+06	4.35E+06	4.12E+06	6.04E+05	5.66E+05	6.31E+05	1.20E+00	1.50E-01	-2.74E+00	down
5,7,2',5'-Tetrahydroxy-8,6'-dimethoxyflavone	C17H14O8	Flavonoids	Flavonoid	3.28E+06	5.51E+06	4.97E+06	1.02E+05	6.72E+04	8.05E+04	1.19E+00	1.82E-02	-5.78E+00	down
Syringetin	C17H14O8	Flavonoids	Flavonoid	2.11E+06	3.58E+06	4.53E+06	5.52E+06	4.60E+06	6.12E+06	8.69E-01	1.59E+00	6.68E-01	insig
5,7,4',5'-Tetrahydro-3',6-dimethoxyflavone	C17H14O8	Flavonoids	Flavonoid	1.05E+07	1.14E+07	1.26E+07	5.28E+06	4.77E+06	5.47E+06	1.18E+00	4.49E-01	-1.16E+00	down
Limocitrin	C17H14O8	Flavonoids	Flavonoid	1.66E+05	1.24E+05	1.38E+05	3.72E+05	3.17E+05	3.49E+05	1.17E+00	2.42E+00	1.28E+00	up
Laciniatin	C17H14O8	Flavonoids	Flavonols	1.28E+05	1.18E+05	1.31E+05	4.25E+05	3.62E+05	3.33E+05	1.19E+00	2.97E+00	1.57E+00	up
5-Hydroxy-6,7,3',4'-tetramethoxyflavone	C19H18O7	Flavonoids	Flavonoid	3.27E+05	4.21E+05	3.85E+05	2.48E+04	2.29E+04	2.39E+04	1.20E+00	6.32E-02	-3.98E+00	down
Eupatorin-5-methylether	C19H18O7	Flavonoids	Flavonoid	2.41E+05	3.36E+05	2.96E+05	2.03E+04	1.60E+04	1.98E+04	1.20E+00	6.43E-02	-3.96E+00	down
Chrysosplenol D	C18H16O8	Flavonoids	Flavonols	2.79E+07	4.23E+07	4.85E+07	2.22E+07	2.33E+07	2.81E+07	9.46E-01	6.19E-01	-6.92E-01	insig
Centaureidin (5,7,3'-Trihydroxy-3,6,4'-Trimethoxyflavone)	C18H16O8	Flavonoids	Flavonoid	5.72E+04	1.22E+05	1.18E+05	6.42E+04	5.98E+04	7.92E+04	6.44E-01	6.83E-01	-5.49E-01	insig
Acerosin	C18H16O8	Flavonoids	Flavonoid	9.08E+06	1.58E+07	1.92E+07	7.70E+06	8.05E+06	9.87E+06	8.77E-01	5.81E-01	-7.83E-01	insig
5,4′-Dihydroxy-3,6,7,3′-tetramethoxyflavone	C19H18O8	Flavonoids	Flavonoid	1.77E+07	3.18E+07	3.31E+07	2.06E+07	2.08E+07	2.37E+07	5.28E-01	7.88E-01	-3.43E-01	insig
Casticin	C19H18O8	Flavonoids	Dihydroflavone	2.46E+06	4.44E+06	4.49E+06	9.99E+05	1.00E+06	1.11E+06	1.14E+00	2.73E-01	-1.87E+00	down
Arteanoflavone	C19H18O8	Flavonoids	Flavonoid	1.80E+07	3.32E+07	3.39E+07	2.12E+07	2.12E+07	2.33E+07	5.57E-01	7.73E-01	-3.71E-01	insig
5-Hydroxyauranetin	C20H20O8	Flavonoids	Flavonols	6.05E+05	1.01E+06	9.34E+05	3.57E+03	2.29E+03	3.06E+03	1.20E+00	3.49E-03	-8.16E+00	down
5-Hydroxy-6,7,8,3',4'-pentamethoxyflavone	C20H20O8	Flavonoids	Flavonoid	1.29E+06	2.14E+06	1.95E+06	3.43E+03	2.54E+03	3.15E+03	1.20E+00	1.70E-03	-9.20E+00	down
Artemetin	C20H20O8	Flavonoids	Flavonoid	7.30E+05	1.25E+06	1.12E+06	1.64E+03	2.09E+03	2.45E+03	1.20E+00	1.99E-03	-8.97E+00	down
Apigenin-8-C-Arabinoside	C20H18O9	Flavonoids	Flavonoid	9.35E+04	2.29E+05	1.72E+05	9.00E+00	9.00E+00	9.00E+00	1.20E+00	5.46E-05	-1.42E+01	down
Chrysin-7-O-glucoside	C21H20O9	Flavonoids	Flavonoid	2.28E+04	1.86E+04	1.83E+04	1.49E+04	1.58E+04	1.19E+04	1.00E+00	7.15E-01	-4.85E-01	insig
Isovitexin	C21H20O10	Flavonoids	Flavonoid carbonoside	3.09E+06	6.38E+06	7.03E+06	1.30E+06	1.53E+06	1.62E+06	1.11E+00	2.70E-01	-1.89E+00	down
Galangin-7-O-glucoside	C21H20O10	Flavonoids	Flavonoid	9.13E+05	1.87E+06	2.03E+06	8.56E+04	9.96E+04	9.00E+00	8.27E-01	3.86E-02	-4.70E+00	insig
Genistin	C21H20O10	Flavonoids	Isoflavones	3.78E+03	1.04E+04	1.58E+04	9.00E+00	9.00E+00	9.00E+00	1.19E+00	9.03E-04	-1.01E+01	down
Genistein-7-O-galactoside	C21H20O10	Flavonoids	Isoflavones	5.91E+06	1.58E+07	1.37E+07	8.28E+05	9.00E+05	2.04E+06	1.13E+00	1.06E-01	-3.23E+00	down
Genistein-8-C-glucoside	C21H20O10	Flavonoids	Isoflavones	6.33E+06	1.25E+07	1.27E+07	2.56E+06	2.66E+06	3.06E+06	1.13E+00	2.63E-01	-1.93E+00	down
Vitexin	C21H20O10	Flavonoids	Flavonoid carbonoside	2.02E+06	4.16E+06	4.00E+06	7.70E+05	1.02E+06	1.06E+06	1.11E+00	2.80E-01	-1.84E+00	down
Naringenin-4'-O-glucoside	C21H22O10	Flavonoids	Flavonoid	1.06E+05	2.21E+05	2.15E+05	4.38E+03	9.41E+03	9.92E+03	1.17E+00	4.37E-02	-4.52E+00	down
Butin-7-O-glucoside	C21H22O10	Flavonoids	Flavonoid	1.05E+05	2.03E+05	2.30E+05	9.00E+00	9.00E+00	9.00E+00	1.20E+00	5.02E-05	-1.43E+01	down
Baicalin	C21H18O11	Flavonoids	Flavonoid	1.26E+07	4.82E+07	5.73E+07	2.82E+06	2.91E+06	6.51E+06	1.07E+00	1.04E-01	-3.27E+00	down
Apigenin-7-O-glucuronide	C21H18O11	Flavonoids	Flavonoid	1.51E+06	5.83E+06	6.83E+06	4.40E+05	5.95E+05	9.50E+05	1.04E+00	1.40E-01	-2.84E+00	down
Acacetin-7-O-galactoside	C22H22O10	Flavonoids	Flavonoid	6.72E+04	5.48E+04	9.00E+00	1.15E+04	1.70E+04	3.07E+04	3.48E-01	4.85E-01	-1.04E+00	insig
Tilianin	C22H22O10	Flavonoids	Flavonoid	5.32E+04	3.71E+04	9.00E+00	1.36E+04	1.37E+04	1.75E+04	3.62E-01	4.96E-01	-1.01E+00	insig
Isoorientin	C21H20O11	Flavonoids	Flavonoid carbonoside	7.56E+05	7.90E+05	9.63E+05	2.19E+06	1.90E+06	2.13E+06	1.18E+00	2.48E+00	1.31E+00	up
Luteolin-3'-O-glucoside	C21H20O11	Flavonoids	Flavonoid	9.00E+00	9.00E+00	9.00E+00	1.69E+06	1.08E+06	3.09E+06	1.20E+00	2.17E+05	1.77E+01	up
Orientin	C21H20O11	Flavonoids	Flavonoid carbonoside	1.47E+06	1.80E+06	1.81E+06	3.72E+06	3.43E+06	4.22E+06	1.17E+00	2.23E+00	1.16E+00	up
Cynaroside	C21H20O11	Flavonoids	Flavonoid	2.13E+07	1.75E+07	2.08E+07	4.22E+06	4.19E+06	5.90E+06	1.18E+00	2.40E-01	-2.06E+00	down
Kaempferol-4'-O-glucoside	C21H20O11	Flavonoids	Flavonoid	2.10E+07	1.67E+07	1.81E+07	4.46E+06	4.25E+06	5.87E+06	1.18E+00	2.61E-01	-1.94E+00	down
Luteolin-4'-O-glucoside	C21H20O11	Flavonoids	Flavonoid	9.00E+00	9.00E+00	9.00E+00	1.68E+06	1.19E+06	3.13E+06	1.20E+00	2.22E+05	1.78E+01	up
Kuromanin	C21H21O11+	Flavonoids	Anthocyanins	4.79E+04	5.64E+04	3.48E+04	9.00E+00	9.00E+00	9.00E+00	1.20E+00	1.94E-04	-1.23E+01	down

Marein	C21H22O11	Flavonoids	Chalcones	7.96E+05	6.25E+05	6.65E+05	2.01E+05	1.43E+05	2.35E+05	1.16E+00	2.78E-01	-1.85E+00	down
Acacetin-7-O-glucuronide	C26H20O8	Flavonoids	Flavonoid	2.22E+04	1.79E+04	3.89E+03	4.00E+03	4.46E+03	5.12E+03	7.73E-01	3.08E-01	-1.70E+00	insig
Scutellarin	C21H18O12	Flavonoids	Flavonoid	6.22E+06	1.73E+07	1.87E+07	5.78E+06	6.28E+06	9.74E+06	7.20E-01	5.16E-01	-9.54E-01	insig
Tetahydroxyflavone-7-O-glucuronide	C21H18O12	Flavonoids	Flavonoid	6.36E+06	1.18E+07	1.50E+07	4.49E+06	4.97E+06	8.14E+06	8.39E-01	5.30E-01	-9.15E-01	insig
Kaempferol-3-O-glucuronide	C21H18O12	Flavonoids	Flavonols	5.37E+06	1.38E+07	1.25E+07	4.86E+06	5.81E+06	8.29E+06	6.78E-01	5.98E-01	-7.41E-01	insig
Pratensein-7-O-glucoside	C22H22O11	Flavonoids	Flavonoid	7.21E+03	9.75E+03	9.00E+00	5.69E+04	4.58E+04	7.04E+04	8.21E-01	1.02E+01	3.35E+00	insig
Scoparin	C22H22O11	Flavonoids	Flavonoid	8.82E+04	1.27E+05	1.36E+05	9.13E+05	7.64E+05	8.23E+05	1.19E+00	7.12E+00	2.83E+00	up
Diosmetin-7-O-galactoside	C22H22O11	Flavonoids	Flavonoid	2.84E+06	3.17E+06	3.75E+06	6.50E+05	6.44E+05	8.28E+05	1.19E+00	2.17E-01	-2.20E+00	down
6-C-MethylKaempferol-3-glucoside	C22H22O11	Flavonoids	Flavonoid	2.58E+06	2.95E+06	3.98E+06	6.58E+05	6.91E+05	8.30E+05	1.18E+00	2.29E-01	-2.13E+00	down
Diosmetin-6-C-glucoside	C22H22O11	Flavonoids	Flavonoid	6.21E+04	9.17E+04	1.12E+05	6.64E+05	5.61E+05	5.70E+05	1.18E+00	6.75E+00	2.75E+00	up
Hispidulin-7-O-Glucoside	C22H22O11	Flavonoids	Flavonoid	2.57E+06	3.57E+06	3.67E+06	7.58E+05	6.88E+05	7.86E+05	1.18E+00	2.28E-01	-2.14E+00	down
6-Hydroxykaempferol-7-O-glucoside	C21H20O12	Flavonoids	Flavonols	5.83E+05	1.07E+06	9.08E+05	1.91E+06	1.62E+06	3.63E+06	1.02E+00	2.79E+00	1.48E+00	up
Isohyperoside	C21H20O12	Flavonoids	Flavonols	5.88E+06	7.84E+06	6.17E+06	2.69E+06	2.93E+06	6.37E+06	8.50E-01	6.03E-01	-7.31E-01	insig
Apigenin-7-O-(6"-acetyl)glucoside	C23H22O11	Flavonoids	Flavonoid	8.74E+03	1.62E+04	1.14E+04	2.24E+03	9.00E+00	9.00E+00	9.78E-01	6.21E-02	-4.01E+00	insig
Diosmetin-7-O-glucuronide	C22H20O12	Flavonoids	Flavonoid	2.80E+06	5.42E+06	4.48E+06	1.25E+06	1.44E+06	1.23E+06	1.13E+00	3.08E-01	-1.70E+00	down
6-Methoxykaempferol-3-O-glucoside	C22H22O12	Flavonoids	Flavonoid	4.24E+05	9.33E+05	1.06E+06	7.28E+05	6.20E+05	1.00E+06	4.51E-02	9.71E-01	-4.20E-02	insig
5,7,4'-Trihydroxy-6,8-dimethoxyisoflavone-7-O-galactoside	C23H24O12	Flavonoids	Isoflavones	2.78E+06	3.89E+06	4.63E+06	8.22E+05	6.03E+05	6.95E+05	1.17E+00	1.88E-01	-2.41E+00	down
Tricin-7-O-Glucoside	C23H24O12	Flavonoids	Flavonoid	2.73E+06	4.22E+06	4.82E+06	8.19E+05	6.46E+05	6.39E+05	1.17E+00	1.79E-01	-2.48E+00	down
Jaceosidin-7-O-Glucoside	C23H24O12	Flavonoids	Flavonoid	2.81E+06	4.57E+06	4.70E+06	8.91E+05	6.11E+05	6.78E+05	1.17E+00	1.80E-01	-2.47E+00	down
Patuletin-3-O-glucoside	C22H22O13	Flavonoids	Flavonoid	2.36E+04	4.04E+04	2.86E+04	1.94E+05	7.96E+04	1.21E+05	1.11E+00	4.25E+00	2.09E+00	up
Chrysoeriol-7-O-(6"-acetyl)glucoside	C24H24O12	Flavonoids	Flavonoid	4.61E+05	6.67E+05	7.87E+05	6.16E+05	4.82E+05	6.06E+05	3.23E-01	8.90E-01	-1.69E-01	insig
Quercetin-3-O-(6"-acetyl)glucoside	C23H22O13	Flavonoids	Flavonols	2.07E+03	3.48E+03	1.03E+03	9.00E+00	9.00E+00	9.00E+00	1.19E+00	4.10E-03	-7.93E+00	down
Tricin-7-O-Glucuronide	C23H22O13	Flavonoids	Flavonoid	1.97E+05	1.60E+05	2.54E+05	7.30E+04	1.55E+05	1.15E+05	9.29E-01	5.62E-01	-8.32E-01	insig
Eupatilin-7-O-glucoside	C24H26O12	Flavonoids	Flavonoid	1.48E+06	1.54E+06	1.62E+06	1.55E+06	1.26E+06	1.38E+06	7.33E-01	9.03E-01	-1.47E-01	insig
Limocitrin-3-O-galactoside	C23H24O13	Flavonoids	Flavonoid	2.57E+05	2.55E+05	3.80E+05	4.56E+06	3.79E+06	3.26E+06	1.19E+00	1.30E+01	3.70E+00	up
Syringetin-7-O-glucoside	C23H24O13	Flavonoids	Flavonoid	3.39E+05	7.34E+05	6.58E+05	1.04E+06	9.08E+05	8.25E+05	8.67E-01	1.60E+00	6.80E-01	insig
5,6,3′,4′-Tetrahydroxy-3,7-dimethoxyflavone-6-O-glucoside	C23H24O13	Flavonoids	Flavonoid	2.49E+05	3.34E+05	3.91E+05	4.41E+06	3.70E+06	3.30E+06	1.19E+00	1.17E+01	3.55E+00	up
6"-O-Malonylgenistin	C24H22O13	Flavonoids	Isoflavones	3.91E+06	7.82E+06	6.66E+06	6.85E+05	9.95E+05	1.35E+06	1.15E+00	1.65E-01	-2.60E+00	down
Apigenin-7-O-(6"-malonyl)glucoside	C24H22O13	Flavonoids	Flavonoid	2.37E+05	4.70E+05	4.35E+05	4.75E+04	6.27E+04	7.58E+04	1.16E+00	1.63E-01	-2.62E+00	down
Iridin	C24H26O13	Flavonoids	Isoflavones	3.42E+04	7.76E+04	1.23E+05	2.09E+05	1.85E+05	1.41E+05	9.19E-01	2.28E+00	1.19E+00	insig
Chrysosplenoside C	C24H26O13	Flavonoids	Flavonoid	3.74E+05	6.54E+05	6.01E+05	5.06E+05	4.82E+05	4.40E+05	3.38E-01	8.76E-01	-1.91E-01	insig
Kaempferol-3-O-(6"-malonyl)galactoside	C24H22O14	Flavonoids	Flavonols	1.90E+06	2.17E+06	2.12E+06	4.43E+05	5.35E+05	5.13E+05	1.20E+00	2.41E-01	-2.05E+00	down
Luteolin-7-O-(6"-malonyl)glucoside	C24H22O14	Flavonoids	Flavonoid	8.33E+04	1.26E+05	1.00E+05	1.93E+04	2.28E+04	2.98E+04	1.17E+00	2.32E-01	-2.11E+00	down
Apigenin-6-C-arabinoside-8-C-xyloside	C25H26O13	Flavonoids	Flavonoid carbonoside	3.67E+06	3.95E+06	3.80E+06	9.00E+00	9.00E+00	9.00E+00	1.20E+00	2.36E-06	-1.87E+01	down
Apigenin-6-C-xyloside-8-C-arabinoside	C25H26O13	Flavonoids	Flavonoid carbonoside	1.16E+05	1.69E+05	1.26E+05	9.00E+00	9.00E+00	9.00E+00	1.20E+00	6.56E-05	-1.39E+01	down
Okanin-4'-O-(6"-O-malonyl)glucoside	C24H24O14	Flavonoids	Chalcones	1.20E+05	1.17E+05	8.89E+04	3.69E+04	4.49E+04	4.23E+04	1.17E+00	3.81E-01	-1.39E+00	down
Chrysoeriol-7-O-(6"-malonyl)glucoside	C25H24O14	Flavonoids	Flavonoid	1.06E+06	9.18E+05	1.07E+06	5.45E+05	7.07E+05	7.49E+05	1.08E+00	6.57E-01	-6.06E-01	insig
Diosmetin-7-O-(6"-malonyl)glucoside	C25H24O14	Flavonoids	Flavonoid	1.30E+05	1.23E+05	1.55E+05	6.51E+04	7.06E+04	8.06E+04	1.16E+00	5.30E-01	-9.15E-01	insig
Kaempferide-3-O-(6"-malonyl)glucoside	C25H24O14	Flavonoids	Flavonoid	6.12E+04	6.71E+04	2.40E+04	1.43E+06	1.41E+06	1.59E+06	1.18E+00	2.91E+01	4.86E+00	up
Maohuoside A	C27H32O12	Flavonoids	Flavonols	2.54E+05	2.26E+05	2.95E+05	2.08E+05	1.64E+05	1.23E+05	9.70E-01	6.37E-01	-6.50E-01	insig
Quercetin-7-O-(6"-malonyl)glucoside	C24H22O15	Flavonoids	Flavonols	6.52E+05	5.85E+05	5.02E+05	3.43E+05	3.75E+05	8.70E+05	3.39E-01	9.13E-01	-1.32E-01	insig
Isorhamnetin-3-O-(6"-malonylglucoside)	C25H24O15	Flavonoids	Flavonoid	1.44E+04	1.22E+05	1.52E+05	9.77E+04	5.69E+04	1.19E+05	2.29E-01	9.50E-01	-7.38E-02	insig
Tamarixetin-3-O-(6"-malonyl)glucoside	C25H24O15	Flavonoids	Flavonoid	7.00E+05	1.33E+06	8.97E+05	2.14E+06	1.49E+06	1.53E+06	9.68E-01	1.76E+00	8.16E-01	insig
Schaftoside	C26H28O14	Flavonoids	Flavonoid carbonoside	37764	54509	58465	9	9	9	1.200569285	0.000179119	-12.4467961	down
Hispidulin-8-C-(2"-O-xylosyl)xyloside	C26H28O14	Flavonoids	Flavonoid carbonoside	424810	390180	384390	303660	386860	425610	0.440987344	0.930589138	-0.10378375	insig
Apigenin-8-C-(2"-xylosyl)glucoside	C26H28O14	Flavonoids	Flavonoid carbonoside	2848400	3544800	3364000	271470	192080	332120	1.188735866	0.08154696	-3.61622509	down
Apigenin-6-C-(2"-glucosyl)arabinoside	C26H28O14	Flavonoids	Flavonoid carbonoside	21834000	27460000	27257000	2364300	1538400	2513600	1.188377102	0.083817324	-3.57660772	down
Tricin-7-O-(6"-O-malonyl)glucoside	C26H26O15	Flavonoids	Flavonoid	1.81E+05	1.43E+05	1.11E+05	4.71E+04	4.92E+04	4.36E+04	1.16E+00	3.22E-01	-1.64E+00	down
Rhoifolin	C27H30O14	Flavonoids	Flavonoid	1.26E+07	2.50E+07	2.87E+07	1.35E+06	1.44E+06	1.57E+06	1.18E+00	6.58E-02	-3.93E+00	down
Genistein-7-O-galactoside-rhamnose	C27H30O14	Flavonoids	Isoflavones	1.39E+07	2.65E+07	2.75E+07	1.16E+06	1.45E+06	1.83E+06	1.18E+00	6.56E-02	-3.93E+00	down
Isorhoifolin	C27H30O14	Flavonoids	Flavonoid	1.14E+07	2.81E+07	2.84E+07	1.39E+06	1.58E+06	1.87E+06	1.17E+00	7.13E-02	-3.81E+00	down
Luteolin-7-O-glucoside-5-O-arabinoside	C26H28O15	Flavonoids	Flavonoid	4.04E+03	6.25E+03	9.19E+03	1.28E+04	1.05E+04	1.43E+04	9.72E-01	1.93E+00	9.47E-01	insig
Lonicerin	C27H30O15	Flavonoids	Flavonoid	6.22E+06	1.39E+07	1.14E+07	4.06E+06	3.16E+06	5.02E+06	1.02E+00	3.88E-01	-1.36E+00	down
Vitexin-2"-O-glucoside	C27H30O15	Flavonoids	Flavonoid carbonoside	190860	511740	473050	485740	489460	523460	0.55336775	1.274750138	0.350214494	insig
Apigenin-6,8-di-C-glucoside (Vicenin-2)	C27H30O15	Flavonoids	Flavonoid	1.28E+06	2.09E+06	1.84E+06	5.75E+06	5.96E+06	6.84E+06	1.17E+00	3.56E+00	1.83E+00	up
Saponarin(Isovitexin-7-O-glucoside)	C27H30O15	Flavonoids	Flavonoid	3.44E+05	5.21E+05	4.95E+05	2.89E+05	3.74E+05	3.02E+05	8.92E-01	7.09E-01	-4.96E-01	insig
Luteolin-7-O-rutinoside	C27H30O15	Flavonoids	Flavonoid	7.42E+06	1.79E+07	1.57E+07	4.73E+06	4.22E+06	5.39E+06	1.04E+00	3.49E-01	-1.52E+00	down
Diosmin	C28H32O15	Flavonoids	Flavonoid	9.73E+06	1.23E+07	1.69E+07	2.08E+05	2.07E+05	2.32E+05	1.20E+00	1.66E-02	-5.91E+00	down
Neodiosmin	C28H32O15	Flavonoids	Flavonoid	5.33E+06	6.33E+06	1.19E+07	1.08E+05	1.04E+05	9.73E+04	1.19E+00	1.32E-02	-6.25E+00	down
Hispidulin-7-O-(6"-O-p-Coumaroyl)Glucoside	C31H28O13	Flavonoids	Flavonoid	5.57E+06	6.90E+06	1.00E+07	1.30E+05	1.07E+05	1.22E+05	1.20E+00	1.60E-02	-5.97E+00	down
Luteolin-7-O-gentiobioside	C27H30O16	Flavonoids	Flavonoid	1.68E+07	1.53E+07	1.78E+07	1.47E+07	1.41E+07	1.74E+07	5.53E-01	9.25E-01	-1.12E-01	insig
Isoorientin-7-O-glucoside	C27H30O16	Flavonoids	Flavonoid carbonoside	30092	25212	24608	150340	164910	171840	1.196659092	6.095329863	2.607704298	up
Luteolin-6,8-di-C-glucoside	C27H30O16	Flavonoids	Flavonoid carbonoside	28258	37543	14510	2438200	2089600	2150400	1.193306063	83.1542379	6.377717886	up
Quercetin-3-O-glucoside-7-O-rhamnoside	C27H30O16	Flavonoids	Flavonols	1.88E+06	3.27E+06	2.55E+06	1.02E+07	8.57E+06	1.96E+07	1.12E+00	4.97E+00	2.31E+00	up
Quercetin-3-O-robinobioside	C27H30O16	Flavonoids	Flavonols	8.92E+05	1.37E+06	1.15E+06	5.19E+06	4.57E+06	1.08E+07	1.14E+00	6.04E+00	2.59E+00	up
Okanin-4'-O-gentiobioside	C27H32O16	Flavonoids	Chalcones	3.68E+05	3.63E+05	4.05E+05	3.88E+05	3.46E+05	4.61E+05	2.95E-01	1.05E+00	7.41E-02	insig
Apigenin-7-O-(2"-glucurosyl)glucuronide	C27H26O17	Flavonoids	Flavonoid	1.04E+06	4.66E+06	5.56E+06	9.75E+03	1.36E+04	1.68E+04	1.18E+00	3.56E-03	-8.13E+00	down
Kaempferol-3-O-glucuronide-7-O-glucoside	C27H28O17	Flavonoids	Flavonols	4.30E+03	2.72E+04	1.21E+04	6.73E+03	1.22E+04	1.45E+04	6.23E-02	7.66E-01	-3.85E-01	insig
Chrysoeriol-6,8-di-C-glucoside	C28H32O16	Flavonoids	Flavonoid carbonoside	4.99E+04	6.67E+04	7.20E+04	2.66E+06	2.63E+06	3.27E+06	1.20E+00	4.54E+01	5.50E+00	up
Tamarixetin-3-O-glucoside-7-O-rhamnoside	C28H32O16	Flavonoids	Flavonoid	2.24E+05	3.01E+05	3.25E+05	2.28E+06	2.04E+06	2.33E+06	1.19E+00	7.83E+00	2.97E+00	up
2'-Hydoxy,5-methoxyGenistein-O-rhamnosyl-glucoside	C28H32O16	Flavonoids	Isoflavones	2.36E+05	3.14E+05	2.90E+05	2.50E+06	2.00E+06	2.32E+06	1.20E+00	8.12E+00	3.02E+00	up
Quercetin-3-O-(2"-O-galactosyl)glucoside	C27H30O17	Flavonoids	Flavonols	2.24E+04	3.74E+04	1.57E+04	3.63E+04	3.17E+04	2.75E+04	5.93E-01	1.26E+00	3.37E-01	insig
Chrysoeriol-7-O-(2"-O-glucuronyl)glucuronide	C28H28O18	Flavonoids	Flavonoid	6.78E+05	6.44E+05	7.30E+05	1.13E+04	1.46E+04	7.47E+03	1.20E+00	1.63E-02	-5.94E+00	down
Syringetin-3-O-rutinoside	C29H34O17	Flavonoids	Flavonoid	2.11E+05	4.12E+05	3.57E+05	3.47E+04	1.43E+04	3.02E+04	1.16E+00	8.08E-02	-3.63E+00	down
Tricin-4'-O-glucoside-7-O-glucoside	C29H34O17	Flavonoids	Flavonoid	1.08E+05	2.16E+05	2.98E+05	2.40E+04	2.15E+04	2.60E+04	1.15E+00	1.15E-01	-3.12E+00	down
5,7,4'-Trihydroxy-6,8-dimethoxyisoflavone-7-O-galactoside-glucoside	C29H34O17	Flavonoids	Isoflavones	2.59E+05	8.93E+05	1.25E+06	7.62E+04	8.90E+04	9.38E+04	1.09E+00	1.08E-01	-3.21E+00	down
Tricin-5,7-O-diglucoside	C29H34O17	Flavonoids	Flavonoid	2.68E+05	1.05E+06	1.04E+06	7.70E+04	7.40E+04	8.97E+04	1.10E+00	1.02E-01	-3.29E+00	down
Laciniatin-7-O-Gentiobioside	C29H34O18	Flavonoids	Flavonols	3.43E+05	5.90E+05	7.06E+05	8.89E+04	6.08E+04	7.22E+04	1.17E+00	1.35E-01	-2.88E+00	down
Tricin-7-O-(2"-Sinapoyl)glucuronide	C34H32O17	Flavonoids	Flavonoid	1.27E+04	6.19E+04	9.09E+04	1.02E+04	8.40E+03	1.27E+04	9.04E-01	1.89E-01	-2.40E+00	insig
6-MethylCoumarin	C10H8O2	Lignans and Coumarins	Coumarins	26787	22600	27603	8507.8	3074.6	5251.8	1.119414101	0.218654371	-2.19327591	down
Umbelliferone	C9H6O3	Lignans and Coumarins	Coumarins	2.11E+05	2.43E+05	2.16E+05	1.79E+05	1.82E+05	1.97E+05	1.04E+00	8.32E-01	-2.65E-01	insig
6-Hydroxy-4-methylcoumarin	C10H8O3	Lignans and Coumarins	Coumarins	6.66E+04	5.70E+04	8.75E+04	1.57E+04	1.70E+04	1.04E+04	1.17E+00	2.04E-01	-2.29E+00	down
Daphnetin	C9H6O4	Lignans and Coumarins	Coumarins	9.35E+04	9.56E+04	5.98E+04	2.14E+06	2.13E+06	2.87E+06	1.20E+00	2.87E+01	4.84E+00	up
Isoscopoletin (6-Hydroxy-7-Methoxycoumarin)	C10H8O4	Lignans and Coumarins	Coumarins	9.00E+00	9.00E+00	9.00E+00	7.55E+05	6.91E+05	9.54E+05	1.20E+00	8.89E+04	1.64E+01	up
Scoparone	C11H10O4	Lignans and Coumarins	Coumarins	9.00E+00	9.00E+00	9.00E+00	1.99E+05	2.20E+05	3.01E+05	1.20E+00	2.67E+04	1.47E+01	up
Fraxidin (8-Hydroxy-6,7-dimethoxycoumarin)	C11H10O5	Lignans and Coumarins	Coumarins	6.63E+04	9.65E+04	9.01E+04	1.15E+05	8.40E+04	9.63E+04	5.83E-01	1.17E+00	2.26E-01	insig
Isofraxidin*	C11H10O5	Lignans and Coumarins	Coumarins	1.37E+05	2.44E+05	2.18E+05	2.76E+05	2.57E+05	2.57E+05	7.84E-01	1.32E+00	3.99E-01	insig
Umckalin (7-hydroxy-5,6-dimethoxycoumarin)	C11H10O5	Lignans and Coumarins	Coumarins	4.92E+05	1.17E+06	6.19E+05	2.58E+05	2.32E+05	2.36E+05	1.08E+00	3.19E-01	-1.65E+00	down
Demethylwedelolactone	C15H8O7	Lignans and Coumarins	Coumarins	9.00E+00	3.38E+04	3.53E+04	1.69E+04	2.01E+04	1.86E+04	4.31E-01	8.04E-01	-3.14E-01	insig
Tachioside	C13H18O8	Lignans and Coumarins	Lignans	3.61E+04	2.10E+04	4.65E+04	2.41E+04	2.54E+04	2.14E+04	6.89E-01	6.85E-01	-5.47E-01	insig
Skimmin	C15H16O8	Lignans and Coumarins	Coumarins	1.23E+06	1.77E+06	1.45E+06	1.32E+06	2.13E+06	1.21E+06	7.07E-02	1.05E+00	6.90E-02	insig
Esculin	C15H16O9	Lignans and Coumarins	Coumarins	3.25E+06	3.60E+06	2.43E+06	1.43E+07	1.53E+07	1.57E+07	1.19E+00	4.88E+00	2.29E+00	up
3,7-DimethylQuercetagetin-(3',4',5,6-Tetrahydroxy-3,7-dimethoxyflavone)	C17H14O8	Lignans and Coumarins	Coumarins	1.65E+04	3.65E+04	2.77E+04	9.00E+00	9.00E+00	9.00E+00	1.20E+00	3.35E-04	-1.15E+01	down
Epipinoresinol	C20H22O6	Lignans and Coumarins	Lignans	3.82E+05	7.09E+05	1.00E+06	1.59E+05	7.51E+04	1.21E+05	1.11E+00	1.70E-01	-2.56E+00	down
Pinoresinol	C20H22O6	Lignans and Coumarins	Lignans	4.23E+05	7.51E+05	1.04E+06	1.62E+05	8.31E+04	1.22E+05	1.13E+00	1.65E-01	-2.60E+00	down
Scopoletin-7-O-glucuronide	C16H16O10	Lignans and Coumarins	Coumarins	1.69E+07	2.52E+07	2.14E+07	2.11E+07	1.59E+07	2.28E+07	2.14E-01	9.39E-01	-9.02E-02	insig
Arctigenin	C21H24O6	Lignans and Coumarins	Lignans	1.76E+04	2.13E+04	2.50E+04	9.00E+00	9.00E+00	4.19E+03	9.77E-01	6.57E-02	-3.93E+00	insig
Isolariciresinol-9'-O-glucoside	C26H34O11	Lignans and Coumarins	Lignans	5.91E+04	6.93E+04	7.47E+04	5.19E+04	3.74E+04	4.41E+04	1.05E+00	6.57E-01	-6.07E-01	insig

Secoisolariciresinol 4-O-glucoside	C26H36O11	Lignans and Coumarins	Lignans	9.36E+05	2.26E+06	1.43E+06	7.57E+05	6.66E+05	9.13E+05	9.06E-01	5.05E-01	-9.85E-01	insig
Phillyrin	C27H34O11	Lignans and Coumarins	Lignans	1.40E+05	9.59E+04	9.99E+04	1.10E+06	1.09E+06	1.12E+06	1.19E+00	9.84E+00	3.30E+00	up
Arctiin	C27H34O11	Lignans and Coumarins	Lignans	9.00E+00	9.00E+00	9.00E+00	9.52E+05	1.01E+06	9.11E+05	1.20E+00	1.06E+05	1.67E+01	up
5'-Methoxymatairesinoside	C27H34O12	Lignans and Coumarins	Lignans	8.67E+06	1.08E+07	1.17E+07	1.52E+07	1.34E+07	1.48E+07	1.04E+00	1.39E+00	4.73E-01	insig
Eucommin A	C27H34O12	Lignans and Coumarins	Lignans	8.65E+06	1.09E+07	1.03E+07	1.23E+07	1.27E+07	1.41E+07	1.04E+00	1.31E+00	3.92E-01	insig
Lappaol C	C30H34O10	Lignans and Coumarins	Lignans	9.60E+03	1.43E+04	1.72E+04	9.00E+00	9.00E+00	9.00E+00	1.20E+00	6.57E-04	-1.06E+01	down
Pinoresinol-4-O-(6"-acetyl)glucoside	C28H34O12	Lignans and Coumarins	Lignans	2.29E+05	2.03E+05	2.46E+05	6.63E+05	8.67E+05	6.72E+05	1.18E+00	3.25E+00	1.70E+00	up
Nitensoside B	C28H32O16	Lignans and Coumarins	Coumarins	2.29E+05	2.92E+05	2.60E+05	2.56E+06	2.15E+06	2.22E+06	1.20E+00	8.88E+00	3.15E+00	up
2-Aminoethylphosphonate	C2H8NO3P	Lipids	Glycerol ester	6.23E+04	5.21E+04	4.94E+04	4.82E+04	6.15E+04	4.05E+04	3.98E-01	9.16E-01	-1.26E-01	insig
cis-3-Hexenyl acetate	C8H14O2	Lipids	Free fatty acids	2.49E+05	2.19E+05	2.37E+05	2.73E+05	2.89E+05	2.76E+05	1.08E+00	1.19E+00	2.48E-01	insig
Undecylic Acid	C11H22O2	Lipids	Free fatty acids	4.54E+05	4.73E+05	4.89E+05	5.96E+05	6.40E+05	8.23E+05	1.05E+00	1.45E+00	5.39E-01	insig
Dodecanoic acid	C12H24O2	Lipids	Free fatty acids	6.64E+04	6.42E+04	6.83E+04	7.91E+04	9.05E+04	1.12E+05	1.02E+00	1.41E+00	5.01E-01	insig
δ-Tridecalactone	C13H24O2	Lipids	Glycerol ester	1.93E+04	1.68E+04	1.72E+04	1.73E+04	2.82E+04	1.77E+04	4.72E-01	1.18E+00	2.45E-01	insig
12-Hydroxydodecanoic acid	C12H24O3	Lipids	Free fatty acids	3.63E+03	2.64E+03	1.50E+03	1.93E+03	2.69E+03	3.90E+03	2.04E-01	1.09E+00	1.30E-01	insig
Myristoleic acid	C14H26O2	Lipids	Free fatty acids	1.59E+03	2.69E+03	2.54E+03	2.93E+03	2.61E+03	3.98E+03	7.64E-01	1.40E+00	4.82E-01	insig
Myristic Acid	C14H28O2	Lipids	Free fatty acids	1.04E+07	1.09E+07	1.05E+07	1.18E+07	1.33E+07	1.34E+07	1.09E+00	1.21E+00	2.80E-01	insig
Pentadecanoic Acid	C15H30O2	Lipids	Free fatty acids	1.28E+05	1.67E+05	1.21E+05	1.14E+05	1.51E+05	1.31E+05	2.16E-01	9.51E-01	-7.25E-02	insig
Palmitoleic Acid	C16H30O2	Lipids	Free fatty acids	5.91E+03	7.87E+03	3.21E+03	5.93E+03	5.55E+03	8.04E+03	3.90E-01	1.15E+00	2.01E-01	insig
(7Z)-Hexadecenoic acid	C16H30O2	Lipids	Free fatty acids	1.01E+03	1.89E+03	9.57E+02	4.64E+02	8.02E+02	7.15E+02	9.14E-01	5.13E-01	-9.62E-01	insig
Palmitic acid	C16H32O2	Lipids	Free fatty acids	1.70E+04	1.68E+04	1.32E+04	1.23E+04	1.74E+04	1.54E+04	2.08E-01	9.59E-01	-5.97E-02	insig
Choline Alfoscerate	C8H20NO6P	Lipids	PC	3.55E+04	4.29E+04	4.25E+04	6.18E+04	9.38E+04	4.66E+04	8.91E-01	1.67E+00	7.42E-01	insig
Methyl 7,10-hexadecadienoate	C17H30O2	Lipids	Free fatty acids	1.26E+07	6.02E+06	8.34E+06	4.92E+06	4.87E+06	4.19E+06	9.77E-01	5.18E-01	-9.48E-01	insig
10-Heptadecenoic Acid	C17H32O2	Lipids	Free fatty acids	3.17E+05	5.20E+05	4.09E+05	6.67E+05	6.13E+05	5.04E+05	9.03E-01	1.43E+00	5.18E-01	insig
Hexadecylsphingosine	C16H35NO2	Lipids	Sphingolipids	3.74E+06	4.36E+06	3.81E+06	4.54E+06	4.07E+06	3.96E+06	4.89E-01	1.06E+00	7.89E-02	insig
9,12-Octadecadien-6-Ynoic Acid	C18H28O2	Lipids	Free fatty acids	3.43E+04	7.11E+04	7.96E+04	1.43E+05	1.42E+05	1.89E+05	1.05E+00	2.56E+00	1.36E+00	up
Punicic acid (9Z,11E,13Z-octadecatrienoic acid)	C18H30O2	Lipids	Free fatty acids	5.20E+05	5.86E+05	4.65E+05	2.11E+05	2.37E+05	2.12E+05	1.18E+00	4.20E-01	-1.25E+00	down
γ-Linolenic Acid	C18H30O2	Lipids	Free fatty acids	7.21E+06	9.90E+06	8.78E+06	4.81E+06	5.38E+06	4.13E+06	1.11E+00	5.53E-01	-8.54E-01	insig
Octadeca-11E,13E,15Z-trienoic acid	C18H30O2	Lipids	Free fatty acids	6.81E+06	6.42E+06	6.52E+06	7.54E+06	7.08E+06	6.68E+06	8.33E-01	1.08E+00	1.09E-01	insig
Crepenynic acid	C18H30O2	Lipids	Free fatty acids	1.30E+05	1.62E+05	1.57E+05	8.37E+04	9.01E+04	6.69E+04	1.13E+00	5.36E-01	-8.99E-01	insig
α-Linolenic Acid	C18H30O2	Lipids	Free fatty acids	7.39E+06	9.78E+06	8.83E+06	4.65E+06	5.36E+06	4.05E+06	1.13E+00	5.41E-01	-8.86E-01	insig
(9Z,11E)-Octadecadienoic acid	C18H32O2	Lipids	Free fatty acids	2.23E+04	3.13E+04	2.29E+04	1.54E+04	2.06E+04	1.44E+04	9.69E-01	6.59E-01	-6.01E-01	insig
Linoleic acid	C18H32O2	Lipids	Free fatty acids	2.07E+07	3.23E+07	2.70E+07	1.56E+07	2.15E+07	1.44E+07	9.38E-01	6.44E-01	-6.36E-01	insig
Oleamide	C18H35NO	Lipids	Free fatty acids	3.46E+04	2.55E+04	1.01E+05	4.10E+04	1.96E+04	6.60E+04	1.91E-01	7.85E-01	-3.50E-01	insig
11-Octadecanoic acid	C18H34O2	Lipids	Free fatty acids	9.33E+05	1.61E+06	1.35E+06	5.95E+05	6.80E+05	5.62E+05	1.09E+00	4.72E-01	-1.08E+00	down
Oleic acid	C18H34O2	Lipids	Free fatty acids	5.33E+03	3.70E+03	3.92E+03	7.78E+03	4.23E+03	4.31E+03	4.97E-01	1.26E+00	3.34E-01	insig
Elaidic Acid	C18H34O2	Lipids	Free fatty acids	5.60E+06	8.64E+06	7.78E+06	3.74E+06	4.18E+06	3.75E+06	1.10E+00	5.30E-01	-9.16E-01	insig
Stearic Acid	C18H36O2	Lipids	Free fatty acids	5.04E+07	4.77E+07	3.82E+07	4.37E+07	4.14E+07	4.21E+07	3.90E-01	9.34E-01	-9.86E-02	insig
10,16-Dihydroxypalmitic acid	C16H32O4	Lipids	Free fatty acids	1.90E+03	2.61E+03	2.28E+03	4.89E+03	4.29E+03	3.73E+03	1.13E+00	1.90E+00	9.27E-01	insig
4-Oxo-9Z,11Z,13E,15E-Octadecatetraenoic Acid	C18H26O3	Lipids	Free fatty acids	6.71E+04	4.43E+04	4.71E+04	4.50E+04	4.61E+04	4.05E+04	6.36E-01	8.31E-01	-2.67E-01	insig
13-KODE; (9Z,11E)-13-Oxooctadeca-9,11-dienoic acid	C18H30O3	Lipids	Free fatty acids	1.41E+04	2.83E+04	2.48E+04	1.14E+04	1.49E+04	1.38E+04	8.74E-01	5.97E-01	-7.45E-01	insig
13-Hydroxy-6,9,11-octadecatrienoic acid	C18H30O3	Lipids	Free fatty acids	5.58E+04	7.74E+04	7.62E+04	5.95E+04	9.29E+04	5.99E+04	5.23E-03	1.01E+00	2.01E-02	insig
13S-Hydroxy-9Z,11E,15Z-octadecatrienoic acid	C18H30O3	Lipids	Free fatty acids	2.08E+05	2.20E+05	1.52E+05	6.68E+04	8.00E+04	7.49E+04	1.16E+00	3.82E-01	-1.39E+00	down
9-Hydroxy-10,12,15-octadecatrienoic acid	C18H30O3	Lipids	Free fatty acids	1.39E+04	2.75E+04	2.42E+04	1.03E+04	1.17E+04	1.39E+04	9.43E-01	5.47E-01	-8.70E-01	insig
13(S)-HODE;13(S)-Hydroxyoctadeca-9Z,11E-dienoic acid	C18H32O3	Lipids	Free fatty acids	5.37E+05	5.77E+05	5.05E+05	2.38E+05	2.36E+05	2.41E+05	1.19E+00	4.41E-01	-1.18E+00	down
9(10)-EpOME;(9R,10S)-(12Z)-9,10-Epoxyoctadecenoic acid	C18H32O3	Lipids	Free fatty acids	5.54E+04	7.16E+04	7.14E+04	4.35E+04	4.88E+04	4.25E+04	1.08E+00	6.80E-01	-5.57E-01	insig
12,13-Epoxy-9-Octadecenoic Acid	C18H32O3	Lipids	Free fatty acids	2.21E+04	3.19E+04	3.27E+04	1.92E+04	1.78E+04	1.50E+04	1.04E+00	6.00E-01	-7.36E-01	insig
9S-Hydroxy-10E,12Z-octadecadienoic acid	C18H32O3	Lipids	Free fatty acids	5.46E+05	5.96E+05	5.31E+05	2.40E+05	2.55E+05	2.51E+05	1.20E+00	4.46E-01	-1.16E+00	down
Ricinoleic acid	C18H34O3	Lipids	Free fatty acids	1.43E+05	2.04E+05	1.93E+05	9.05E+04	1.01E+05	9.65E+04	1.13E+00	5.34E-01	-9.06E-01	insig
1-Eicosanol	C20H42O	Lipids	Free fatty acids	1.51E+05	2.14E+05	1.82E+05	9.58E+04	9.94E+04	9.33E+04	1.14E+00	5.27E-01	-9.23E-01	insig
3-Dehydrosphinganine	C18H37NO2	Lipids	Sphingolipids	2.11E+04	1.76E+04	1.97E+04	5.45E+04	5.18E+04	5.42E+04	1.19E+00	2.75E+00	1.46E+00	up
2R-Hydroxyoctadecanoic Acid	C18H36O3	Lipids	Free fatty acids	1.75E+04	1.94E+04	1.74E+04	1.71E+04	2.10E+04	2.41E+04	6.17E-01	1.15E+00	1.98E-01	insig
Eicosadienoic acid	C20H36O2	Lipids	Free fatty acids	9.29E+04	1.82E+05	1.85E+05	6.78E+04	7.18E+04	6.41E+04	1.03E+00	4.42E-01	-1.18E+00	down
9-Hydroperoxy-10E,12,15Z-octadecatrienoic acid	C18H30O4	Lipids	Free fatty acids	8.44E+04	1.57E+05	7.39E+04	5.03E+04	2.27E+05	2.67E+04	3.37E-01	9.66E-01	-5.01E-02	insig
13S-Hydroperoxy-6Z,9Z,11E-octadecatrienoic acid	C18H30O4	Lipids	Free fatty acids	1.25E+05	2.45E+05	1.13E+05	8.35E+04	3.74E+05	4.92E+04	2.50E-01	1.05E+00	6.86E-02	insig
Eicosenoic acid	C20H38O2	Lipids	Free fatty acids	9.17E+04	7.07E+04	1.03E+05	1.56E+05	5.47E+04	4.60E+04	2.51E-01	9.67E-01	-4.85E-02	insig
9-Hydroxy-13-oxo-10-octadecenoic Acid	C18H32O4	Lipids	Free fatty acids	4.36E+04	5.00E+04	5.20E+04	7.99E+03	9.06E+03	1.20E+04	1.19E+00	2.00E-01	-2.32E+00	down
7S,8S-DiHODE	C18H32O4	Lipids	Free fatty acids	2.26E+04	2.92E+04	3.52E+04	3.85E+04	3.99E+04	4.13E+04	9.44E-01	1.38E+00	4.61E-01	insig
5S,8R-DiHODE	C18H32O4	Lipids	Free fatty acids	2.52E+05	3.12E+05	2.88E+05	1.10E+06	1.00E+06	1.03E+06	1.20E+00	3.68E+00	1.88E+00	up
9-Hydroxy-12-oxo-15(Z)-octadecenoic acid	C18H32O4	Lipids	Free fatty acids	2.64E+05	3.29E+05	3.04E+05	1.14E+06	1.08E+06	1.11E+06	1.20E+00	3.71E+00	1.89E+00	up
9S-Hydroperoxy-10E,12Z-octadecadienoic acid	C18H32O4	Lipids	Free fatty acids	3.52E+04	3.46E+04	3.39E+04	1.57E+04	1.34E+04	1.58E+04	1.19E+00	4.33E-01	-1.21E+00	down
13S-Hydroperoxy-9Z,11E-octadecadienoic acid	C18H32O4	Lipids	Free fatty acids	1.87E+04	2.36E+04	2.42E+04	7.36E+03	7.12E+03	9.34E+03	1.17E+00	3.58E-01	-1.48E+00	down
12,13-DHOME	C18H34O4	Lipids	Free fatty acids	2.24E+04	2.30E+04	2.63E+04	2.73E+04	2.84E+04	3.08E+04	1.00E+00	1.21E+00	2.72E-01	insig
4-Hydroxysphinganine	C18H39NO3	Lipids	Sphingolipids	1.68E+05	1.81E+05	1.71E+05	2.36E+05	2.21E+05	2.43E+05	1.17E+00	1.35E+00	4.30E-01	insig
9,12,13-Trihydroxy-10,15-octadecadienoic acid	C18H32O5	Lipids	Free fatty acids	3.71E+04	3.16E+04	2.60E+04	4.12E+04	3.47E+04	3.34E+04	6.42E-01	1.15E+00	2.07E-01	insig
9,10,13-Trihydroxy-11-Octadecenoic Acid	C18H34O5	Lipids	Free fatty acids	2.87E+05	2.74E+05	2.75E+05	4.60E+05	4.91E+05	4.27E+05	1.18E+00	1.65E+00	7.23E-01	insig
9,12,13-TriHOME; 9(S),12(S),13(S)-Trihydroxy-10(E)-octadecenoic acid	C18H34O5	Lipids	Free fatty acids	3.75E+05	3.97E+05	3.70E+05	6.46E+05	7.29E+05	6.53E+05	1.19E+00	1.78E+00	8.29E-01	insig
Monopalmitin	C19H38O4	Lipids	Glycerol ester	4.84E+04	5.47E+04	4.34E+04	5.09E+04	4.14E+04	4.55E+04	3.63E-01	9.41E-01	-8.80E-02	insig
8,15-Dihydroxy-5,9,11,13-eicosatetraenoic acid	C20H32O4	Lipids	Free fatty acids	1.19E+04	1.10E+04	9.68E+03	9.00E+00	9.00E+00	9.00E+00	1.20E+00	8.29E-04	-1.02E+01	down
15-Hydroperoxyicosatetraenoic acid	C20H32O4	Lipids	Free fatty acids	3.02E+04	1.92E+04	9.72E+03	4.86E+03	4.78E+03	6.22E+03	1.05E+00	2.68E-01	-1.90E+00	down
1-α-Linolenoyl-glycerol	C21H36O4	Lipids	Glycerol ester	2.02E+06	2.94E+06	2.49E+06	1.16E+06	1.36E+06	9.38E+05	1.12E+00	4.64E-01	-1.11E+00	down
2-α-Linolenoyl-glycerol	C21H36O4	Lipids	Glycerol ester	2.77E+05	4.08E+05	3.15E+05	3.06E+05	3.34E+05	2.64E+05	3.86E-01	9.05E-01	-1.44E-01	insig
1-Linoleoylglycerol	C21H38O4	Lipids	Glycerol ester	4.65E+05	6.63E+05	6.75E+05	4.01E+05	4.12E+05	3.58E+05	1.02E+00	6.50E-01	-6.22E-01	insig
2-Linoleoylglycerol	C21H38O4	Lipids	Glycerol ester	1.61E+05	2.32E+05	2.05E+05	2.16E+05	2.36E+05	1.99E+05	4.45E-01	1.09E+00	1.23E-01	insig
1-Oleoyl-Sn-Glycerol	C21H40O4	Lipids	Glycerol ester	7.20E+04	7.11E+04	5.91E+04	4.49E+04	5.54E+04	5.27E+04	1.01E+00	7.57E-01	-4.03E-01	insig
1-O-p-Hydroxycinnamoyl-3-O-caffeoylglycerol	C21H20O8	Lipids	Glycerol ester	1.89E+04	1.45E+04	1.22E+04	7.52E+04	1.02E+05	9.24E+04	1.18E+00	5.90E+00	2.56E+00	up
LysoPA 16:0	C19H39O7P	Lipids	Glycerol ester	1.69E+04	1.51E+04	1.34E+04	1.06E+04	1.95E+04	1.22E+04	3.16E-01	9.31E-01	-1.04E-01	insig
1,3-O-Dicaffeoylglycerol	C21H20O9	Lipids	Glycerol ester	5.31E+05	4.18E+05	4.91E+05	9.00E+00	9.00E+00	9.00E+00	1.20E+00	1.88E-05	-1.57E+01	down
LysoPE 14:0	C19H40NO7P	Lipids	LPE	8.60E+03	8.05E+03	8.40E+03	6.96E+03	5.35E+03	7.46E+03	9.15E-01	7.90E-01	-3.41E-01	insig
1-O-Feruloyl-3-O-caffeoylglycerol	C22H22O9	Lipids	Glycerol ester	9.00E+00	9.00E+00	9.00E+00	2.18E+06	2.22E+06	2.49E+06	1.20E+00	2.55E+05	1.80E+01	up
LysoPE 15:0	C20H42NO7P	Lipids	LPE	2.69E+04	2.02E+04	2.23E+04	9.86E+03	1.15E+04	9.22E+03	1.16E+00	4.41E-01	-1.18E+00	down
LysoPE 16:3	C21H38NO7P	Lipids	LPE	1.62E+04	8.25E+03	1.30E+03	9.00E+00	9.00E+00	9.00E+00	1.17E+00	1.05E-03	-9.90E+00	down
LysoPE 16:1(2n isomer)	C21H42NO7P	Lipids	LPE	1.98E+04	2.64E+04	3.63E+04	1.68E+04	1.13E+04	1.38E+04	1.01E+00	5.09E-01	-9.76E-01	insig
LysoPE 16:1	C21H42NO7P	Lipids	LPE	8.11E+03	7.91E+03	8.27E+03	6.02E+03	5.51E+03	5.12E+03	1.16E+00	6.85E-01	-5.45E-01	insig
LysoPE 16:0	C21H44NO7P	Lipids	LPE	1.77E+06	1.35E+06	1.32E+06	8.26E+05	9.72E+05	8.51E+05	1.11E+00	5.98E-01	-7.43E-01	insig
LysoPE 16:0(2n isomer)	C21H44NO7P	Lipids	LPE	1.32E+05	1.33E+05	1.08E+05	5.79E+04	6.34E+04	6.27E+04	1.17E+00	4.94E-01	-1.02E+00	down
LysoPC 14:0	C22H46NO7P	Lipids	LPC	1.20E+04	2.06E+04	2.35E+04	5.12E+03	8.35E+03	8.32E+03	1.05E+00	3.89E-01	-1.36E+00	down
LysoPE 18:3(2n isomer)	C23H42NO7P	Lipids	LPE	9.87E+05	1.69E+06	1.96E+06	5.11E+05	5.37E+05	4.52E+05	1.12E+00	3.24E-01	-1.63E+00	down
LysoPE 18:3	C23H42NO7P	Lipids	LPE	2.99E+05	2.57E+05	2.59E+05	1.75E+05	1.58E+05	1.72E+05	1.16E+00	6.20E-01	-6.89E-01	insig
LysoPE 18:2(2n isomer)	C23H44NO7P	Lipids	LPE	4.90E+06	8.27E+06	1.01E+07	3.25E+06	3.37E+06	2.94E+06	1.07E+00	4.10E-01	-1.29E+00	down
LysoPE 18:2	C23H44NO7P	Lipids	LPE	5.83E+05	6.00E+05	6.28E+05	3.61E+05	3.84E+05	4.00E+05	1.19E+00	6.32E-01	-6.61E-01	insig
LysoPE 18:1	C23H46NO7P	Lipids	LPE	4.39E+04	3.76E+04	4.17E+04	2.07E+04	2.07E+04	2.39E+04	1.18E+00	5.30E-01	-9.16E-01	insig
LysoPC 15:1	C23H46NO7P	Lipids	LPC	8.83E+03	1.37E+04	1.84E+04	2.05E+03	3.26E+03	3.07E+03	1.14E+00	2.05E-01	-2.29E+00	down
LysoPE 18:1(2n isomer)	C23H46NO7P	Lipids	LPE	1.54E+05	2.34E+05	2.91E+05	5.19E+04	5.03E+04	5.58E+04	1.16E+00	2.33E-01	-2.10E+00	down
LysoPE 18:0	C23H48NO7P	Lipids	LPE	7.72E+03	1.11E+04	6.19E+03	2.26E+03	2.55E+03	2.59E+03	1.15E+00	2.96E-01	-1.76E+00	down
LysoPC 15:0(2n isomer)	C23H48NO7P	Lipids	LPC	4.95E+04	9.60E+04	1.24E+05	1.41E+04	1.73E+04	1.36E+04	1.14E+00	1.67E-01	-2.58E+00	down
LysoPC 15:0	C23H48NO7P	Lipids	LPC	3.30E+04	5.67E+04	8.41E+04	9.76E+03	1.02E+04	1.12E+04	1.14E+00	1.79E-01	-2.48E+00	down
LysoPC 16:2(2n isomer)	C24H46NO7P	Lipids	LPC	8.13E+04	1.79E+05	2.34E+05	3.69E+04	4.21E+04	3.89E+04	1.09E+00	2.39E-01	-2.07E+00	down
LysoPC 16:2	C24H46NO7P	Lipids	LPC	5.81E+04	5.42E+04	5.46E+04	2.51E+04	2.82E+04	2.75E+04	1.19E+00	4.84E-01	-1.05E+00	down
LysoPC 16:1	C24H48NO7P	Lipids	LPC	6.60E+05	1.41E+06	1.62E+06	3.18E+05	3.32E+05	3.01E+05	1.10E+00	2.58E-01	-1.96E+00	down
LysoPC 16:1(2n isomer)	C24H48NO7P	Lipids	LPC	6.28E+05	1.46E+06	1.72E+06	3.29E+05	3.39E+05	2.94E+05	1.08E+00	2.52E-01	-1.99E+00	down
LysoPC 16:0(2n isomer)	C24H50NO7P	Lipids	LPC	1.08E+06	1.57E+06	1.68E+06	5.94E+05	6.24E+05	6.06E+05	1.14E+00	4.21E-01	-1.25E+00	down
LysoPC 16:0	C24H50NO7P	Lipids	LPC	1.09E+07	1.11E+07	1.03E+07	7.43E+06	8.15E+06	7.41E+06	1.17E+00	7.12E-01	-4.91E-01	insig
LysoPC 17:2	C25H48NO7P	Lipids	LPC	1.09E+05	2.39E+05	2.83E+05	5.81E+04	6.08E+04	5.05E+04	1.08E+00	2.68E-01	-1.90E+00	down
LysoPE 20:2	C25H48NO7P	Lipids	LPE	3.18E+03	3.68E+03	3.62E+03	2.21E+03	1.85E+03	2.48E+03	1.11E+00	6.25E-01	-6.79E-01	insig
LysoPC 17:1	C25H50NO7P	Lipids	LPC	1.47E+05	2.97E+05	3.46E+05	5.03E+04	5.18E+04	3.52E+04	1.14E+00	1.74E-01	-2.53E+00	down
LysoPC 17:0	C25H52NO7P	Lipids	LPC	2.86E+04	3.71E+04	6.49E+04	1.11E+04	1.20E+04	1.25E+04	1.12E+00	2.73E-01	-1.88E+00	down
2-α-Linolenoyl-glycerol-1-O-glucoside	C27H46O9	Lipids	Glycerol ester	5.37E+04	7.64E+04	8.67E+04	3.67E+04	2.82E+04	1.97E+04	1.08E+00	3.90E-01	-1.36E+00	down
1-α-Linolenoyl-glycerol-3-O-glucoside	C27H46O9	Lipids	Glycerol ester	4.31E+04	2.45E+04	5.84E+04	1.03E+04	1.63E+04	1.90E+04	1.02E+00	3.62E-01	-1.47E+00	down
LysoPC 18:4	C26H46NO7P	Lipids	LPC	3.32E+04	1.26E+05	1.29E+05	6.66E+03	5.32E+03	4.40E+03	1.14E+00	5.69E-02	-4.14E+00	down
2-Linoleoylglycerol-1-O-glucoside	C27H48O9	Lipids	Glycerol ester	3.85E+03	1.09E+04	1.12E+04	9.00E+00	9.00E+00	9.00E+00	1.19E+00	1.04E-03	-9.91E+00	down
1-Linoleoylglycerol-3-O-glucoside	C27H48O9	Lipids	Glycerol ester	4.46E+03	8.69E+03	8.05E+03	9.00E+00	9.00E+00	9.00E+00	1.20E+00	1.27E-03	-9.62E+00	down
LysoPC 18:3	C26H48NO7P	Lipids	LPC	5.57E+06	5.51E+06	4.68E+06	3.06E+06	3.18E+06	3.08E+06	1.17E+00	5.91E-01	-7.59E-01	insig
LysoPC 18:3(2n isomer)	C26H48NO7P	Lipids	LPC	2.66E+07	5.00E+07	5.85E+07	9.41E+06	8.95E+06	8.50E+06	1.15E+00	1.99E-01	-2.33E+00	down
LysoPC 18:2	C26H50NO7P	Lipids	LPC	2.66E+06	2.79E+06	3.15E+06	1.79E+06	1.72E+06	1.79E+06	1.17E+00	6.17E-01	-6.96E-01	insig
LysoPC 18:2(2n isomer)	C26H50NO7P	Lipids	LPC	1.66E+06	3.69E+06	4.59E+06	8.46E+05	7.66E+05	7.66E+05	1.09E+00	2.39E-01	-2.06E+00	down
LysoPC 18:1	C26H52NO7P	Lipids	LPC	6.61E+05	8.12E+05	7.83E+05	3.37E+05	3.93E+05	3.94E+05	1.17E+00	4.98E-01	-1.01E+00	down
LysoPC 18:1(2n isomer)	C26H52NO7P	Lipids	LPC	8.84E+06	1.99E+07	2.71E+07	2.46E+06	2.03E+06	2.23E+06	1.14E+00	1.21E-01	-3.05E+00	down
LysoPC 18:0(2n isomer)	C26H54NO7P	Lipids	LPC	3.11E+04	4.10E+04	4.47E+04	2.08E+04	2.79E+04	2.84E+04	9.83E-01	6.61E-01	-5.98E-01	insig
LysoPC 18:0	C26H54NO7P	Lipids	LPC	3.48E+04	5.38E+04	7.01E+04	2.59E+04	3.35E+04	2.80E+04	9.57E-01	5.50E-01	-8.62E-01	insig

LysoPC 19:2(2n isomer)	C27H52NO7P	Lipids	LPC	7.36E+04	1.10E+05	9.62E+04	1.18E+04	1.41E+04	1.41E+04	1.19E+00	1.43E-01	-2.81E+00	down
LysoPC 19:2	C27H52NO7P	Lipids	LPC	6.86E+03	1.95E+04	2.60E+04	4.13E+03	5.34E+03	5.05E+03	9.79E-01	2.78E-01	-1.85E+00	insig
LysoPC 19:1	C27H54NO7P	Lipids	LPC	3.68E+04	6.49E+04	7.47E+04	3.43E+03	3.97E+03	3.21E+03	1.19E+00	6.02E-02	-4.06E+00	down
LysoPC 19:0	C27H56NO7P	Lipids	LPC	1.66E+04	1.64E+04	1.62E+04	5.39E+03	7.89E+03	8.19E+03	1.15E+00	4.36E-01	-1.20E+00	down
LysoPC 20:3	C28H52NO7P	Lipids	LPC	6.94E+03	1.73E+04	1.82E+04	3.26E+03	2.40E+03	2.41E+03	1.11E+00	1.90E-01	-2.40E+00	down
LysoPC 20:2(2n isomer)	C28H54NO7P	Lipids	LPC	2.05E+04	3.76E+04	5.18E+04	9.75E+03	8.25E+03	9.41E+03	1.11E+00	2.50E-01	-2.00E+00	down
LysoPC 20:2	C28H54NO7P	Lipids	LPC	1.32E+04	4.00E+04	4.49E+04	1.01E+04	9.19E+03	9.04E+03	9.85E-01	2.88E-01	-1.80E+00	insig
LysoPC 20:1	C28H56NO7P	Lipids	LPC	1.10E+04	3.14E+04	3.24E+04	4.03E+03	3.78E+03	5.81E+03	1.09E+00	1.82E-01	-2.46E+00	down
1-(9Z-Octadecenoyl)-2-(9-oxo-nonanoyl)-sn-glycero-3-phosphocholine	C35H66NO9P	Lipids	Glycerol ester	3.43E+04	1.22E+04	1.33E+04	1.30E+05	1.29E+05	1.41E+05	1.14E+00	6.67E+00	2.74E+00	up
1-Linolenoyl-rac-glycerol-diglucoside	C33H56O14	Lipids	Free fatty acids	8.61E+05	1.02E+06	7.98E+05	9.17E+05	9.90E+05	9.33E+05	4.76E-01	1.06E+00	8.55E-02	insig
2-α-Linolenoyl-glycerol-1,3-di-O-glucoside	C33H56O14	Lipids	Glycerol ester	6.82E+04	5.61E+04	6.89E+04	3.12E+04	3.45E+04	2.72E+04	1.16E+00	4.81E-01	-1.06E+00	down
1-α-Linolenoyl-glycerol-2,3-di-O-glucoside	C33H56O14	Lipids	Glycerol ester	4.63E+04	5.85E+04	3.86E+04	4.69E+04	4.30E+04	3.87E+04	4.03E-01	8.96E-01	-1.58E-01	insig
2-Linoleoylglycerol-1,3-di-O-glucoside	C33H58O14	Lipids	Glycerol ester	6.48E+03	7.49E+03	6.13E+03	1.40E+03	1.50E+03	1.24E+03	1.19E+00	2.06E-01	-2.28E+00	down
1-Linoleoylglycerol-2,3-di-O-glucoside	C33H58O14	Lipids	Glycerol ester	3.17E+03	1.72E+03	2.68E+03	9.00E+00	9.00E+00	9.00E+00	1.20E+00	3.57E-03	-8.13E+00	down
1-methylguanidine	C2H7N3	Nucleotides and derivatives	Nucleotides and derivatives	5.01E+03	2.81E+03	3.27E+03	4.83E+03	6.17E+03	4.45E+03	7.93E-01	1.39E+00	4.80E-01	insig
Pyrimidine	C4H4N2	Nucleotides and derivatives	Nucleotides and derivatives	9.00E+00	9.00E+00	9.00E+00	2.91E+04	3.15E+04	2.13E+04	1.20E+00	3.03E+03	1.16E+01	up
Cytosine	C4H5N3O	Nucleotides and derivatives	Nucleotides and derivatives	2.94E+04	3.60E+04	3.60E+04	7.10E+05	4.99E+05	6.45E+05	1.20E+00	1.83E+01	4.19E+00	up
Uracil	C4H4N2O2	Nucleotides and derivatives	Nucleotides and derivatives	1.77E+05	1.26E+05	1.37E+05	2.10E+05	1.95E+05	1.94E+05	1.00E+00	1.36E+00	4.48E-01	insig
5-Methylcytosine	C5H7N3O	Nucleotides and derivatives	Nucleotides and derivatives	6.81E+03	5.45E+03	6.67E+03	7.40E+03	6.44E+03	4.58E+03	1.56E-01	9.73E-01	-3.97E-02	insig
Thymine	C5H6N2O2	Nucleotides and derivatives	Nucleotides and derivatives	2.44E+03	5.02E+03	3.66E+03	3.99E+03	3.41E+03	3.00E+03	7.85E-02	9.35E-01	-9.75E-02	insig
Adenine	C5H5N5	Nucleotides and derivatives	Nucleotides and derivatives	2.72E+06	2.21E+06	9.21E+05	9.38E+06	9.51E+06	7.48E+06	1.11E+00	4.51E+00	2.17E+00	up
Allopurinol	C5H4N4O	Nucleotides and derivatives	Nucleotides and derivatives	1.35E+04	2.08E+04	8.67E+03	3.15E+04	2.32E+04	2.06E+04	8.90E-01	1.75E+00	8.07E-01	insig
Hypoxanthine	C5H4N4O	Nucleotides and derivatives	Nucleotides and derivatives	1.78E+04	2.18E+04	6.22E+03	4.23E+04	3.36E+04	2.73E+04	9.11E-01	2.26E+00	1.17E+00	insig
1-Methyladenine	C6H7N5	Nucleotides and derivatives	Nucleotides and derivatives	3.28E+04	4.25E+04	3.26E+04	4.15E+04	4.17E+04	4.03E+04	7.64E-01	1.15E+00	1.95E-01	insig
Guanine	C5H5N5O	Nucleotides and derivatives	Nucleotides and derivatives	1.13E+06	2.07E+06	7.47E+05	2.45E+05	2.45E+05	1.59E+05	1.12E+00	1.65E-01	-2.60E+00	down
Isoguanine	C5H5N5O	Nucleotides and derivatives	Nucleotides and derivatives	1.46E+05	2.39E+05	9.93E+04	4.54E+04	4.51E+04	3.87E+04	1.11E+00	2.66E-01	-1.91E+00	down
Xanthine	C5H4N4O2	Nucleotides and derivatives	Nucleotides and derivatives	1.68E+04	2.37E+04	1.78E+04	9.00E+00	9.00E+00	9.00E+00	1.20E+00	4.63E-04	-1.11E+01	down
7-Methylguanine	C6H7N5O	Nucleotides and derivatives	Nucleotides and derivatives	5.36E+03	8.75E+03	7.68E+03	9.00E+00	9.00E+00	9.00E+00	1.20E+00	1.24E-03	-9.66E+00	down
6-Methylmercaptopurine	C6H6N4S	Nucleotides and derivatives	Nucleotides and derivatives	3.76E+06	2.75E+06	2.64E+06	8.31E+06	8.36E+06	6.29E+06	1.15E+00	2.51E+00	1.33E+00	up
Isoxanthopterin	C6H5N5O2	Nucleotides and derivatives	Nucleotides and derivatives	1.17E+04	8.42E+03	1.09E+04	1.11E+04	1.36E+04	1.29E+04	7.74E-01	1.22E+00	2.83E-01	insig
2-Deoxyribose-1-phosphate	C5H11O7P	Nucleotides and derivatives	Nucleotides and derivatives	1.83E+05	5.75E+04	9.15E+04	8.20E+05	9.19E+05	4.07E+05	1.10E+00	6.47E+00	2.69E+00	up
2-Deoxyribose-5'-phosphate	C5H11O7P	Nucleotides and derivatives	Nucleotides and derivatives	1.42E+04	7.22E+03	6.14E+03	8.28E+03	9.53E+03	1.15E+04	2.63E-01	1.06E+00	8.98E-02	insig
2'-Deoxycytidine	C9H13N3O4	Nucleotides and derivatives	Nucleotides and derivatives	3.56E+04	3.75E+04	3.35E+04	9.97E+04	4.25E+04	6.57E+04	9.43E-01	1.95E+00	9.64E-01	insig
Thymidine	C10H14N2O5	Nucleotides and derivatives	Nucleotides and derivatives	1.00E+04	1.41E+04	1.03E+04	2.07E+04	1.47E+04	1.71E+04	9.93E-01	1.52E+00	6.07E-01	insig
Cytarabine	C9H13N3O5	Nucleotides and derivatives	Nucleotides and derivatives	2.16E+05	2.74E+05	2.43E+05	2.90E+05	2.89E+05	2.67E+05	8.52E-01	1.15E+00	2.07E-01	insig
Cytidine	C9H13N3O5	Nucleotides and derivatives	Nucleotides and derivatives	1.85E+05	2.25E+05	1.69E+05	3.31E+05	3.10E+05	3.20E+05	1.14E+00	1.66E+00	7.31E-01	insig
β-Pseudouridine	C9H12N2O6	Nucleotides and derivatives	Nucleotides and derivatives	1.27E+04	1.90E+04	1.56E+04	1.92E+04	1.27E+04	1.74E+04	1.57E-01	1.04E+00	6.08E-02	insig
Uridine	C9H12N2O6	Nucleotides and derivatives	Nucleotides and derivatives	2.50E+04	1.99E+04	1.46E+04	9.49E+04	8.34E+04	8.16E+04	1.18E+00	4.37E+00	2.13E+00	up
2'-Deoxyadenosine	C10H13N5O3	Nucleotides and derivatives	Nucleotides and derivatives	1.09E+05	2.49E+05	2.01E+05	3.42E+04	4.48E+04	3.61E+04	1.14E+00	2.06E-01	-2.28E+00	down
2'-Deoxyguanosine	C10H13N5O4	Nucleotides and derivatives	Nucleotides and derivatives	7.26E+04	9.59E+04	8.80E+04	5.82E+04	4.84E+04	4.41E+04	1.10E+00	5.88E-01	-7.67E-01	insig
Adenosine	C10H13N5O4	Nucleotides and derivatives	Nucleotides and derivatives	9.10E+06	1.43E+07	2.35E+07	1.71E+06	2.23E+06	1.73E+06	1.16E+00	1.21E-01	-3.05E+00	down
9-(Arabinosyl)hypoxanthine	C10H12N4O5	Nucleotides and derivatives	Nucleotides and derivatives	1.15E+05	9.69E+04	7.61E+04	4.13E+05	3.34E+05	2.60E+05	1.16E+00	3.50E+00	1.81E+00	up
2-Hydroxyadenosine	C10H13N5O5	Nucleotides and derivatives	Nucleotides and derivatives	9.89E+05	1.39E+06	1.48E+06	2.23E+06	2.00E+06	1.82E+06	1.03E+00	1.57E+00	6.48E-01	insig
Guanosine	C10H13N5O5	Nucleotides and derivatives	Nucleotides and derivatives	6.00E+05	8.65E+05	8.71E+05	1.44E+06	1.24E+06	1.05E+06	1.01E+00	1.60E+00	6.75E-01	insig
Xanthosine	C10H12N4O6	Nucleotides and derivatives	Nucleotides and derivatives	2.79E+05	3.71E+05	3.35E+05	2.90E+05	2.88E+05	2.63E+05	7.75E-01	8.53E-01	-2.29E-01	insig
5-Aminoimidazole ribonucleotide	C8H14N3O7P	Nucleotides and derivatives	Nucleotides and derivatives	1.96E+05	2.60E+05	3.60E+05	2.61E+05	2.80E+05	2.80E+05	1.11E-01	1.01E+00	8.07E-03	insig
5'-Deoxy-5'-(methylthio)adenosine	C11H15N5O3S	Nucleotides and derivatives	Nucleotides and derivatives	8.85E+06	1.04E+07	1.04E+07	7.33E+06	7.51E+06	8.26E+06	1.07E+00	7.80E-01	-3.59E-01	insig
N7-Methylguanosine	C11H15N5O5	Nucleotides and derivatives	Nucleotides and derivatives	1.95E+04	3.12E+04	3.44E+04	2.71E+04	1.85E+04	2.59E+04	4.13E-01	8.41E-01	-2.50E-01	insig
8-Hydroxyguanosine	C10H13N5O6	Nucleotides and derivatives	Nucleotides and derivatives	8.74E+03	2.92E+03	3.42E+03	6.05E+03	4.79E+03	5.25E+03	3.13E-01	1.07E+00	9.45E-02	insig
Cytidine 5'-monophosphate(Cytidylic acid)	C9H14N3O8P	Nucleotides and derivatives	Nucleotides and derivatives	5.23E+03	1.06E+04	2.14E+03	1.25E+04	2.06E+04	6.52E+03	7.41E-01	2.20E+00	1.14E+00	insig
Uridine 5'-monophosphate	C9H13N2O9P	Nucleotides and derivatives	Nucleotides and derivatives	1.59E+04	1.90E+04	1.56E+04	3.19E+04	4.57E+04	3.26E+04	1.13E+00	2.18E+00	1.12E+00	up
Cyclic 3',5'-Adenylic acid	C10H12N5O6P	Nucleotides and derivatives	Nucleotides and derivatives	1.03E+04	2.38E+04	8.99E+03	1.03E+04	1.11E+04	1.19E+04	2.97E-01	7.72E-01	-3.73E-01	insig
2'-Deoxyadenosine-5'-monophosphate	C10H14N5O6P	Nucleotides and derivatives	Nucleotides and derivatives	3.64E+05	2.84E+05	3.19E+05	1.29E+05	1.46E+05	1.18E+05	1.18E+00	4.06E-01	-1.30E+00	down
2'-Deoxyinosine-5'-monophosphate	C10H13N4O7P	Nucleotides and derivatives	Nucleotides and derivatives	1.12E+07	1.35E+07	1.32E+07	1.06E+07	1.11E+07	8.71E+06	9.18E-01	8.02E-01	-3.18E-01	insig
β-Nicotinamide mononucleotide	C11H15N2O8P	Nucleotides and derivatives	Nucleotides and derivatives	2.93E+04	2.23E+04	2.51E+04	8.49E+04	1.19E+05	6.96E+04	1.15E+00	3.56E+00	1.83E+00	up
Guanosine 3',5'-cyclic monophosphate	C10H12N5O7P	Nucleotides and derivatives	Nucleotides and derivatives	1.48E+05	2.41E+05	1.88E+05	2.69E+05	2.69E+05	2.55E+05	9.20E-01	1.38E+00	4.60E-01	insig
Adenosine 5'-monophosphate	C10H14N5O7P	Nucleotides and derivatives	Nucleotides and derivatives	6.00E+05	6.93E+05	9.22E+05	3.22E+05	4.50E+05	2.35E+05	1.05E+00	4.55E-01	-1.14E+00	down
Adenosine 2'-Phosphate	C10H14N5O7P	Nucleotides and derivatives	Nucleotides and derivatives	1.21E+04	1.00E+04	9.21E+03	9.58E+04	1.32E+05	6.74E+04	1.18E+00	9.40E+00	3.23E+00	up
Guanosine 5'-monophosphate	C10H14N5O8P	Nucleotides and derivatives	Nucleotides and derivatives	9.02E+03	9.18E+03	1.25E+04	4.96E+03	6.42E+03	2.54E+03	9.90E-01	4.54E-01	-1.14E+00	insig
Isopentenyladenine-7-N-glucoside	C16H23N5O5	Nucleotides and derivatives	Nucleotides and derivatives	5.31E+04	3.73E+04	3.74E+04	4.73E+04	6.56E+04	4.10E+04	5.25E-01	1.20E+00	2.67E-01	insig
Uridine 5'-diphosphate	C9H14N2O12P2	Nucleotides and derivatives	Nucleotides and derivatives	2.97E+05	3.02E+05	2.96E+05	3.22E+05	2.88E+05	2.92E+05	1.23E-01	1.01E+00	1.08E-02	insig
Adenosine 5'-diphosphate	C10H15N5O10P2	Nucleotides and derivatives	Nucleotides and derivatives	1.32E+04	9.28E+03	2.00E+04	2.12E+04	2.06E+04	1.66E+04	7.27E-01	1.38E+00	4.62E-01	insig
Uridine-5'-Diphosphate-D-Xylose	C14H22N2O16P2	Nucleotides and derivatives	Nucleotides and derivatives	4.81E+04	3.63E+04	4.05E+04	1.13E+05	1.08E+05	8.16E+04	1.15E+00	2.42E+00	1.28E+00	up
Uridine 5'-diphospho-D-glucose	C15H24N2O17P2	Nucleotides and derivatives	Nucleotides and derivatives	4.24E+05	2.83E+05	3.61E+05	9.60E+05	1.03E+06	8.20E+05	1.16E+00	2.63E+00	1.40E+00	up
Nicotinic acid adenine dinucleotide	C21H27N7O14P2	Nucleotides and derivatives	Nucleotides and derivatives	1.71E+05	2.07E+05	1.87E+05	3.21E+05	3.40E+05	2.45E+05	1.08E+00	1.60E+00	6.81E-01	insig
NADP	C21H28N7O17P3	Nucleotides and derivatives	Nucleotides and derivatives	4.28E+04	3.75E+04	4.48E+04	5.83E+04	7.08E+04	5.09E+04	1.02E+00	1.44E+00	5.25E-01	insig
3-Hydroxypropanoic acid	C3H6O3	Organic acids	Organic acids	6.92E+03	1.38E+04	1.21E+04	1.31E+04	1.96E+04	1.14E+04	5.99E-01	1.34E+00	4.27E-01	insig
Succinic anhydride	C4H4O3	Organic acids	Organic acids	1046000	1299000	1309400	624710	672600	682690	1.164656512	0.541812609	-0.88413413	insig
Succinic semialdehyde	C4H6O3	Organic acids	Organic acids	4.21E+04	3.94E+04	3.39E+04	1.39E+04	1.35E+04	2.24E+04	1.11E+00	4.32E-01	-1.21E+00	down
γ-Aminobutyric acid	C4H9NO2	Organic acids	Organic acids	1.03E+06	5.61E+05	7.63E+05	4.10E+05	3.75E+05	3.04E+05	1.06E+00	4.64E-01	-1.11E+00	down
Tartronate semialdehyde	C3H4O4	Organic acids	Organic acids	8.90E+05	1.03E+06	1.43E+06	6.02E+05	5.93E+05	6.09E+05	1.09E+00	5.39E-01	-8.91E-01	insig
Hydroxypyruvic acid	C3H4O4	Organic acids	Organic acids	9.82E+05	1.26E+06	1.64E+06	7.64E+05	6.41E+05	6.37E+05	1.07E+00	5.26E-01	-9.27E-01	insig
3-Hydroxybutyric acid	C4H8O3	Organic acids	Organic acids	2.39E+06	1.79E+06	2.69E+06	2.31E+06	2.17E+06	1.70E+06	3.77E-01	9.01E-01	-1.51E-01	insig
Malonic acid	C3H4O4	Organic acids	Organic acids	3.34E+06	2.27E+06	3.35E+06	2.82E+06	2.91E+06	2.17E+06	4.25E-01	8.82E-01	-1.82E-01	insig
Fumaric acid	C4H4O4	Organic acids	Organic acids	3.05E+05	1.77E+05	1.89E+05	9.62E+04	9.45E+04	5.92E+04	1.08E+00	3.72E-01	-1.42E+00	down
3-Methyl-2-Oxobutanoic acid	C5H8O3	Organic acids	Organic acids	1.07E+05	7.29E+04	1.09E+05	1.33E+06	1.11E+06	1.31E+06	1.19E+00	1.30E+01	3.70E+00	up
2-Oxovaleric acid	C5H8O3	Organic acids	Organic acids	9.00E+00	9.00E+00	9.00E+00	9.39E+04	9.09E+04	9.02E+04	1.20E+00	1.02E+04	1.33E+01	up
4-Oxopentanoic Acid	C5H8O3	Organic acids	Organic acids	1.41E+04	1.77E+04	2.13E+04	2.44E+05	1.74E+05	2.31E+05	1.19E+00	1.22E+01	3.61E+00	up
D-Erythronolactone	C4H6O4	Organic acids	Organic acids	4.86E+05	6.14E+05	6.75E+05	6.64E+05	6.10E+05	7.25E+05	5.95E-01	1.13E+00	1.71E-01	insig

Succinic acid	C4H6O4	Organic acids	Organic acids	4.40E+06	4.58E+06	4.30E+06	2.58E+06	2.67E+06	2.53E+06	1.20E+00	5.86E-01	-7.72E-01	insig
Methylmalonic acid	C4H6O4	Organic acids	Organic acids	3.66E+06	3.19E+06	3.47E+06	2.22E+06	2.36E+06	2.13E+06	1.17E+00	6.51E-01	-6.20E-01	insig
Acetoxyacetic acid	C4H6O4	Organic acids	Organic acids	1.65E+04	1.52E+04	1.51E+04	9.00E+00	9.00E+00	9.00E+00	1.20E+00	5.77E-04	-1.08E+01	down
β-Hydroxyisovaleric acid	C5H10O3	Organic acids	Organic acids	5.74E+04	3.13E+06	3.38E+06	5.60E+04	6.82E+04	7.71E+04	8.26E-01	3.07E-02	-5.03E+00	insig
Aminomalonic acid	C3H5NO4	Organic acids	Organic acids	3.82E+06	5.00E+06	4.68E+06	2.16E+06	2.25E+06	2.40E+06	1.16E+00	5.05E-01	-9.87E-01	insig
L-Homoserine	C4H9NO3	Organic acids	Organic acids	2.30E+05	1.26E+05	1.36E+05	3.13E+05	3.49E+05	2.31E+05	9.69E-01	1.81E+00	8.58E-01	insig
Isonicotinic acid	C6H5NO2	Organic acids	Organic acids	3.86E+05	3.45E+05	4.23E+05	2.56E+05	2.74E+05	2.05E+05	1.09E+00	6.37E-01	-6.50E-01	insig
2-Picolinic acid	C6H5NO2	Organic acids	Organic acids	2.72E+05	2.13E+05	3.16E+05	1.68E+05	1.69E+05	1.46E+05	1.08E+00	6.02E-01	-7.31E-01	insig
1-Pyrroline-4-hydroxy-2-carboxylic acid	C5H7NO3	Organic acids	Organic acids	3.64E+03	1.59E+03	3.26E+03	1.11E+04	5.32E+03	9.96E+03	1.03E+00	3.11E+00	1.64E+00	up
Citraconic acid	C5H6O4	Organic acids	Organic acids	7.48E+04	6.68E+04	7.63E+04	1.09E+05	1.06E+05	1.17E+05	1.17E+00	1.52E+00	6.08E-01	insig
Methylenesuccinic acid	C5H6O4	Organic acids	Organic acids	2.69E+04	2.11E+04	2.50E+04	2.89E+04	3.13E+04	1.91E+04	2.19E-01	1.08E+00	1.18E-01	insig
3-Guanidinopropionic acid	C4H9N3O2	Organic acids	Organic acids	2.14E+04	1.44E+04	2.69E+04	3.79E+04	3.85E+04	4.72E+04	1.05E+00	1.97E+00	9.77E-01	insig
6-Aminocaproic acid	C6H13NO2	Organic acids	Organic acids	6.78E+05	5.83E+05	5.78E+05	8.51E+05	7.22E+05	6.91E+05	9.49E-01	1.23E+00	3.00E-01	insig
Glutaric acid	C5H8O4	Organic acids	Organic acids	5.78E+05	6.35E+05	7.42E+05	7.25E+05	4.82E+05	8.03E+05	4.21E-02	1.03E+00	4.02E-02	insig
4-Hydroxy-2-Oxopentanoic Acid	C5H8O4	Organic acids	Organic acids	4.40E+04	3.73E+04	4.35E+04	1.89E+04	1.71E+04	1.75E+04	1.19E+00	4.29E-01	-1.22E+00	down
Dimethylmalonic acid	C5H8O4	Organic acids	Organic acids	6.51E+04	8.11E+04	7.82E+04	8.13E+04	5.33E+04	8.69E+04	9.80E-02	9.87E-01	-1.87E-02	insig
2-Methylsuccinic acid	C5H8O4	Organic acids	Organic acids	7.44E+05	8.17E+05	9.65E+05	9.58E+05	6.45E+05	1.03E+06	1.08E-01	1.04E+00	6.07E-02	insig
2-Hydroxy-2-methyl-3-oxobutanoic acid	C5H8O4	Organic acids	Organic acids	3.11E+04	3.12E+04	3.19E+04	1.46E+04	1.33E+04	1.95E+04	1.14E+00	5.03E-01	-9.92E-01	insig
3-Ureidopropionic Acid	C4H8N2O3	Organic acids	Organic acids	4.18E+03	2.60E+03	2.92E+03	3.06E+04	2.06E+04	2.08E+04	1.18E+00	7.43E+00	2.89E+00	up
2-Hydroxyisocaproic acid	C6H12O3	Organic acids	Organic acids	4.23E+04	6.51E+04	5.65E+04	1.01E+05	8.02E+04	9.68E+04	1.06E+00	1.70E+00	7.63E-01	insig
6-Hydroxyhexanoic acid	C6H12O3	Organic acids	Organic acids	3.70E+04	3.46E+04	2.17E+04	3.07E+04	5.93E+04	3.62E+04	5.79E-01	1.35E+00	4.36E-01	insig
2-Hydroxy-4-methylpentanoic acid	C6H12O3	Organic acids	Organic acids	2.36E+05	2.23E+05	2.79E+05	4.45E+05	5.08E+05	4.47E+05	1.16E+00	1.90E+00	9.26E-01	insig
3-Dehydro-L-Threonic Acid	C4H6O5	Organic acids	Organic acids	6.63E+07	3.47E+07	4.67E+07	3.00E+07	2.98E+07	2.53E+07	9.62E-01	5.76E-01	-7.96E-01	insig
L-Malic acid	C4H6O5	Organic acids	Organic acids	3.23E+06	1.59E+06	2.18E+06	1.17E+06	1.24E+06	1.06E+06	1.02E+00	4.95E-01	-1.02E+00	down
2,3-Dihydroxy-3-Methylbutanoic Acid	C5H10O4	Organic acids	Organic acids	1.37E+07	7.50E+06	8.40E+06	6.30E+06	6.71E+06	4.43E+06	9.00E-01	5.90E-01	-7.62E-01	insig
3-Hydroxybenzoic Acid	C7H6O3	Organic acids	Organic acids	4.69E+06	1.02E+07	6.12E+06	6.64E+05	5.69E+05	7.26E+05	1.17E+00	9.30E-02	-3.43E+00	down
Urocanic acid	C6H6N2O2	Organic acids	Organic acids	9.00E+00	9.00E+00	9.00E+00	1.40E+04	4.08E+03	1.72E+04	1.19E+00	1.31E+03	1.03E+01	up
Muconic acid	C6H6O4	Organic acids	Organic acids	1.31E+06	1.24E+06	1.38E+06	1.07E+06	1.28E+06	1.36E+06	4.53E-01	9.45E-01	-8.17E-02	insig
2-n-Propyl-3-pentenoic acid	C8H14O2	Organic acids	Organic acids	2.98E+05	2.05E+05	2.66E+05	1.71E+05	1.28E+05	1.74E+05	1.02E+00	6.16E-01	-7.00E-01	insig
4-Acetamidobutyric acid	C6H11NO3	Organic acids	Organic acids	4.77E+05	6.21E+05	5.56E+05	1.74E+05	1.95E+05	1.67E+05	1.19E+00	3.24E-01	-1.63E+00	down
4-Guanidinobutyric acid	C5H11N3O2	Organic acids	Organic acids	1.14E+06	1.98E+06	1.25E+06	3.08E+06	2.78E+06	3.35E+06	1.09E+00	2.11E+00	1.08E+00	up
α-Ketoglutaric acid	C5H6O5	Organic acids	Organic acids	1.21E+06	6.93E+05	8.17E+05	7.78E+05	7.91E+05	7.41E+05	4.55E-01	8.48E-01	-2.38E-01	insig
2-Methyl-3-oxosuccinic acid	C5H6O5	Organic acids	Organic acids	3.36E+05	2.98E+05	3.26E+05	1.50E+05	1.24E+05	1.52E+05	1.18E+00	4.42E-01	-1.18E+00	down
2-Methylglutaric acid	C6H10O4	Organic acids	Organic acids	5.75E+05	8.06E+05	1.25E+06	3.03E+06	2.31E+06	2.96E+06	1.11E+00	3.15E+00	1.66E+00	up
Adipic Acid	C6H10O4	Organic acids	Organic acids	6.80E+05	8.99E+05	1.50E+06	3.50E+06	2.86E+06	3.64E+06	1.11E+00	3.25E+00	1.70E+00	up
2-Acetyl-2-Hydroxybutanoic Acid	C6H10O4	Organic acids	Organic acids	9.74E+04	1.70E+05	2.28E+05	5.85E+05	4.21E+05	5.75E+05	1.10E+00	3.19E+00	1.67E+00	up
L-Citramalic acid	C5H8O5	Organic acids	Organic acids	7.09E+05	8.36E+05	7.57E+05	3.57E+05	3.16E+05	3.39E+05	1.19E+00	4.40E-01	-1.19E+00	down
3-Methylmalic acid	C5H8O5	Organic acids	Organic acids	8.73E+04	1.25E+05	1.21E+05	8.94E+04	7.06E+04	7.35E+04	9.43E-01	7.00E-01	-5.14E-01	insig
2-Hydroxyglutaric Acid	C5H8O5	Organic acids	Organic acids	1.01E+05	1.34E+05	1.36E+05	9.12E+04	1.20E+05	1.26E+05	3.92E-01	9.11E-01	-1.35E-01	insig
Mevalonic acid	C6H12O4	Organic acids	Organic acids	3.59E+04	1.92E+04	2.63E+04	4.23E+04	6.85E+04	3.97E+04	9.25E-01	1.85E+00	8.86E-01	insig
L-Tartaric acid	C4H6O6	Organic acids	Organic acids	1.24E+05	1.61E+05	1.39E+05	1.64E+05	1.46E+05	2.17E+05	7.21E-01	1.24E+00	3.15E-01	insig
2-Hydroxyphenylacetic acid	C8H8O3	Organic acids	Organic acids	5.45E+04	3.34E+04	3.78E+04	3.94E+03	2.63E+03	4.43E+03	1.18E+00	8.75E-02	-3.52E+00	down
3-Hydroxyanthranilic acid	C7H7NO3	Organic acids	Organic acids	6.01E+03	2.34E+04	8.82E+03	3.17E+04	2.89E+04	2.69E+04	9.34E-01	2.29E+00	1.19E+00	insig
2,3-Dihydroxybenzoic Acid	C7H6O4	Organic acids	Organic acids	1.49E+06	1.92E+06	1.66E+06	1.54E+06	1.17E+06	1.71E+06	5.60E-01	8.70E-01	-2.00E-01	insig
Diethyl phosphate	C4H11O4P	Organic acids	Organic acids	1.22E+04	1.04E+04	1.39E+04	9.52E+03	1.30E+04	1.78E+04	2.08E-01	1.11E+00	1.44E-01	insig
5-Acetamidopentanoic Acid	C7H13NO3	Organic acids	Organic acids	7.20E+04	5.34E+04	6.99E+04	7.59E+04	7.70E+04	6.97E+04	6.78E-01	1.14E+00	1.89E-01	insig
2-Oxoadipic acid	C6H8O5	Organic acids	Organic acids	7.63E+04	9.96E+04	1.02E+05	7.36E+04	7.67E+04	7.37E+04	8.93E-01	8.06E-01	-3.11E-01	insig
2-Propylsuccinic acid	C7H12O4	Organic acids	Organic acids	7.80E+05	1.09E+06	9.68E+05	3.36E+05	3.55E+05	3.05E+05	1.18E+00	3.51E-01	-1.51E+00	down
Pimelic acid	C7H12O4	Organic acids	Organic acids	7.17E+05	1.06E+06	9.15E+05	2.87E+05	3.20E+05	2.78E+05	1.17E+00	3.29E-01	-1.60E+00	down
Phenylpyruvic acid	C9H8O3	Organic acids	Organic acids	5.12E+05	7.51E+05	5.49E+05	3.86E+05	3.69E+05	3.46E+05	1.07E+00	6.08E-01	-7.19E-01	insig
2-Hydroxycinnamic acid	C9H8O3	Organic acids	Organic acids	1.61E+05	1.34E+05	1.42E+05	2.66E+05	2.61E+05	2.84E+05	1.18E+00	1.86E+00	8.93E-01	insig
Phthalic acid	C8H6O4	Organic acids	Organic acids	1.02E+05	9.26E+04	8.88E+04	9.64E+04	1.03E+05	9.12E+04	2.70E-01	1.03E+00	3.61E-02	insig
D-Xylonic acid	C5H10O6	Organic acids	Organic acids	1.17E+06	1.11E+06	1.28E+06	2.15E+06	1.84E+06	2.13E+06	1.17E+00	1.72E+00	7.82E-01	insig
2-Hydroxy-3-phenylpropanoic acid	C9H10O3	Organic acids	Organic acids	1.06E+04	1.39E+04	7.90E+03	8.90E+04	6.41E+04	9.19E+04	1.18E+00	7.56E+00	2.92E+00	up
3-(3-Hydroxyphenyl)-propionic acid	C9H10O3	Organic acids	Organic acids	2.13E+05	1.31E+05	1.44E+05	9.00E+00	9.00E+00	9.00E+00	1.20E+00	5.54E-05	-1.41E+01	down
Dihydrojasmone	C11H18O	Organic acids	Organic acids	2.75E+04	1.58E+04	1.94E+04	5.67E+03	1.23E+04	1.07E+04	9.87E-01	4.57E-01	-1.13E+00	insig
Phosphoenolpyruvate	C3H5O6P	Organic acids	Organic acids	1.35E+05	1.02E+05	1.05E+05	1.74E+05	2.77E+05	1.52E+05	9.54E-01	1.76E+00	8.17E-01	insig
DL-Glyceraldehyde-3-phosphate	C3H7O6P	Organic acids	Organic acids	6.24E+04	4.02E+04	3.33E+04	1.97E+04	4.20E+04	4.38E+04	4.88E-01	7.77E-01	-3.64E-01	insig
3-Dehydroshikimic acid	C7H8O5	Organic acids	Organic acids	9.34E+04	1.58E+05	1.03E+05	1.37E+05	8.19E+04	1.04E+05	2.21E-01	9.11E-01	-1.35E-01	insig
9-Oxononanoic acid	C9H16O3	Organic acids	Organic acids	9.37E+03	7.07E+03	1.17E+04	1.73E+04	1.35E+04	2.57E+04	9.82E-01	2.01E+00	1.00E+00	insig
Decanoic acid	C10H20O2	Organic acids	Organic acids	9.51E+02	1.55E+03	1.50E+03	1.44E+03	1.80E+03	1.91E+03	7.17E-01	1.29E+00	3.66E-01	insig
Cis-Aconitic acid	C6H6O6	Organic acids	Organic acids	2.69E+04	8.50E+04	6.59E+04	1.14E+05	6.99E+04	1.24E+05	7.55E-01	1.73E+00	7.93E-01	insig
Trans-Citridic acid	C6H6O6	Organic acids	Organic acids	2.11E+06	1.47E+06	1.60E+06	1.61E+06	1.73E+06	1.60E+06	1.82E-01	9.56E-01	-6.48E-02	insig
Shikimic acid	C7H10O5	Organic acids	Organic acids	1.23E+05	3.24E+05	2.58E+05	4.63E+05	2.42E+05	4.16E+05	7.04E-01	1.59E+00	6.71E-01	insig
Suberic Acid	C8H14O4	Organic acids	Organic acids	2.33E+06	1.27E+06	1.58E+06	3.05E+06	2.86E+06	2.47E+06	9.66E-01	1.62E+00	6.93E-01	insig
2-Propylglutaric acid	C8H14O4	Organic acids	Organic acids	7.78E+06	7.72E+06	8.10E+06	1.38E+07	1.57E+07	1.50E+07	1.19E+00	1.88E+00	9.14E-01	insig
2-Propylmalic Acid	C7H12O5	Organic acids	Organic acids	8.25E+05	1.07E+06	1.40E+06	3.80E+05	4.10E+05	3.57E+05	1.15E+00	3.48E-01	-1.52E+00	down
3-Isopropylmalic Acid	C7H12O5	Organic acids	Organic acids	8.14E+05	1.12E+06	1.40E+06	4.47E+05	4.09E+05	3.95E+05	1.14E+00	3.75E-01	-1.41E+00	down
2-Isopropylmalic Acid	C7H12O5	Organic acids	Organic acids	6.91E+05	1.12E+06	1.31E+06	4.17E+05	3.97E+05	3.90E+05	1.11E+00	3.86E-01	-1.37E+00	down
4,5,6-Trihydroxy-2-oxohexanoic acid	C6H10O6	Organic acids	Organic acids	6.68E+04	5.88E+04	4.81E+04	3.36E+04	2.61E+04	3.41E+04	1.10E+00	5.40E-01	-8.89E-01	insig
Azelaic acid	C9H16O4	Organic acids	Organic acids	7.89E+05	7.86E+05	6.96E+05	8.44E+05	8.25E+05	7.74E+05	7.39E-01	1.08E+00	1.05E-01	insig
Citric Acid	C6H8O7	Organic acids	Organic acids	1.16E+07	8.83E+06	1.06E+07	1.84E+07	1.75E+07	1.84E+07	1.15E+00	1.75E+00	8.07E-01	insig
Isocitric Acid	C6H8O7	Organic acids	Organic acids	9.09E+06	9.76E+06	1.01E+07	1.73E+07	1.87E+07	1.89E+07	1.19E+00	1.90E+00	9.25E-01	insig
Quinic Acid	C7H12O6	Organic acids	Organic acids	3.04E+06	3.28E+06	3.14E+06	6.02E+06	6.44E+06	6.31E+06	1.20E+00	1.99E+00	9.89E-01	insig
D-Galacturonic acid	C6H10O7	Organic acids	Organic acids	1.77E+05	2.12E+05	2.34E+05	9.95E+04	9.67E+04	9.90E+04	1.17E+00	4.73E-01	-1.08E+00	down
3,4-Dimethoxyphenyl acetic acid	C10H12O4	Organic acids	Organic acids	2.11E+04	9.57E+03	1.71E+04	1.62E+04	1.30E+04	6.66E+03	4.64E-01	7.52E-01	-4.11E-01	insig
Sebacate	C10H18O4	Organic acids	Organic acids	6.93E+03	6.31E+03	1.45E+03	9.93E+03	8.52E+03	7.01E+03	7.13E-01	1.73E+00	7.93E-01	insig
4,8-Dihydroxyquinoline-2-carboxylic acid	C10H7NO4	Organic acids	Organic acids	2.42E+05	2.10E+05	1.91E+05	1.20E+05	1.61E+05	1.14E+05	1.07E+00	6.12E-01	-7.08E-01	insig
Indole-3-lactic acid	C11H11NO3	Organic acids	Organic acids	2.89E+03	9.00E+00	9.00E+00	1.21E+04	1.18E+04	9.95E+03	9.67E-01	1.17E+01	3.54E+00	insig
Jasmonic acid	C12H18O3	Organic acids	Organic acids	1.18E+06	1.88E+06	1.56E+06	6.66E+05	5.09E+05	5.07E+05	1.14E+00	3.64E-01	-1.46E+00	down
Dihydrojasmonic acid	C12H20O3	Organic acids	Organic acids	9.00E+00	9.00E+00	9.00E+00	5.89E+04	5.74E+04	6.60E+04	1.20E+00	6.75E+03	1.27E+01	up
Methyl jasmonate	C13H20O3	Organic acids	Organic acids	6.10E+05	1.35E+06	1.75E+06	3.37E+05	2.57E+05	2.97E+05	1.08E+00	2.40E-01	-2.06E+00	down
Dodecanedioic aicd	C12H22O4	Organic acids	Organic acids	1.61E+03	1.08E+03	6.05E+02	1.73E+03	1.59E+03	1.07E+03	5.74E-01	1.33E+00	4.12E-01	insig
2-Hydroxyhexadecanoic acid	C16H32O3	Organic acids	Organic acids	5.82E+05	7.61E+05	7.83E+05	2.04E+06	2.11E+06	2.16E+06	1.18E+00	2.97E+00	1.57E+00	up
Argininosuccinic acid	C10H18N4O6	Organic acids	Organic acids	1.03E+05	1.45E+05	1.71E+05	3.63E+04	4.03E+04	3.98E+04	1.17E+00	2.78E-01	-1.85E+00	down
(-)-Jasmonoyl-L-Isoleucine	C18H29NO4	Organic acids	Organic acids	3.53E+04	9.11E+04	2.49E+04	1.72E+05	1.28E+05	1.13E+05	9.88E-01	2.73E+00	1.45E+00	insig
Styrene	C8H8	Others	Others	3.88E+04	7.81E+04	7.90E+04	9.00E+00	9.00E+00	9.00E+00	1.20E+00	1.38E-04	-1.28E+01	down
D-(-)-Threose	C4H8O4	Others	Saccharides and Alcohols	2.24E+04	3.68E+04	1.78E+04	7.92E+04	8.14E+04	1.03E+05	1.13E+00	3.42E+00	1.77E+00	up
(S)-2-Phenyloxirane	C8H8O	Others	Others	1.00E+06	1.37E+06	6.39E+05	3.60E+06	3.68E+06	4.80E+06	1.14E+00	4.01E+00	2.00E+00	up
3-Methylbenzaldehyde	C8H8O	Others	Others	1.47E+04	1.19E+04	1.08E+04	8.46E+03	8.69E+03	8.22E+03	1.08E+00	6.78E-01	-5.60E-01	insig
Nicotinamide	C6H6N2O	Others	Vitamin	6.79E+05	8.73E+05	7.34E+05	5.69E+05	4.68E+05	5.66E+05	1.04E+00	7.01E-01	-5.12E-01	insig
D-Threitol	C4H10O4	Others	Saccharides and Alcohols	7.07E+05	6.15E+05	6.67E+05	2.69E+05	1.42E+05	3.60E+05	1.05E+00	3.87E-01	-1.37E+00	down
Nicotinic acid	C6H5NO2	Others	Vitamin	1.66E+05	1.29E+05	1.68E+05	1.23E+05	1.24E+05	6.71E+04	8.30E-01	6.78E-01	-5.60E-01	insig
D-Threonic Acid	C4H8O5	Others	Saccharides and Alcohols	7.13E+05	8.07E+05	8.83E+05	5.48E+05	4.41E+05	6.15E+05	1.04E+00	6.67E-01	-5.84E-01	insig
D-Arabinono-1,4-lactone	C5H8O5	Others	Saccharides and Alcohols	4.33E+05	5.07E+05	5.33E+05	2.32E+05	2.69E+05	2.54E+05	1.17E+00	5.14E-01	-9.61E-01	insig
D-Arabinose	C5H10O5	Others	Saccharides and Alcohols	2.78E+05	3.73E+05	3.09E+05	3.03E+05	2.15E+05	3.44E+05	3.99E-01	8.97E-01	-1.57E-01	insig
D-Ribose	C5H10O5	Others	Saccharides and Alcohols	1.56E+05	2.44E+05	2.09E+05	2.09E+05	1.75E+05	2.77E+05	2.60E-01	1.09E+00	1.19E-01	insig
D-Arabitol	C5H12O5	Others	Saccharides and Alcohols	3.02E+05	4.21E+05	3.53E+05	1.19E+05	8.71E+04	1.61E+05	1.12E+00	3.41E-01	-1.55E+00	down
Xylitol	C5H12O5	Others	Saccharides and Alcohols	2.30E+05	2.96E+05	2.74E+05	9.51E+04	6.16E+04	1.09E+05	1.14E+00	3.32E-01	-1.59E+00	down
1,5-Anhydro-D-glucitol	C6H12O5	Others	Saccharides and Alcohols	4.14E+03	4.34E+03	3.87E+03	1.30E+04	1.55E+04	2.49E+04	1.16E+00	4.32E+00	2.11E+00	up
Sodium Valproate	C8H15NaO2	Others	Others	1.57E+06	1.38E+06	1.46E+06	2.42E+06	2.78E+06	2.68E+06	1.18E+00	1.79E+00	8.39E-01	insig
Pyridoxal	C8H9NO3	Others	Vitamin	5.05E+05	5.55E+05	5.65E+05	3.70E+05	3.02E+05	4.03E+05	1.10E+00	6.62E-01	-5.96E-01	insig
Griffonilide	C8H8O4	Others	Others	2.64E+04	3.27E+04	2.97E+04	2.14E+04	2.09E+04	1.76E+04	1.10E+00	6.75E-01	-5.66E-01	insig
Pyridoxine	C8H11NO3	Others	Vitamin	4.39E+05	5.19E+05	4.24E+05	9.90E+04	8.41E+04	8.89E+04	1.19E+00	1.97E-01	-2.35E+00	down
Dihydroxyacetone phosphate	C3H7O6P	Others	Saccharides and Alcohols	3.16E+04	3.34E+04	1.91E+04	3.63E+04	2.57E+04	2.85E+04	2.78E-01	1.07E+00	1.04E-01	insig
1,10-Decanediol	C10H22O2	Others	Saccharides and Alcohols	5.55E+04	5.40E+04	6.62E+04	9.00E+00	9.00E+00	9.00E+00	1.20E+00	1.54E-04	-1.27E+01	down
3,7-Dihydroxychromen-4-one	C9H6O4	Others	Others	1.12E+05	5.02E+04	6.52E+04	9.00E+00	9.00E+00	9.00E+00	1.20E+00	1.19E-04	-1.30E+01	down
D-Glucono-1,5-lactone	C6H10O6	Others	Saccharides and Alcohols	1.06E+04	1.49E+04	1.77E+04	3.53E+03	9.29E+03	1.08E+04	8.15E-01	5.46E-01	-8.72E-01	insig
D-Glucosamine	C6H13NO5	Others	Saccharides and Alcohols	4.26E+04	8.20E+04	5.64E+04	8.15E+04	4.50E+04	7.78E+04	2.82E-01	1.13E+00	1.74E-01	insig
D-Glucose	C6H12O6	Others	Saccharides and Alcohols	1.14E+06	2.71E+06	2.25E+06	5.38E+05	3.98E+05	6.29E+05	1.09E+00	2.57E-01	-1.96E+00	down
D-Fructose	C6H12O6	Others	Saccharides and Alcohols	1.21E+06	2.69E+06	3.11E+06	4.92E+05	3.09E+05	6.62E+05	1.08E+00	2.09E-01	-2.26E+00	down
Inositol	C6H12O6	Others	Saccharides and Alcohols	1.26E+06	3.30E+06	3.11E+06	5.44E+05	3.39E+05	7.69E+05	1.06E+00	2.15E-01	-2.22E+00	down
Dihydroactinidiolide	C11H16O2	Others	Others	9.00E+05	7.59E+05	7.63E+05	8.55E+05	8.67E+05	7.35E+05	1.16E-01	1.01E+00	2.08E-02	insig
D-Sorbitol	C6H14O6	Others	Saccharides and Alcohols	2.20E+05	2.25E+05	2.22E+05	1.45E+05	1.11E+05	1.89E+05	9.69E-01	6.68E-01	-5.81E-01	insig

D-Mannitol	C6H14O6	Others	Saccharides and Alcohols	7.05E+04	6.88E+04	7.98E+04	4.22E+04	4.26E+04	6.51E+04	9.62E-01	6.85E-01	-5.46E-01	insig
Dulcitol	C6H14O6	Others	Saccharides and Alcohols	3.03E+05	3.19E+05	3.32E+05	1.94E+05	1.75E+05	3.24E+05	8.17E-01	7.26E-01	-4.61E-01	insig
4-Pyridoxic acid	C8H9NO4	Others	Vitamin	6.32E+04	6.35E+04	7.10E+04	1.25E+04	1.40E+04	1.43E+04	1.20E+00	2.06E-01	-2.28E+00	down
3-Phospho-D-glyceric acid	C3H7O7P	Others	Saccharides and Alcohols	6.39E+05	4.36E+05	5.45E+05	4.50E+05	4.91E+05	3.72E+05	7.22E-01	8.10E-01	-3.04E-01	insig
D-Glucoronic acid	C6H10O7	Others	Saccharides and Alcohols	1.99E+05	2.59E+05	2.25E+05	4.37E+04	8.24E+04	8.59E+04	1.13E+00	3.10E-01	-1.69E+00	down
D-Pinitol	C7H14O6	Others	Saccharides and Alcohols	9.84E+04	1.24E+05	1.09E+05	1.61E+05	1.19E+05	2.02E+05	8.81E-01	1.46E+00	5.44E-01	insig
Gluconic acid	C6H12O7	Others	Saccharides and Alcohols	5.05E+05	6.36E+05	6.28E+05	6.34E+05	4.92E+05	6.05E+05	1.18E-01	9.78E-01	-3.27E-02	insig
D-Erythrose-4-phosphate	C4H9O7P	Others	Saccharides and Alcohols	5.95E+04	4.72E+04	6.94E+04	7.37E+04	8.74E+04	7.66E+04	9.29E-01	1.35E+00	4.33E-01	insig
Senkyunolide F	C12H14O3	Others	Others	9.00E+00	9.00E+00	9.00E+00	1.54E+04	1.24E+04	9.00E+00	8.50E-01	1.03E+03	1.00E+01	insig
D-Saccharic acid	C6H10O8	Others	Saccharides and Alcohols	7.92E+05	6.05E+05	7.92E+05	3.60E+05	3.65E+05	4.07E+05	1.15E+00	5.17E-01	-9.51E-01	insig
D-Galactaric acid	C6H10O8	Others	Saccharides and Alcohols	6.49E+05	4.71E+05	6.88E+05	3.32E+05	2.82E+05	3.15E+05	1.12E+00	5.14E-01	-9.61E-01	insig
Sedoheptulose	C7H14O7	Others	Saccharides and Alcohols	5.13E+05	6.99E+05	7.97E+05	3.74E+05	2.40E+05	4.16E+05	1.01E+00	5.13E-01	-9.63E-01	insig
Dihydroylindesteolene	C15H16O	Others	Others	9.39E+05	8.48E+05	6.61E+04	9.00E+00	9.00E+00	9.00E+00	1.18E+00	1.46E-05	-1.61E+01	down
D-Pantothenic Acid	C9H17NO5	Others	Vitamin	7.75E+05	6.83E+05	7.38E+05	6.08E+05	6.73E+05	6.08E+05	1.01E+00	8.60E-01	-2.17E-01	insig
N-Acetyl-D-mannosamine	C8H15NO6	Others	Saccharides and Alcohols	2.14E+04	1.51E+04	1.20E+04	2.23E+04	3.27E+04	2.14E+04	8.76E-01	1.58E+00	6.56E-01	insig
N-Acetyl-D-glucosamine	C8H15NO6	Others	Saccharides and Alcohols	1.95E+05	1.81E+05	1.64E+05	2.11E+05	2.07E+05	2.36E+05	1.00E+00	1.21E+00	2.74E-01	insig
Solatuberenol A	C13H20O3	Others	Others	1.17E+05	2.66E+05	1.59E+05	4.41E+05	3.92E+05	3.10E+05	1.01E+00	2.11E+00	1.08E+00	up
Annuionone D	C13H20O3	Others	Others	3.55E+05	2.99E+05	2.85E+05	7.48E+05	7.97E+05	6.58E+05	1.18E+00	2.35E+00	1.23E+00	up
Dehydrocamphorlactone	C15H16O2	Others	Others	2.41E+05	2.28E+05	1.74E+05	9.00E+00	9.00E+00	9.00E+00	1.20E+00	4.19E-05	-1.45E+01	down
Biotin	C10H16N2O3S	Others	Vitamin	3.77E+04	4.45E+04	5.69E+04	9.00E+00	9.00E+00	9.00E+00	1.20E+00	1.94E-04	-1.23E+01	down
Hydroxyindesteolide	C15H18O3	Others	Others	4.99E+04	3.78E+04	6.77E+04	9.00E+00	9.00E+00	9.00E+00	1.20E+00	1.74E-04	-1.25E+01	down
Nicotinate D-ribonucleoside	C11H14NO6+	Others	Vitamin	1.91E+06	1.53E+06	1.23E+06	1.41E+06	1.68E+06	1.19E+06	2.95E-01	9.16E-01	-1.27E-01	insig
D-Glucosamine 1-phosphate	C6H14NO8P	Others	Saccharides and Alcohols	2.15E+04	2.88E+04	3.02E+04	3.55E+04	5.33E+04	3.23E+04	8.65E-01	1.50E+00	5.89E-01	insig
Glucose-1-phosphate	C6H13O9P	Others	Saccharides and Alcohols	3.78E+06	3.88E+06	3.70E+06	3.38E+06	3.69E+06	3.03E+06	8.63E-01	8.90E-01	-1.69E-01	insig
D-Glucose 6-phosphate	C6H13O9P	Others	Saccharides and Alcohols	3.75E+06	3.61E+06	3.77E+06	3.64E+06	3.97E+06	3.66E+06	1.95E-01	1.01E+00	1.70E-02	insig
D-Fructose 6-phosphate	C6H13O9P	Others	Saccharides and Alcohols	5.45E+05	7.50E+05	7.14E+05	5.97E+05	9.25E+05	8.01E+05	4.59E-01	1.16E+00	2.09E-01	insig
Sorbitol-6-phosphate	C6H15O9P	Others	Saccharides and Alcohols	1.84E+04	1.73E+04	2.19E+04	3.71E+04	4.13E+04	4.48E+04	1.17E+00	2.14E+00	1.10E+00	up
Senkyunolide M	C16H22O4	Others	Others	4.66E+06	4.63E+06	4.64E+06	5.38E+06	4.97E+06	4.96E+06	1.05E+00	1.10E+00	1.37E-01	insig
Retinol (Vitamin A1)	C20H30O	Others	Vitamin	8.40E+03	2.61E+04	1.65E+04	9.00E+00	9.00E+00	9.00E+00	1.20E+00	5.30E-04	-1.09E+01	down
3'-Deoxysappanol	C16H16O5	Others	Others	1.15E+05	1.67E+05	1.65E+05	1.44E+04	1.86E+04	1.94E+04	1.19E+00	1.17E-01	-3.09E+00	down
Glucarate O-Phosphoric acid	C6H11PO11	Others	Saccharides and Alcohols	1.03E+06	8.35E+05	8.08E+05	1.31E+06	1.03E+06	1.44E+06	9.79E-01	1.42E+00	5.04E-01	insig
D-Sedoheptuiose 7-phosphate	C7H15O10P	Others	Saccharides and Alcohols	7.49E+05	5.54E+05	6.07E+05	1.86E+06	2.02E+06	1.17E+06	1.11E+00	2.65E+00	1.40E+00	up
6-Demethoxy-7-methylcapillarisin	C16H12O6	Others	Others	9.00E+00	9.00E+00	9.00E+00	5.78E+05	5.03E+05	8.38E+05	1.20E+00	7.11E+04	1.61E+01	up
N-Acetyl-D-glucosamine-1-phosphate	C8H16NO9P	Others	Saccharides and Alcohols	1.07E+04	2.03E+04	5.93E+03	1.34E+04	1.79E+04	8.96E+03	2.53E-01	1.09E+00	1.25E-01	insig
3,5,7,4'-Tetrahydroxy-Coumaronochromone	C15H10O7	Others	Others	2.99E+04	2.53E+04	2.14E+04	9.53E+03	1.34E+04	1.57E+04	1.07E+00	5.04E-01	-9.88E-01	insig
Idesin	C13H18O8	Others	Others	8.16E+05	8.72E+05	7.84E+05	8.36E+05	7.33E+05	8.55E+05	2.03E-01	9.80E-01	-2.87E-02	insig
Bartsioside	C15H22O8	Others	Others	2.62E+05	3.98E+05	3.67E+05	7.44E+05	6.62E+05	6.83E+05	1.13E+00	2.04E+00	1.03E+00	up
Pyridoxine-5'-O-glucoside	C14H21NO8	Others	Vitamin	1.09E+06	8.39E+05	1.04E+06	1.91E+05	2.46E+05	1.70E+05	1.19E+00	2.05E-01	-2.29E+00	down
1-(sn-Glycero-3-phospho)-1D-myo-inositol	C9H19O11P	Others	Saccharides and Alcohols	7.18E+05	8.64E+05	6.57E+05	7.75E+05	8.86E+05	6.29E+05	9.34E-02	1.02E+00	3.25E-02	insig
Aquilarone F	C17H18O7	Others	Others	1.78E+04	1.41E+04	1.97E+04	1.80E+04	2.28E+04	1.26E+04	4.20E-02	1.04E+00	5.23E-02	insig
D-Fructose-1,6-biphosphate*	C6H14O12P2	Others	Saccharides and Alcohols	1.48E+04	1.00E+04	6.59E+03	1.81E+04	1.15E+04	2.34E+04	7.87E-01	1.69E+00	7.53E-01	insig
D-Glucose 1,6-bisphosphate*	C6H14O12P2	Others	Saccharides and Alcohols	9.81E+03	7.22E+03	7.07E+03	8.52E+03	9.88E+03	1.00E+04	7.10E-01	1.18E+00	2.39E-01	insig
Cichoriin	C15H16O9	Others	Others	3.33E+06	3.81E+06	2.72E+06	1.53E+07	1.42E+07	1.84E+07	1.19E+00	4.86E+00	2.28E+00	up
D-Maltose	C12H22O11	Others	Saccharides and Alcohols	2.72E+06	7.61E+06	4.96E+06	1.65E+06	1.10E+06	3.17E+06	8.91E-01	3.88E-01	-1.37E+00	insig
D-Trehalose	C12H22O11	Others	Saccharides and Alcohols	2.06E+06	4.86E+06	2.64E+06	8.83E+05	8.44E+05	2.09E+06	9.24E-01	3.99E-01	-1.33E+00	insig
Galactinol	C12H22O11	Others	Saccharides and Alcohols	6.84E+06	1.48E+07	1.35E+07	3.15E+06	2.70E+06	6.26E+06	1.00E+00	3.45E-01	-1.54E+00	down
D-Sucrose	C12H22O11	Others	Saccharides and Alcohols	4.32E+06	8.61E+06	7.25E+06	2.37E+06	2.62E+06	3.73E+06	1.03E+00	4.32E-01	-1.21E+00	down
Lactobiose	C12H22O11	Others	Saccharides and Alcohols	5.08E+06	9.60E+06	9.03E+06	3.05E+06	1.30E+06	4.65E+06	9.30E-01	3.80E-01	-1.40E+00	insig
Melibiose	C12H22O11	Others	Saccharides and Alcohols	1.37E+06	2.81E+06	2.63E+06	9.81E+05	5.98E+05	1.29E+06	9.62E-01	4.21E-01	-1.25E+00	insig
Isomaltulose	C12H22O11	Others	Saccharides and Alcohols	3.69E+06	7.75E+06	7.50E+06	2.19E+06	1.55E+06	2.75E+06	1.04E+00	3.43E-01	-1.54E+00	down
4-Pyridoxic acid-O-glucoside	C14H19NO9	Others	Vitamin	6.51E+04	7.35E+04	5.80E+04	5.06E+04	7.01E+04	4.85E+04	6.15E-01	8.61E-01	-2.17E-01	insig
Turanose	C12H21O11Na	Others	Saccharides and Alcohols	1.62E+05	3.50E+05	2.52E+05	1.73E+05	2.29E+05	1.84E+05	5.12E-01	7.68E-01	-3.81E-01	insig
Byzantionoside A	C19H30O7	Others	Others	5.52E+04	4.85E+04	4.00E+04	2.02E+05	1.79E+05	1.64E+05	1.19E+00	3.79E+00	1.92E+00	up
Riboflavin (Vitamin B2)	C17H20N4O6	Others	Vitamin	7.02E+05	5.72E+05	5.81E+05	1.48E+06	1.48E+06	1.40E+06	1.19E+00	2.35E+00	1.23E+00	up
Trehalose 6-phosphate	C12H23O14P	Others	Saccharides and Alcohols	2.43E+04	3.14E+04	3.41E+04	1.87E+04	1.85E+04	2.33E+04	1.01E+00	6.73E-01	-5.71E-01	insig
Menatetrenone (Vitamin K2)	C31H40O2	Others	Vitamin	1.57E+06	1.41E+06	1.53E+06	1.51E+06	1.57E+06	1.51E+06	2.82E-01	1.02E+00	2.34E-02	insig
Ganoderiol F	C30H46O3	Others	Others	1.21E+05	2.12E+05	1.27E+05	1.53E+04	1.32E+04	1.24E+04	1.18E+00	8.89E-02	-3.49E+00	down
Solatriose	C18H32O15	Others	Saccharides and Alcohols	2.51E+04	4.58E+04	5.02E+04	3.49E+04	2.04E+04	4.48E+04	3.52E-01	8.27E-01	-2.74E-01	insig
D-Panose	C18H32O16	Others	Saccharides and Alcohols	2.35E+04	6.12E+04	4.87E+04	9.13E+03	6.02E+03	7.41E+03	1.13E+00	1.69E-01	-2.56E+00	down
Raffinose	C18H32O16	Others	Saccharides and Alcohols	7.57E+04	1.67E+05	1.42E+05	3.12E+04	1.43E+04	3.33E+04	1.09E+00	2.05E-01	-2.29E+00	down
D-Melezitose	C18H32O16	Others	Saccharides and Alcohols	1.45E+04	5.39E+04	5.70E+04	6.97E+03	4.79E+03	1.33E+04	9.83E-01	2.00E-01	-2.32E+00	insig
Dehydrodiconiferyl alcohol-4-O-glucoside	C26H32O11	Others	Others	1.07E+06	1.09E+06	1.02E+06	1.38E+05	1.54E+05	2.22E+05	1.19E+00	1.61E-01	-2.63E+00	down
Propyl 2-(trimethylammonio)ethyl phosphate	C28H50NO7P	Others	Others	1.06E+05	2.22E+05	2.78E+05	2.20E+04	3.35E+04	3.97E+04	1.13E+00	1.57E-01	-2.67E+00	down
Nystose	C24H42O21	Others	Saccharides and Alcohols	8.81E+03	4.92E+04	3.61E+04	4.84E+02	2.81E+03	3.21E+03	1.04E+00	6.92E-02	-3.85E+00	down
Salicylaldehyde	C7H6O2	Phenolic acids	Phenolic acids	1.18E+04	1.18E+04	1.69E+04	1.42E+04	1.62E+04	1.26E+04	2.63E-01	1.06E+00	8.21E-02	insig
4-Hydroxybenzaldehyde	C7H6O2	Phenolic acids	Phenolic acids	3.31E+05	2.91E+05	2.89E+05	3.10E+05	3.43E+05	3.38E+05	7.31E-01	1.09E+00	1.20E-01	insig
Benzoic acid	C7H6O2	Phenolic acids	Phenolic acids	6.88E+04	3.18E+05	3.74E+05	6.02E+04	6.42E+04	6.21E+04	8.87E-01	2.45E-01	-2.03E+00	insig
2-Methylbenzoic acid	C8H8O2	Phenolic acids	Phenolic acids	4.88E+03	7.16E+03	6.76E+03	1.16E+04	1.27E+04	1.32E+04	1.13E+00	2.00E+00	9.96E-01	insig
4-Aminobenzoic acid	C7H7NO2	Phenolic acids	Phenolic acids	2.16E+04	4.00E+04	3.57E+04	1.05E+04	1.35E+04	9.92E+03	1.11E+00	3.49E-01	-1.52E+00	down
Anthranilic Acid	C7H7NO2	Phenolic acids	Phenolic acids	3.84E+04	5.20E+04	4.92E+04	3.05E+04	3.95E+04	3.52E+04	9.21E-01	7.54E-01	-4.08E-01	insig
2,5-Dihydroxybenzaldehyde	C7H6O3	Phenolic acids	Phenolic acids	1.55E+06	3.26E+06	3.79E+06	5.70E+05	5.81E+05	5.23E+05	1.13E+00	1.95E-01	-2.36E+00	down
4-Hydroxybenzoic acid	C7H6O3	Phenolic acids	Phenolic acids	1.66E+05	2.79E+05	1.76E+05	9.85E+04	3.04E+04	1.28E+05	8.73E-01	4.13E-01	-1.28E+00	insig
Salicylic acid	C7H6O3	Phenolic acids	Phenolic acids	2.21E+06	1.65E+06	1.70E+06	3.75E+06	3.41E+06	3.40E+06	1.15E+00	1.90E+00	9.26E-01	insig
Tyrosol	C8H10O2	Phenolic acids	Phenolic acids	2.59E+05	1.83E+05	1.96E+05	4.46E+05	3.94E+05	3.44E+05	1.11E+00	1.85E+00	8.91E-01	insig
3-Nitrophenol	C6H5NO3	Phenolic acids	Phenolic acids	2.19E+04	1.16E+04	1.52E+04	3.08E+04	2.80E+04	2.68E+04	1.01E+00	1.76E+00	8.14E-01	insig
Phthalic anhydride	C8H4O3	Phenolic acids	Phenolic acids	1.33E+06	1.33E+06	1.30E+06	1.50E+06	1.41E+06	1.38E+06	1.01E+00	1.08E+00	1.17E-01	insig
p-Coumaraldehyde	C9H8O2	Phenolic acids	Phenolic acids	9.26E+03	9.73E+03	1.07E+04	8.49E+03	4.48E+03	4.43E+03	9.51E-01	5.86E-01	-7.71E-01	insig
Hydrocinnamic acid	C9H10O2	Phenolic acids	Phenolic acids	3.23E+05	5.74E+05	7.61E+05	9.00E+00	9.00E+00	9.00E+00	1.20E+00	1.63E-05	-1.59E+01	down
Mandelic acid	C8H8O3	Phenolic acids	Phenolic acids	2.07E+05	2.78E+05	2.02E+05	2.24E+05	1.59E+05	2.26E+05	4.27E-01	8.87E-01	-1.74E-01	insig
4-Aminosalicylic acid	C7H7NO3	Phenolic acids	Phenolic acids	3.33E+04	2.82E+04	2.99E+04	4.39E+03	8.02E+03	5.66E+03	1.18E+00	1.98E-01	-2.34E+00	down
Gentisic Acid	C7H6O4	Phenolic acids	Phenolic acids	5.76E+05	7.65E+05	7.34E+05	6.98E+05	5.23E+05	7.76E+05	1.71E-01	9.62E-01	-5.59E-02	insig
2,4-Dihydroxybenzoic acid	C7H6O4	Phenolic acids	Phenolic acids	1.44E+06	1.86E+06	1.69E+06	1.58E+06	1.14E+06	1.74E+06	4.39E-01	8.95E-01	-1.59E-01	insig
2,6-Dihydroxybenzoic acid	C7H6O4	Phenolic acids	Phenolic acids	7.68E+05	2.39E+06	2.14E+06	1.24E+05	1.20E+05	1.39E+05	1.15E+00	7.23E-02	-3.79E+00	down
3,4-Dihydroxybenzoic acid	C7H6O4	Phenolic acids	Phenolic acids	9.00E+00	9.00E+00	9.00E+00	7.49E+04	7.56E+04	1.01E+05	1.20E+00	9.32E+03	1.32E+01	up
4-MethoxycinnaMaldehyde	C10H10O2	Phenolic acids	Phenolic acids	1.77E+05	1.76E+05	1.80E+05	1.94E+05	2.39E+05	2.15E+05	1.01E+00	1.22E+00	2.83E-01	insig
Caffeic aldehyde	C9H8O3	Phenolic acids	Phenolic acids	1.59E+05	1.57E+05	1.68E+05	1.26E+05	1.53E+05	9.91E+04	8.69E-01	7.81E-01	-3.56E-01	insig
α-Hydroxycinnamic Acid	C9H8O3	Phenolic acids	Phenolic acids	1.07E+05	1.18E+05	1.02E+05	2.03E+05	1.73E+05	2.07E+05	1.17E+00	1.79E+00	8.37E-01	insig
p-Coumaric acid	C9H8O3	Phenolic acids	Phenolic acids	5.90E+04	6.23E+04	4.69E+04	1.29E+04	2.46E+04	3.53E+04	1.00E+00	4.33E-01	-1.21E+00	down
Eugenol	C10H12O2	Phenolic acids	Phenolic acids	7.83E+04	1.30E+05	2.10E+05	6.50E+04	6.36E+04	5.19E+04	9.58E-01	4.31E-01	-1.21E+00	insig
2-(Formylamino)benzoic acid	C8H7NO3	Phenolic acids	Phenolic acids	4.24E+04	2.51E+04	2.68E+04	5.89E+04	5.69E+04	4.96E+04	1.04E+00	1.75E+00	8.10E-01	insig
Terephthalic acid*	C8H6O4	Phenolic acids	Phenolic acids	1.61E+05	1.07E+05	1.32E+05	1.79E+05	1.65E+05	1.39E+05	6.88E-01	1.21E+00	2.72E-01	insig
3-(4-Hydroxyphenyl)-propionic acid	C9H10O3	Phenolic acids	Phenolic acids	9.07E+03	6.21E+03	6.10E+03	1.16E+04	1.04E+04	1.02E+04	1.02E+00	1.51E+00	5.93E-01	insig
2-Amino-3-methoxybenzoic acid	C8H9NO3	Phenolic acids	Phenolic acids	4.82E+05	5.05E+05	5.00E+05	3.35E+05	2.69E+05	3.57E+05	1.11E+00	6.47E-01	-6.29E-01	insig
Homogentisic acid	C8H8O4	Phenolic acids	Phenolic acids	2.06E+05	1.99E+05	1.82E+05	3.15E+04	1.71E+04	2.55E+04	1.18E+00	1.26E-01	-2.99E+00	down
2',4',6'-Trihydroxyacetophenone	C8H8O4	Phenolic acids	Phenolic acids	1.70E+04	6.05E+04	7.12E+04	9.00E+00	5.64E+03	8.17E+03	8.11E-01	9.29E-02	-3.43E+00	insig

Vanillic acid	C8H8O4	Phenolic acids	Phenolic acids	6.54E+04	1.04E+05	1.06E+05	5.44E+05	4.70E+05	4.84E+05	1.18E+00	5.43E+00	2.44E+00	up
3,4-Dihydroxybenzeneacetic acid	C8H8O4	Phenolic acids	Phenolic acids	2.06E+05	2.13E+05	1.11E+05	9.00E+00	9.00E+00	9.00E+00	1.20E+00	5.09E-05	-1.43E+01	down
Coniferaldehyde	C10H10O3	Phenolic acids	Phenolic acids	5.74E+04	9.41E+04	1.09E+05	4.29E+04	2.32E+04	5.32E+04	9.41E-01	4.58E-01	-1.13E+00	insig
4-Methoxycinnamic acid	C10H10O3	Phenolic acids	Phenolic acids	4.25E+05	3.87E+05	2.21E+05	8.48E+03	6.00E+03	1.28E+04	1.18E+00	2.64E-02	-5.24E+00	down
p-Coumaric acid methyl ester	C10H10O3	Phenolic acids	Phenolic acids	4.09E+05	3.55E+05	2.00E+05	9.00E+00	9.00E+00	9.00E+00	1.20E+00	2.80E-05	-1.51E+01	down
Caffeic acid	C9H8O4	Phenolic acids	Phenolic acids	1.38E+06	3.13E+06	2.13E+06	1.48E+06	1.54E+06	1.75E+06	5.97E-01	7.18E-01	-4.78E-01	insig
Coniferyl alcohol	C10H12O3	Phenolic acids	Phenolic acids	1.38E+04	2.16E+04	2.19E+04	9.00E+00	9.00E+00	9.00E+00	1.20E+00	4.72E-04	-1.10E+01	down
Dihydrocaffeic acid	C9H10O4	Phenolic acids	Phenolic acids	3.17E+04	3.78E+04	2.92E+04	1.76E+04	1.55E+04	1.53E+04	1.16E+00	4.90E-01	-1.03E+00	down
Syringaldehyde; 4-Hydroxy-3,5-Dimethoxybenzaldehyde	C9H10O4	Phenolic acids	Phenolic acids	1.06E+04	4.86E+03	5.23E+03	4.18E+04	3.79E+04	3.45E+04	1.15E+00	5.51E+00	2.46E+00	up
2,4-Dinitrophenol	C6H4N2O5	Phenolic acids	Phenolic acids	1.48E+04	2.29E+04	1.88E+04	1.91E+04	2.50E+04	2.38E+04	6.42E-01	1.20E+00	2.67E-01	insig
Isoferulic Acid	C10H10O4	Phenolic acids	Phenolic acids	7.42E+03	1.10E+04	8.19E+03	1.10E+04	7.02E+03	8.67E+03	1.71E-02	1.00E+00	3.34E-03	insig
Ferulic acid	C10H10O4	Phenolic acids	Phenolic acids	7.87E+03	5.86E+03	5.84E+03	1.39E+04	1.07E+04	1.02E+04	1.09E+00	1.78E+00	8.30E-01	insig
Methyl caffeate	C10H10O4	Phenolic acids	Phenolic acids	5.39E+04	3.27E+05	3.23E+05	6.97E+05	6.14E+05	6.77E+05	8.85E-01	2.83E+00	1.50E+00	insig
4-Hydroxy-3-methoxymandelate	C9H10O5	Phenolic acids	Phenolic acids	5.31E+04	5.09E+04	5.17E+04	3.09E+04	5.49E+04	3.87E+04	7.34E-01	8.00E-01	-3.23E-01	insig
Syringic acid	C9H10O5	Phenolic acids	Phenolic acids	9.00E+00	9.00E+00	9.00E+00	1.32E+04	2.29E+04	1.73E+04	1.20E+00	1.98E+03	1.09E+01	up
4-Hydroxy-3,5-diisopropylbenzaldehyde	C13H18O2	Phenolic acids	Phenolic acids	1.16E+04	5.20E+03	8.76E+03	8.13E+03	1.34E+04	7.64E+03	2.87E-01	1.14E+00	1.90E-01	insig
2,4-Di-Tert-Butylphenol	C14H22O	Phenolic acids	Phenolic acids	1.44E+06	1.03E+06	1.32E+06	1.47E+06	1.61E+06	1.75E+06	8.82E-01	1.27E+00	3.50E-01	insig
2,6-Di-tert-butylphenol	C14H22O	Phenolic acids	Phenolic acids	1.29E+06	1.10E+06	1.26E+06	1.43E+06	1.65E+06	2.02E+06	9.83E-01	1.39E+00	4.79E-01	insig
Sinapinaldehyde	C11H12O4	Phenolic acids	Phenolic acids	5.83E+04	6.79E+04	5.57E+04	6.52E+04	5.08E+04	6.48E+04	3.21E-02	9.95E-01	-7.96E-03	insig
3,4-Dimethoxycinnamic acid	C11H12O4	Phenolic acids	Phenolic acids	8.58E+03	1.09E+04	1.85E+04	9.00E+00	9.00E+00	9.00E+00	1.20E+00	7.11E-04	-1.05E+01	down
Ethyl caffeate	C11H12O4	Phenolic acids	Phenolic acids	1.16E+04	1.56E+04	2.31E+04	9.00E+00	9.00E+00	9.00E+00	1.20E+00	5.36E-04	-1.09E+01	down
Oresbiusin A	C10H12O5	Phenolic acids	Phenolic acids	2.52E+04	2.01E+04	2.17E+04	1.09E+05	1.00E+05	1.42E+05	1.19E+00	5.24E+00	2.39E+00	up
3,4'-Dihydroxy-3'-methoxybenzenepentanoic acid	C12H16O5	Phenolic acids	Phenolic acids	6.36E+05	9.31E+05	8.88E+05	1.33E+05	1.47E+05	1.41E+05	1.19E+00	1.71E-01	-2.54E+00	down
p-Hydroxybenzoylmalic acid	C11H10O7	Phenolic acids	Phenolic acids	2.21E+05	1.30E+05	2.07E+05	9.00E+00	9.00E+00	9.00E+00	1.20E+00	4.84E-05	-1.43E+01	down
Benzyl glucoside	C13H18O6	Phenolic acids	Phenolic acids	5.84E+03	2.52E+03	3.44E+03	1.40E+03	9.00E+00	2.02E+03	6.90E-01	2.91E-01	-1.78E+00	insig
Dibutyl phthalate	C16H22O4	Phenolic acids	Phenolic acids	3.81E+07	3.70E+07	3.72E+07	4.17E+07	3.97E+07	3.90E+07	1.02E+00	1.07E+00	1.00E-01	insig
Diisobutyl phthalate	C16H22O4	Phenolic acids	Phenolic acids	3.78E+07	3.63E+07	3.78E+07	4.14E+07	3.93E+07	3.81E+07	8.84E-01	1.06E+00	8.59E-02	insig
Cinnamoyltartaric acid	C13H12O7	Phenolic acids	Phenolic acids	7.81E+04	6.05E+04	6.61E+04	1.95E+03	2.79E+03	3.44E+03	1.19E+00	3.99E-02	-4.65E+00	down
Phenethyl caffeate	C17H16O4	Phenolic acids	Phenolic acids	5.65E+03	9.33E+03	8.55E+03	2.29E+03	2.91E+03	2.97E+03	1.14E+00	3.47E-01	-1.53E+00	down
3-Hydroxy-5-Methylphenol-1-O-Glucoside	C13H18O7	Phenolic acids	Phenolic acids	4.15E+04	2.26E+05	1.18E+05	3.38E+05	3.65E+05	3.87E+05	9.44E-01	2.83E+00	1.50E+00	insig
Brevifolin carboxylic acid	C13H8O8	Phenolic acids	Phenolic acids	2.70E+04	3.41E+04	4.33E+04	3.34E+04	2.24E+04	8.68E+03	6.98E-01	6.17E-01	-6.96E-01	insig
4-O-Glucosyl-4-hydroxybenzoic acid	C13H16O8	Phenolic acids	Phenolic acids	5.28E+05	4.41E+05	5.14E+05	7.69E+04	6.66E+04	8.43E+04	1.20E+00	1.54E-01	-2.70E+00	down
1-O-Salicyl-D-glucose	C13H16O8	Phenolic acids	Phenolic acids	1.52E+07	2.54E+07	2.01E+07	1.38E+07	1.12E+07	1.33E+07	9.67E-01	6.29E-01	-6.68E-01	insig
Salicylic acid-2-O-glucoside	C13H16O8	Phenolic acids	Phenolic acids	5.14E+06	8.02E+06	8.16E+06	8.63E+05	7.43E+05	8.48E+05	1.19E+00	1.15E-01	-3.12E+00	down
4-O-Glucosyl-3,4-dihydroxybenzyl alcohol	C13H18O8	Phenolic acids	Phenolic acids	9.21E+04	9.50E+04	8.15E+04	1.61E+05	1.47E+05	1.60E+05	1.18E+00	1.74E+00	7.98E-01	insig
Feruloylmalic acid	C14H14O8	Phenolic acids	Phenolic acids	2.54E+04	1.97E+04	1.26E+04	5.36E+04	3.14E+04	3.16E+04	9.67E-01	2.02E+00	1.02E+00	insig
Mandelic acid-β-glucoside	C14H18O8	Phenolic acids	Phenolic acids	2.51E+04	3.76E+04	4.65E+04	1.84E+04	2.00E+04	2.12E+04	1.01E+00	5.46E-01	-8.73E-01	insig
1-O-Gentisoyl-D-glucoside	C13H16O9	Phenolic acids	Phenolic acids	2.65E+07	2.27E+07	2.44E+07	1.06E+07	7.22E+06	9.08E+06	1.16E+00	3.66E-01	-1.45E+00	down
Protocatechuic acid-4-O-glucoside	C13H16O9	Phenolic acids	Phenolic acids	2.19E+07	2.44E+07	2.61E+07	4.91E+06	4.26E+06	4.95E+06	1.20E+00	1.95E-01	-2.36E+00	down
Ginkgolic Acid C13:2	C20H28O3	Phenolic acids	Phenolic acids	2.98E+05	2.15E+05	2.03E+05	6.23E+04	7.09E+04	6.55E+04	1.18E+00	2.77E-01	-1.85E+00	down
Trans-5-O-(p-Coumaroyl)shikimate	C16H16O7	Phenolic acids	Phenolic acids	1.13E+05	1.12E+05	1.19E+05	1.65E+04	9.03E+03	1.62E+04	1.18E+00	1.21E-01	-3.04E+00	down
3-O-p-Coumaroylshikimic acid	C16H16O7	Phenolic acids	Phenolic acids	2.72E+04	3.09E+04	2.62E+04	4.69E+03	4.70E+03	9.98E+03	1.14E+00	2.30E-01	-2.12E+00	down
1-O-[(E)-p-Cumaroyl]-D-glucose	C15H18O8	Phenolic acids	Phenolic acids	7.22E+05	5.91E+05	4.77E+05	1.79E+05	1.91E+05	1.95E+05	1.17E+00	3.15E-01	-1.67E+00	down
p-Coumaric acid-4-O-glucoside	C15H18O8	Phenolic acids	Phenolic acids	3.11E+05	3.41E+05	3.06E+05	4.26E+05	3.72E+05	4.57E+05	1.08E+00	1.31E+00	3.91E-01	insig
5-Glucosyloxy-2-Hydroxybenzoic acid methyl ester	C14H18O9	Phenolic acids	Phenolic acids	6.83E+04	3.77E+04	2.81E+04	1.51E+04	1.71E+04	2.99E+04	8.93E-01	4.62E-01	-1.11E+00	insig
Vanillic acid-4-O-glucoside	C14H18O9	Phenolic acids	Phenolic acids	4.35E+03	1.61E+04	1.15E+04	3.16E+04	1.44E+04	2.44E+04	8.40E-01	2.21E+00	1.14E+00	insig
3,4,5-Trimethoxyphenyl-1-O-Glucoside	C15H22O8	Phenolic acids	Phenolic acids	2.43E+06	3.67E+06	3.56E+06	9.43E+06	7.93E+06	8.71E+06	1.16E+00	2.70E+00	1.43E+00	up
Koaburaside	C14H20O9	Phenolic acids	Phenolic acids	1.01E+05	1.05E+05	9.85E+04	2.41E+04	1.87E+04	2.31E+04	1.19E+00	2.17E-01	-2.21E+00	down
p-Dimeric galloyl methyl ester	C15H12O9	Phenolic acids	Phenolic acids	5.59E+03	9.00E+00	3.91E+03	2.95E+04	2.94E+04	3.12E+04	8.18E-01	9.46E+00	3.24E+00	insig
5-O-Caffeoylshikimic acid	C16H16O8	Phenolic acids	Phenolic acids	3.24E+05	2.30E+05	4.30E+05	9.00E+00	9.00E+00	9.00E+00	1.20E+00	2.74E-05	-1.52E+01	down
1-O-p-Coumaroylquinic acid	C16H18O8	Phenolic acids	Phenolic acids	7.37E+03	2.06E+04	2.21E+04	1.16E+05	1.13E+05	1.57E+05	1.13E+00	7.72E+00	2.95E+00	up
3-O-p-Coumaroylquinic acid	C16H18O8	Phenolic acids	Phenolic acids	7.38E+05	2.92E+06	2.09E+06	7.91E+05	1.06E+06	1.40E+06	5.39E-01	5.67E-01	-8.19E-01	insig
4-O-p-Coumaroylquinic acid	C16H18O8	Phenolic acids	Phenolic acids	2.39E+05	7.22E+05	6.02E+05	2.52E+05	2.51E+05	4.09E+05	6.29E-01	5.83E-01	-7.78E-01	insig
5-O-p-Coumaroylquinic acid	C16H18O8	Phenolic acids	Phenolic acids	6.77E+05	2.08E+06	2.17E+06	8.80E+05	8.35E+05	1.28E+06	5.27E-01	6.08E-01	-7.18E-01	insig
Sinapoyl malate	C15H16O9	Phenolic acids	Phenolic acids	2.91E+04	1.88E+04	2.46E+04	4.30E+03	4.07E+03	3.42E+03	1.19E+00	1.63E-01	-2.62E+00	down
1-O-[(E)-Caffeoyl]-D-glucose	C15H18O9	Phenolic acids	Phenolic acids	1.05E+06	3.12E+06	2.13E+06	2.84E+05	4.51E+05	2.53E+05	1.11E+00	1.57E-01	-2.67E+00	down
6-O-Caffeoyl-D-glucose	C15H18O9	Phenolic acids	Phenolic acids	1.73E+06	5.52E+06	3.72E+06	6.83E+05	8.97E+05	5.65E+05	1.09E+00	1.96E-01	-2.35E+00	down
Coniferin	C16H22O8	Phenolic acids	Phenolic acids	2.15E+06	2.08E+06	2.71E+06	8.46E+05	1.00E+06	1.11E+06	1.16E+00	4.25E-01	-1.23E+00	down
Dihydrocaffeoylglucose	C15H20O9	Phenolic acids	Phenolic acids	4.19E+05	6.40E+05	5.74E+05	1.49E+05	1.30E+05	1.18E+05	1.18E+00	2.43E-01	-2.04E+00	down
1-Caffeoylquinic acid	C16H18O9	Phenolic acids	Phenolic acids	2.37E+05	7.73E+05	4.38E+05	5.41E+05	5.24E+05	1.57E+06	6.01E-01	1.82E+00	8.66E-01	insig
Chlorogenic acid (3-O-Caffeoylquinic acid)	C16H18O9	Phenolic acids	Phenolic acids	2.52E+05	3.54E+05	2.96E+05	3.04E+04	3.32E+04	3.36E+04	1.20E+00	1.08E-01	-3.21E+00	down
Cryptochlorogenic acid (4-O-Caffeoylquinic acid)	C16H18O9	Phenolic acids	Phenolic acids	2.65E+06	2.85E+06	2.54E+06	2.31E+06	2.80E+06	2.60E+06	3.91E-01	9.59E-01	-6.09E-02	insig
Neochlorogenic acid (5-O-Caffeoylquinic acid)	C16H18O9	Phenolic acids	Phenolic acids	7.72E+04	1.13E+05	8.55E+04	4.15E+05	3.64E+05	4.57E+05	1.19E+00	4.49E+00	2.17E+00	up
Sinapoyltartaric acid	C15H16O10	Phenolic acids	Phenolic acids	2.23E+03	3.82E+03	3.05E+03	4.74E+03	4.61E+03	5.56E+03	1.01E+00	1.64E+00	7.14E-01	insig
1-O-Feruloyl-D-Glucose*	C16H20O9	Phenolic acids	Phenolic acids	2.07E+07	2.30E+07	2.39E+07	1.94E+07	1.93E+07	2.05E+07	9.84E-01	8.76E-01	-1.90E-01	insig
6-O-Feruloyl-D-glucose*	C16H20O9	Phenolic acids	Phenolic acids	9.52E+05	1.21E+06	9.95E+05	7.54E+05	5.59E+05	8.05E+05	9.94E-01	6.71E-01	-5.75E-01	insig
5-O-Galloyl-methyl quinine ester	C15H18O10	Phenolic acids	Phenolic acids	1.67E+04	7.65E+03	1.22E+04	4.74E+03	8.67E+03	2.13E+03	8.67E-01	4.26E-01	-1.23E+00	insig
Rosmarinic acid	C18H16O8	Phenolic acids	Phenolic acids	1.78E+05	1.12E+05	1.29E+05	1.74E+05	1.61E+05	1.55E+05	6.37E-01	1.17E+00	2.27E-01	insig
Glucosyringic Acid	C15H20O10	Phenolic acids	Phenolic acids	3.95E+05	4.24E+05	3.66E+05	2.57E+05	2.42E+05	2.13E+05	1.16E+00	6.01E-01	-7.34E-01	insig
5-O-Feruloylquinic acid	C17H20O9	Phenolic acids	Phenolic acids	1.78E+06	2.73E+06	1.75E+06	2.15E+06	1.53E+06	2.72E+06	6.08E-02	1.02E+00	3.26E-02	insig
1-O-Feruloylquinic acid	C17H20O9	Phenolic acids	Phenolic acids	1.69E+06	2.37E+06	2.18E+06	2.01E+06	1.60E+06	2.17E+06	3.03E-01	9.27E-01	-1.09E-01	insig
Chlorogenic acid methyl ester	C17H20O9	Phenolic acids	Phenolic acids	5.60E+05	6.67E+05	6.13E+05	6.43E+05	4.84E+05	6.76E+05	1.34E-01	9.80E-01	-2.96E-02	insig
3-O-Feruloylquinic acid	C17H20O9	Phenolic acids	Phenolic acids	1.06E+05	8.28E+04	5.16E+04	8.99E+04	6.06E+04	8.63E+04	4.52E-02	9.85E-01	-2.23E-02	insig
Sinapaldehyde-4-O-Glucoside	C17H22O9	Phenolic acids	Phenolic acids	7.08E+04	5.33E+04	5.07E+04	6.30E+05	6.61E+05	6.40E+05	1.20E+00	1.11E+01	3.47E+00	up
Trihydroxycinnamoylquinic acid	C16H20O10	Phenolic acids	Phenolic acids	2.86E+04	5.98E+04	3.02E+04	6.85E+03	4.35E+03	5.14E+03	1.15E+00	1.38E-01	-2.86E+00	down
Syringin	C17H24O9	Phenolic acids	Phenolic acids	2.17E+06	3.32E+06	5.55E+06	4.40E+05	4.35E+05	5.59E+05	1.16E+00	1.30E-01	-2.94E+00	down
Feruloyl syringic acid	C19H18O8	Phenolic acids	Phenolic acids	9.00E+00	9.00E+00	9.00E+00	1.22E+04	9.44E+03	1.14E+04	1.20E+00	1.22E+03	1.03E+01	up
1-O-Glucosyl sinapate	C17H22O10	Phenolic acids	Phenolic acids	5.60E+03	1.73E+04	3.11E+04	9.80E+04	8.91E+04	7.78E+04	1.04E+00	4.90E+00	2.29E+00	up
4-O-Glucosyl-sinapate	C17H22O10	Phenolic acids	Phenolic acids	9.14E+03	9.00E+00	9.00E+00	3.34E+06	3.21E+06	2.92E+06	1.10E+00	1.03E+03	1.00E+01	up
1-O-Sinapoyl-D-glucose	C17H22O10	Phenolic acids	Phenolic acids	1.28E+04	6.28E+03	8.96E+03	4.72E+05	4.82E+05	6.42E+05	1.19E+00	5.68E+01	5.83E+00	up
5'-Glucosyloxyjasmanic acid	C18H28O9	Phenolic acids	Phenolic acids	1.50E+07	1.96E+07	1.77E+07	4.28E+07	4.54E+07	4.25E+07	1.18E+00	2.50E+00	1.32E+00	up
Bis(2-ethylhexyl)phthalate	C24H38O4	Phenolic acids	Phenolic acids	3.65E+07	4.00E+07	4.64E+07	3.85E+07	4.23E+07	4.12E+07	5.15E-02	9.91E-01	-1.27E-02	insig
Diisooctyl Phthalate	C24H38O4	Phenolic acids	Phenolic acids	3.55E+07	4.00E+07	4.33E+07	3.54E+07	4.00E+07	3.86E+07	3.47E-01	9.60E-01	-5.95E-02	insig
Sinapoylglucuronic acid	C17H20O11	Phenolic acids	Phenolic acids	9.00E+00	9.00E+00	9.00E+00	1.23E+05	6.99E+04	1.09E+05	1.20E+00	1.12E+04	1.34E+01	up
Benzyl-(2"-O-xylosyl)glucoside	C18H26O10	Phenolic acids	Phenolic acids	9.71E+05	6.06E+05	6.97E+05	6.13E+05	6.84E+05	6.20E+05	5.65E-01	8.43E-01	-2.47E-01	insig
Caffeoylnicotinoyltartaric acid	C19H13NO10	Phenolic acids	Phenolic acids	5.33E+05	5.39E+05	7.28E+05	1.78E+05	1.73E+05	1.21E+05	1.17E+00	2.63E-01	-1.93E+00	down
Caffeoylbenzoyltartaric acid	C20H16O10	Phenolic acids	Phenolic acids	5.34E+04	5.62E+04	4.06E+04	1.89E+04	1.38E+04	9.42E+03	1.13E+00	2.80E-01	-1.84E+00	down
Cimicifugic acid C	C20H18O10	Phenolic acids	Phenolic acids	9.00E+00	9.00E+00	9.00E+00	5.22E+06	4.08E+06	4.91E+06	1.20E+00	5.26E+05	1.90E+01	up
Grandidentatin	C21H28O9	Phenolic acids	Phenolic acids	8.52E+03	2.67E+04	1.91E+04	5.32E+03	2.92E+03	1.19E+04	8.42E-01	3.71E-01	-1.43E+00	insig
Cimicifugic acid E	C21H20O10	Phenolic acids	Phenolic acids	6.71E+04	7.04E+04	7.46E+04	9.00E+00	9.00E+00	9.00E+00	1.20E+00	1.27E-04	-1.29E+01	down
Bis(p-Coumaroyl)tartaric acid	C22H18O10	Phenolic acids	Phenolic acids	1.08E+04	1.68E+04	2.58E+04	8.81E+03	1.21E+04	1.25E+04	7.38E-01	6.26E-01	-6.76E-01	insig
6-O-Glucosyl-caffeoylbenzoic acid	C22H22O10	Phenolic acids	Phenolic acids	1.03E+04	1.31E+04	1.02E+04	9.00E+00	6.64E+03	9.00E+00	9.04E-01	1.98E-01	-2.33E+00	insig
p-Coumaroylcaffeoyltartaric acid	C22H18O11	Phenolic acids	Phenolic acids	1.10E+04	2.06E+04	1.56E+04	9.00E+00	9.00E+00	9.00E+00	1.20E+00	5.72E-04	-1.08E+01	down
Caffeoyl-p-coumaroyltartaric acid	C22H18O11	Phenolic acids	Phenolic acids	1.59E+06	2.16E+06	1.77E+06	6.50E+06	5.84E+06	6.25E+06	1.19E+00	3.37E+00	1.75E+00	up
Anthranilate-1-O-Sophoroside	C19H27NO12	Phenolic acids	Phenolic acids	5.97E+05	1.11E+06	1.14E+06	6.72E+05	3.04E+05	8.51E+05	6.48E-01	6.43E-01	-6.38E-01	insig
Vnilloylcaffeoyltartaric acid	C21H18O12	Phenolic acids	Phenolic acids	8.13E+03	2.19E+04	1.30E+04	9.00E+00	9.00E+00	9.00E+00	1.20E+00	6.28E-04	-1.06E+01	down
4-O-(6'-O-Glucosylcaffeoyl)-4-hydroxybenzoic acid	C22H22O11	Phenolic acids	Phenolic acids	2.08E+06	2.19E+06	2.82E+06	6.84E+05	6.34E+05	6.77E+05	1.19E+00	2.81E-01	-1.83E+00	down
Sibiricose A3	C19H26O13	Phenolic acids	Phenolic acids	1.57E+04	1.08E+04	1.86E+04	7.60E+03	3.39E+03	1.12E+04	8.58E-01	4.93E-01	-1.02E+00	insig
4-p-Cumaroyl-rhamnosyl-(1→6)-D-glucose	C21H28O12	Phenolic acids	Phenolic acids	1.03E+06	8.26E+05	1.15E+06	6.31E+05	5.55E+05	6.70E+05	1.09E+00	6.18E-01	-6.95E-01	insig
4-O-(6'-O-Glucosylcaffeoyl)-3,4-dihydroxybenzoic acid	C22H22O12	Phenolic acids	Phenolic acids	1.03E+06	1.07E+06	8.70E+05	5.44E+05	4.01E+05	4.83E+05	1.15E+00	4.81E-01	-1.06E+00	down
3-O-p-Coumaroylshikimic acid-O-glucoside	C22H26O12	Phenolic acids	Phenolic acids	1.09E+04	7.38E+03	9.63E+03	2.64E+04	1.36E+04	2.52E+04	1.04E+00	2.33E+00	1.22E+00	up
Juglanoside J	C25H26O10	Phenolic acids	Phenolic acids	5.97E+04	2.34E+05	2.14E+05	3.88E+05	3.97E+05	2.86E+05	8.43E-01	2.11E+00	1.08E+00	insig
Dicaffeoylshikimic acid	C25H22O11	Phenolic acids	Phenolic acids	7.63E+05	8.66E+05	8.61E+05	1.27E+06	1.26E+06	1.38E+06	1.17E+00	1.57E+00	6.49E-01	insig
3-O-p-Coumaroylquinic acid-O-glucoside	C22H28O13	Phenolic acids	Phenolic acids	5.10E+03	1.16E+04	7.21E+03	5.55E+03	7.38E+03	1.06E+04	1.10E-02	9.84E-01	-2.29E-02	insig
Sinapoyl-p-coumaroyltartaric acid	C24H22O12	Phenolic acids	Phenolic acids	1.25E+05	3.45E+05	2.99E+05	1.40E+05	1.23E+05	2.16E+05	6.02E-01	6.21E-01	-6.86E-01	insig
3,6-Di-O-caffeoyl glucose	C24H24O12	Phenolic acids	Phenolic acids	1.16E+05	4.75E+05	6.83E+05	2.97E+05	2.60E+05	2.93E+05	1.86E-01	6.68E-01	-5.82E-01	insig
((2R,3R,5R,6R)-6-(((E)-3-(3,4-dihydroxyphenyl)acryloyl)oxy)-3,4,5-trihydroxytetrahydro-2H-pyran-2-yl)methyl (Z)-3-(3,4-dihydroxyphenyl)acrylate	C24H24O12	Phenolic acids	Phenolic acids	8.20E+04	2.13E+05	1.58E+05	1.44E+05	2.37E+05	2.05E+05	5.21E-01	1.29E+00	3.73E-01	insig
Isochlorogenic acid B	C25H24O12	Phenolic acids	Phenolic acids	1.65E+07	2.03E+07	1.84E+07	1.69E+07	1.52E+07	1.79E+07	6.33E-01	9.06E-01	-1.42E-01	insig
Isochlorogenic acid A	C25H24O12	Phenolic acids	Phenolic acids	3.24E+05	1.10E+06	5.68E+05	7.71E+05	9.12E+05	1.03E+06	6.06E-01	1.36E+00	4.41E-01	insig
Isochlorogenic acid C	C25H24O12	Phenolic acids	Phenolic acids	1.47E+07	1.37E+07	1.31E+07	1.41E+07	1.29E+07	1.51E+07	1.47E-01	1.01E+00	2.03E-02	insig
Cynarin	C25H24O12	Phenolic acids	Phenolic acids	1.66E+06	3.74E+06	2.62E+06	2.86E+07	2.82E+07	3.19E+07	1.18E+00	1.11E+01	3.47E+00	up
3,5-Dicaffeoylquinic acid	C25H24O12	Phenolic acids	Phenolic acids	1.49E+06	3.19E+06	2.61E+06	2.30E+07	2.43E+07	2.61E+07	1.18E+00	1.01E+01	3.33E+00	up
Di-O-Glucosylquinic acid	C19H32O16	Phenolic acids	Phenolic acids	1.96E+05	9.27E+05	5.21E+05	1.68E+05	8.77E+04	2.78E+05	8.14E-01	3.24E-01	-1.63E+00	insig
3,5-O-Dicaffeoylquinic Acid Methyl Ester	C26H26O12	Phenolic acids	Phenolic acids	1.62E+06	2.44E+06	1.90E+06	1.86E+06	2.11E+06	2.19E+06	2.12E-01	1.03E+00	4.94E-02	insig
3,4-O-Dicaffeoylquinic Acid Methyl Ester	C26H26O12	Phenolic acids	Phenolic acids	1.58E+05	6.35E+05	3.20E+05	1.83E+06	7.25E+05	2.19E+06	9.89E-01	4.26E+00	2.09E+00	insig
Feruloylsinapoyltartaric acid	C25H24O13	Phenolic acids	Phenolic acids	1.55E+04	3.98E+04	2.71E+04	4.06E+04	2.12E+04	3.65E+04	3.68E-01	1.19E+00	2.56E-01	insig
Sinapoylsinapoyltartaric acid	C26H26O14	Phenolic acids	Phenolic acids	1.62E+04	1.66E+04	2.54E+04	8.98E+03	7.72E+03	1.32E+04	1.01E+00	5.13E-01	-9.62E-01	insig
Disinapoyl glucoside	C28H32O14	Phenolic acids	Phenolic acids	1.34E+05	4.77E+04	4.44E+04	8.00E+04	5.38E+04	6.13E+04	3.04E-02	8.64E-01	-2.11E-01	insig
4-O-(6'-O-Glucosylcaffeoylglucosyl)-4-hydroxybenzyl alcohol	C28H34O15	Phenolic acids	Phenolic acids	1.98E+04	5.53E+03	6.70E+03	9.35E+04	1.05E+05	7.21E+04	1.13E+00	8.45E+00	3.08E+00	up
3,4,5-Tri-O-Galloylshikimic acid	C28H22O17	Phenolic acids	Phenolic acids	1.22E+04	1.10E+04	6.12E+03	4.82E+03	8.39E+03	7.41E+03	6.31E-01	7.04E-01	-5.07E-01	insig
3,4,5-Tricaffeoylquinic acid	C34H30O15	Phenolic acids	Phenolic acids	5.67E+05	1.23E+06	1.12E+06	3.11E+06	1.88E+06	3.32E+06	1.05E+00	2.84E+00	1.51E+00	up
Dicaffeoylquinic acid-O-glucoside	C31H34O17	Phenolic acids	Phenolic acids	5.67E+05	3.50E+05	4.00E+05	5.98E+05	6.36E+05	4.58E+05	7.17E-01	1.29E+00	3.62E-01	insig
Syringoylcaffeoylquinic acid-D-glucose	C31H36O18	Phenolic acids	Phenolic acids	1.64E+05	6.19E+05	4.04E+05	2.71E+04	2.46E+04	2.09E+04	1.15E+00	6.12E-02	-4.03E+00	down
3-Methoxy-juglone	C11H8O4	Quinones	Quinones	2.04E+06	3.54E+06	3.12E+06	4.02E+06	3.85E+06	3.86E+06	8.37E-01	1.35E+00	4.30E-01	insig

HydroxyAloe-emodin-8-O-glucoside	C21H20O11	Quinones	Anthraquinone	5.36E+06	1.94E+06	2.63E+06	8.52E+05	1.04E+06	1.40E+06	1.01E+00	3.33E-01	-1.59E+00	down
Emodin-8-O-(6"-O-acetyl)glucoside	C23H22O11	Quinones	Anthraquinone	8.19E+03	7.84E+03	8.00E+03	9.26E+03	7.55E+03	9.38E+03	6.04E-01	1.09E+00	1.24E-01	insig
1,4,8-Trihydroxynaphthalene-1-O-[6'-O-(3",4",5"-trimethylbenzoyl)]glucoside	C25H24O11	Quinones	Quinones	3.06E+06	8.10E+06	7.96E+06	1.01E+06	1.20E+06	1.78E+06	1.08E+00	2.09E-01	-2.26E+00	down
Tigogenin	C27H44O3	Steroids	Steroidal saponins	1.17E+04	2.59E+04	1.35E+04	1.92E+04	1.60E+04	1.44E+04	7.03E-02	9.70E-01	-4.35E-02	insig
5-O-Galloyl-D-hamamelose*	C13H16O10	Tannins	Tannin	1.39E+05	1.10E+05	1.34E+05	1.46E+05	1.71E+05	1.04E+05	2.71E-01	1.10E+00	1.36E-01	insig
1-O-Galloyl-D-glucose	C13H16O10	Tannins	Tannin	2.19E+04	1.55E+05	5.95E+04	9.00E+00	9.00E+00	9.00E+00	1.19E+00	1.14E-04	-1.31E+01	down
3-O-Galloyl-D-glucose*	C13H16O10	Tannins	Tannin	1.57E+05	1.09E+05	1.06E+05	1.76E+05	1.37E+05	1.40E+05	6.98E-01	1.22E+00	2.85E-01	insig
Di-O-galloyl Methyl gallate	C22H32O12	Tannins	Tannin	1.46E+05	3.72E+04	8.96E+04	3.70E+04	6.09E+04	2.90E+04	7.19E-01	4.66E-01	-1.10E+00	insig
Perillyl alcohol	C10H16O	Terpenoids	Monoterpenoids	1.31E+05	9.76E+04	5.98E+04	1.61E+05	1.57E+05	1.31E+05	8.68E-01	1.56E+00	6.39E-01	insig
Arteannuin A	C13H18O2	Terpenoids	Sesquiterpenoids	2.48E+05	8.12E+05	4.19E+05	1.33E+06	1.19E+06	9.13E+05	9.60E-01	2.33E+00	1.22E+00	insig
Cryptomerione	C15H22O	Terpenoids	Sesquiterpenoids	3.57E+04	5.22E+04	4.16E+04	9.00E+00	9.00E+00	9.00E+00	1.20E+00	2.08E-04	-1.22E+01	down
Nootkatol	C15H24O	Terpenoids	Sesquiterpenoids	1.50E+05	3.58E+05	4.24E+05	9.00E+00	9.00E+00	9.00E+00	1.20E+00	2.90E-05	-1.51E+01	down
Oxyphyllol A	C15H24O	Terpenoids	Sesquiterpenoids	7.15E+05	1.42E+06	1.56E+06	9.00E+00	9.00E+00	9.00E+00	1.20E+00	7.31E-06	-1.71E+01	down
Elemol	C15H26O	Terpenoids	Sesquiterpenoids	1.96E+06	1.69E+06	1.77E+06	2.34E+06	2.15E+06	2.34E+06	1.10E+00	1.26E+00	3.34E-01	insig
Genipin	C11H14O5	Terpenoids	Monoterpenoids	4.06E+06	4.75E+06	4.53E+06	9.81E+06	1.04E+07	1.08E+07	1.19E+00	2.33E+00	1.22E+00	up
Chloranthalactone A	C15H16O2	Terpenoids	Sesquiterpenoids	4.71E+05	6.99E+05	7.33E+05	9.00E+00	9.00E+00	9.00E+00	1.20E+00	1.42E-05	-1.61E+01	down
1-Oxo-9-desoxy cacalol	C15H16O2	Terpenoids	Sesquiterpenoids	8.65E+04	1.88E+05	1.98E+05	9.00E+00	9.00E+00	9.00E+00	1.20E+00	5.72E-05	-1.41E+01	down
Lindenenone	C15H18O2	Terpenoids	Sesquiterpenoids	1.83E+04	1.62E+04	1.23E+04	5.25E+04	5.30E+04	6.88E+04	1.17E+00	3.72E+00	1.90E+00	up
Dehydrocostuslactone	C15H18O2	Terpenoids	Sesquiterpenoids	2.86E+05	2.95E+05	2.12E+05	9.00E+00	9.00E+00	9.00E+00	1.20E+00	3.40E-05	-1.48E+01	down
Nicosesquiterpene A	C15H20O2	Terpenoids	Sesquiterpenoids	4.41E+04	9.35E+04	1.02E+05	3.57E+05	2.81E+05	3.24E+05	1.12E+00	4.01E+00	2.00E+00	up
Annulide	C15H20O2	Terpenoids	Sesquiterpenoids	9.00E+00	9.00E+00	9.00E+00	4.17E+03	3.52E+03	3.02E+03	1.20E+00	3.96E+02	8.63E+00	up
6,7-Dehydroartemisinic acid	C15H20O2	Terpenoids	Sesquiterpenoids	9.00E+00	9.00E+00	1.25E+03	9.00E+00	9.00E+00	3.11E+03	6.31E-02	2.47E+00	1.30E+00	insig
Costunolide	C15H20O2	Terpenoids	Sesquiterpenoids	4.43E+06	9.81E+06	7.61E+06	1.79E+05	1.47E+05	1.98E+05	1.19E+00	2.40E-02	-5.38E+00	down
Artemisinic acid	C15H22O2	Terpenoids	Sesquiterpenoids	9.00E+00	9.00E+00	9.00E+00	8.44E+04	7.11E+04	6.90E+04	1.20E+00	8.32E+03	1.30E+01	up
Aspergillusene A	C15H22O2	Terpenoids	Sesquiterpenoids	1.49E+04	1.29E+04	1.55E+04	1.75E+04	2.07E+04	2.40E+04	1.02E+00	1.43E+00	5.20E-01	insig
Arteannuin J	C15H22O2	Terpenoids	Sesquiterpenoids	2.02E+03	5.52E+03	5.19E+03	1.50E+03	1.50E+03	2.42E+03	8.86E-01	4.26E-01	-1.23E+00	insig
Neolinderane	C15H16O3	Terpenoids	Sesquiterpenoids	1.26E+06	4.63E+06	5.34E+06	9.00E+00	9.00E+00	9.00E+00	1.20E+00	2.41E-06	-1.87E+01	down
Lindenenol E	C15H18O3	Terpenoids	Sesquiterpenoids	2.16E+06	3.60E+06	4.12E+06	5.34E+03	1.47E+04	7.11E+03	1.19E+00	2.75E-03	-8.51E+00	down
Santonin	C15H18O3	Terpenoids	Sesquiterpenoids	4.17E+04	3.48E+05	4.03E+05	9.00E+00	9.00E+00	9.00E+00	1.19E+00	3.41E-05	-1.48E+01	down
Santamarin	C15H20O3	Terpenoids	Sesquiterpenoids	2.93E+06	8.36E+06	7.63E+06	4.03E+04	4.31E+04	3.72E+04	1.19E+00	6.37E-03	-7.29E+00	down
Reynosin	C15H20O3	Terpenoids	Terpene	9.00E+00	9.00E+00	9.00E+00	1.78E+04	9.42E+03	1.46E+04	1.20E+00	1.55E+03	1.06E+01	up
Parthenolide	C15H20O3	Terpenoids	Sesquiterpenoids	3.28E+04	2.90E+05	3.56E+05	9.45E+04	8.66E+04	6.25E+04	4.55E-01	3.59E-01	-1.48E+00	insig
Artemisinin B	C15H20O3	Terpenoids	Sesquiterpenoids	1.01E+06	1.20E+06	1.04E+06	9.00E+00	9.00E+00	9.00E+00	1.20E+00	8.29E-06	-1.69E+01	down
Magnolialide	C15H20O3	Terpenoids	Sesquiterpenoids	1.10E+04	1.76E+04	1.49E+04	9.00E+00	9.00E+00	9.00E+00	1.20E+00	6.21E-04	-1.07E+01	down
Cichoralexin	C15H20O3	Terpenoids	Sesquiterpenoids	2.26E+04	2.34E+04	3.47E+04	9.00E+00	9.00E+00	9.00E+00	1.20E+00	3.34E-04	-1.15E+01	down
Arteannuin N	C15H22O3	Terpenoids	Sesquiterpenoids	9.00E+00	9.00E+00	9.00E+00	1.65E+05	1.07E+05	1.34E+05	1.20E+00	1.50E+04	1.39E+01	up
Dihydro-epi-arteannuin B	C15H22O3	Terpenoids	Sesquiterpenoids	9.00E+00	9.00E+00	9.00E+00	3.82E+06	3.05E+06	2.78E+06	1.20E+00	3.57E+05	1.84E+01	up
Arteannuin L	C15H22O3	Terpenoids	Sesquiterpenoids	2.76E+05	5.19E+05	4.08E+05	7.65E+04	4.07E+04	4.02E+04	1.15E+00	1.31E-01	-2.93E+00	down
Arteannuin K	C15H22O3	Terpenoids	Sesquiterpenoids	2.70E+05	5.47E+05	4.13E+05	7.83E+04	4.83E+04	4.02E+04	1.15E+00	1.36E-01	-2.88E+00	down
4,5-Epoxyartemisinic Acid	C15H22O3	Terpenoids	Sesquiterpenoids	5.60E+04	5.10E+04	6.49E+04	4.02E+05	3.71E+05	4.06E+05	1.20E+00	6.86E+00	2.78E+00	up
Sclareolide	C16H26O2	Terpenoids	Ditepenoids	4.19E+05	2.10E+05	2.73E+05	5.78E+06	4.98E+06	5.62E+06	1.19E+00	1.82E+01	4.18E+00	up
Alpha-Epoxydihydroartemisinic Acid	C15H24O3	Terpenoids	Sesquiterpenoids	3.43E+04	6.40E+04	4.27E+04	3.04E+04	2.09E+04	2.73E+04	9.47E-01	5.57E-01	-8.44E-01	insig
8-Deoxylactucin	C15H16O4	Terpenoids	Sesquiterpenoids	2.22E+06	2.07E+06	2.05E+06	9.00E+00	9.00E+00	9.00E+00	1.20E+00	4.25E-06	-1.78E+01	down
Linderalactone C	C15H18O4	Terpenoids	Sesquiterpenoids	2.54E+03	1.04E+04	1.25E+04	9.00E+00	9.00E+00	9.00E+00	1.19E+00	1.06E-03	-9.88E+00	down
Lindenenol K	C15H20O4	Terpenoids	Sesquiterpenoids	1.75E+05	6.39E+05	5.82E+05	9.00E+00	9.00E+00	9.00E+00	1.20E+00	1.93E-05	-1.57E+01	down
Lactucin	C15H16O5	Terpenoids	Sesquiterpenoids	3.25E+04	2.68E+04	2.51E+04	9.00E+00	9.00E+00	9.00E+00	1.20E+00	3.20E-04	-1.16E+01	down
Lindenenol A	C15H18O5	Terpenoids	Sesquiterpenoids	1.08E+06	1.55E+06	2.12E+06	9.00E+00	9.00E+00	9.00E+00	1.20E+00	5.68E-06	-1.74E+01	down
Dihydro Artemisinin-D3	C15H24O5	Terpenoids	Sesquiterpenoids	9.00E+00	9.00E+00	9.00E+00	9.67E+03	9.07E+03	8.31E+03	1.20E+00	1.00E+03	9.97E+00	up
Mikanolide	C15H14O6	Terpenoids	Sesquiterpenoids	5.47E+04	2.69E+04	5.34E+04	9.00E+00	9.00E+00	9.00E+00	1.20E+00	2.00E-04	-1.23E+01	down
Blumeaene L	C17H26O5	Terpenoids	Sesquiterpenoids	7.87E+03	9.00E+00	9.00E+00	9.49E+03	8.93E+03	1.06E+04	8.69E-01	3.69E+00	1.88E+00	insig
Tussilagonone	C21H30O3	Terpenoids	Sesquiterpenoids	1.08E+04	1.37E+04	1.43E+04	2.76E+03	1.96E+03	1.01E+03	1.15E+00	1.48E-01	-2.76E+00	down
7β-Angeloyloxy-14-hydroxy-notonipetranone	C20H30O4	Terpenoids	Sesquiterpenoids	6.65E+04	5.43E+04	5.39E+04	2.11E+04	2.48E+04	2.11E+04	1.18E+00	3.84E-01	-1.38E+00	down
Moxartenolide	C20H22O5	Terpenoids	Sesquiterpenoids	8.57E+06	8.67E+06	8.17E+06	9.00E+00	9.00E+00	9.00E+00	1.20E+00	1.06E-06	-1.98E+01	down
6-DeoxyCatalpol	C15H22O9	Terpenoids	Terpene	1.02E+05	1.22E+05	1.22E+05	9.66E+04	7.74E+04	1.07E+05	8.19E-01	8.11E-01	-3.02E-01	insig
Argyinolide J	C20H28O5	Terpenoids	Sesquiterpenoids	6.70E+07	1.69E+05	1.19E+05	4.83E+03	1.44E+04	1.44E+04	8.98E-01	5.00E-04	-1.10E+01	insig
Balsamiferine E	C20H32O5	Terpenoids	Sesquiterpenoids	1.47E+06	2.05E+06	2.61E+06	5.76E+05	7.52E+05	7.24E+05	1.14E+00	3.35E-01	-1.58E+00	down
Sweroside	C16H22O9	Terpenoids	Sesquiterpenoids	2.19E+05	2.44E+05	2.04E+05	5.71E+05	5.09E+05	5.88E+05	1.19E+00	2.50E+00	1.32E+00	up
Ixoroside	C16H24O9	Terpenoids	Sesquiterpenoids	1.38E+05	1.05E+05	1.11E+05	1.47E+05	1.71E+05	1.39E+05	9.29E-01	1.29E+00	3.66E-01	insig
Argyinolide H	C20H28O6	Terpenoids	Sesquiterpenoids	1.78E+04	2.26E+04	2.58E+04	9.00E+00	9.00E+00	9.00E+00	1.20E+00	4.08E-04	-1.13E+01	down
Citroside A	C19H30O8	Terpenoids	Terpene	1.49E+06	9.32E+05	9.66E+05	4.42E+05	7.68E+05	5.40E+05	1.01E+00	5.17E-01	-9.52E-01	insig
Geniposide	C17H24O10	Terpenoids	Monoterpenoids	3.00E+05	2.76E+05	2.18E+05	3.32E+05	3.58E+05	3.57E+05	9.90E-01	1.32E+00	4.02E-01	insig
Ixerisoside D	C21H28O8	Terpenoids	Sesquiterpenoids	6.44E+04	1.19E+05	1.91E+05	5.46E+05	3.22E+05	4.65E+05	1.06E+00	3.56E+00	1.83E+00	up
3,4β-Dihydro-15-dehydrolactucopicrin	C23H22O7	Terpenoids	Sesquiterpenoids	9.00E+00	5.97E+03	4.91E+03	1.69E+04	1.71E+04	8.04E+03	6.91E-01	3.86E+00	1.95E+00	insig
Lactucopicrin	C23H22O7	Terpenoids	Sesquiterpenoids	6.88E+03	1.23E+04	1.08E+04	9.00E+00	9.00E+00	9.00E+00	1.20E+00	9.02E-04	-1.01E+01	down
Sonchuside A	C21H32O8	Terpenoids	Sesquiterpenoids	1.54E+04	1.07E+04	1.38E+04	2.60E+04	4.13E+04	2.62E+04	1.09E+00	2.34E+00	1.23E+00	up
Sonchuside G	C21H34O8	Terpenoids	Sesquiterpenoids	9.00E+00	9.00E+00	9.00E+00	3.26E+04	1.89E+04	3.46E+04	1.20E+00	3.19E+03	1.16E+01	up
Sonchuside E	C21H30O9	Terpenoids	Sesquiterpenoids	1.41E+04	1.48E+04	1.19E+04	1.47E+04	1.94E+04	1.65E+04	8.49E-01	1.24E+00	3.10E-01	insig
Ixerin D	C21H30O9	Terpenoids	Sesquiterpenoids	1.94E+04	2.31E+04	2.22E+04	2.40E+04	2.67E+04	2.79E+04	9.74E-01	1.21E+00	2.80E-01	insig
Asperulosidic acid	C18H24O12	Terpenoids	Monoterpenoids	9.00E+00	9.00E+00	9.00E+00	1.19E+05	1.16E+05	8.85E+04	1.20E+00	1.20E+04	1.35E+01	up
Sarcaglaboside F	C21H28O10	Terpenoids	Sesquiterpenoids	3.83E+04	4.62E+04	3.85E+04	9.00E+00	9.00E+00	9.00E+00	1.20E+00	2.20E-04	-1.22E+01	down
Shionoside A	C21H36O10	Terpenoids	Monoterpenoids	9.00E+00	9.00E+00	9.00E+00	2.90E+04	2.31E+04	3.74E+04	1.20E+00	3.31E+03	1.17E+01	up
5-Hydroxy-10-O-(p-methoxycinnamoyl)adoxosidic acid	C25H30O13	Terpenoids	Monoterpenoids	5.64E+03	6.54E+03	9.59E+03	7.25E+03	7.43E+03	7.12E+03	9.28E-02	1.00E+00	2.51E-03	insig
Ixerisoside N	C27H42O12	Terpenoids	Sesquiterpenoids	5.03E+03	5.38E+03	7.73E+03	9.00E+00	9.00E+00	9.00E+00	1.20E+00	1.49E-03	-9.39E+00	down
Arteminolide B	C35H40O8	Terpenoids	Sesquiterpenoids	1.04E+07	1.06E+07	9.47E+06	9.00E+00	9.00E+00	6.76E+03	1.12E+00	2.22E-04	-1.21E+01	down
Arteminolide A	C35H42O8	Terpenoids	Sesquiterpenoids	5.72E+06	6.26E+06	4.91E+06	9.00E+00	9.00E+00	9.00E+00	1.20E+00	1.60E-06	-1.93E+01	down
3-O-(2-O-Acetyl-glucosyl)oleanolic acid	C38H60O10	Terpenoids	Triterpene	2.17E+06	2.93E+06	3.61E+06	1.02E+06	1.13E+06	9.38E+05	1.15E+00	3.54E-01	-1.50E+00	down
Ecliptasaponin IX	C36H62O11S	Terpenoids	Triterpene	3.98E+04	4.90E+04	4.50E+04	9.00E+00	9.00E+00	9.00E+00	1.20E+00	2.02E-04	-1.23E+01	down
Oleanolic acid-3-O-xylosyl(1→3)glucuronide	C41H64O13	Terpenoids	Triterpene Saponin	7.07E+03	2.22E+05	3.02E+05	9.00E+00	9.00E+00	9.00E+00	1.16E+00	5.09E-05	-1.43E+01	down
