# Supplementary material for: Metabolomic and transcriptomic analyses provide insights into variations in flavonoids contents between two Artemisia cultivars
Source: BMC Plant Biol. 2023 May 30;23:288. doi: 10.1186/s12870-023-04295-8 (PMC10228080; doi:10.1186/s12870-023-04295-8)
Supplement: Supplementary file 3 — Additional file 3: Table S2. qRT-PCR primers used for validation different expressed genes between NYSY and NYYY. [file 12870_2023_4295_MOESM3_ESM.pdf]

Table S2. qRT-PCR primers used for validation different expressed genes between NYSY and NYYY

Gene name	Gene cluster	Forward Primer (5'→3')	Reverse Primer (5'→3')
AaActin	12810.14761	GTCACACTGGTGTCATGGTTGGAAT	CGTGCTCAATCGGGTACTTCAAAGT
AaCHI	12810.21667	ATTAATGCCAGGCGGTGGAT	CCTAGCGCTCCTTCCAACAT
AaCHS	12810.17019	TCGCCAGGATGTGTTAGTCG	CTGGATTTCGGTTGTCCCCA
AaF3'H	12810.6764	GTACTTTACGCCGTCGTCCT	GCATCAATGGCCCGTACTT
AaFSII	12810.21356	TCGTCTACCGCCTAGTCCTC	GGACCCTAGACGAAGGTGGA
AaF3H	12810.17316	CGGCCTAAGGTGCCATACAA	TGTCCACGCCATGATCAACA
AaI2'H	12810.21024	ACCACACGAGCCATCAGAAG	GGGTCCCTATGGATAGCCCA
